# Cryogenically Flexible Organic Single-Crystal Heterostructures via Molecular Alloying for Advanced Optical Tuning

**DOI:** 10.1021/jacs.6c14819

**Published:** 2026-10-27

**Authors:** Wenbo Wu, Yifang Lu, Mingliang Wu, Jiaxuan Zhu, Hui Yu, Xin Huang, Na Wang, Qiang Li, Ting Wang, Dongyang Zhu, Panče Naumov, Hongxun Hao

**Affiliations:** † National Engineering Research Center of Industrial Crystallization Technology, School of Chemical Engineering and Technology, 12605 Tianjin University, Tianjin 300072, P. R. China; ‡ 12579 Suzhou Laboratory, Suzhou, Jiangsu 215123, P. R. China; § Engineering Research Center of Green Refining Process, Ministry of Education, Tianjin University, Tianjin 300072, P. R. China; ∥ Key Laboratory of Quantum Materials and Devices of Ministry of Education, School of Physics, 597765 Southeast University, Nanjing 211189, P. R. China; ⊥ Smart Materials Lab, New York University Abu Dhabi, Abu Dhabi 129188, UAE; # Center for Smart Engineering Materials, New York University Abu Dhabi, Abu Dhabi 129188, UAE; ∇ Research Center for Environment and Materials, Macedonian Academy of Sciences and Arts, Bul. Krste Misirkov 2, Skopje MK−1000, Macedonia; ○ Molecular Design Institute, Department of Chemistry, New York University, 100 Washington Square East, New York, New York 10003, United States

## Abstract

Organic single-crystal heterostructures (OSCHs) constitute a promising platform for advanced flexible optoelectronics, yet imparting mechanical flexibility to these multiblock systems, especially given the susceptibility to failure at the interfaces between individual crystal segments, remains challenging. This obstacle is particularly aggravated at cryogenic temperatures, where the freezing of vibrational motion may cause brittleness and lead to mechanical failure. Herein, we describe an epitaxial growth strategy using molecular alloys for the fabrication of crystals of cryogenically flexible two-color (blue–cyan–blue) triblock heterostructures, prepared by growing triphenylene (TP) segments onto perylene (PER)-doped alloy seed crystals via crystallization under controlled cooling. The compatibility between the host and guest molecules provides interfaces with low defect densities. Analysis of the crystal structure reveals an abundance of weak intermolecular interactions that are thought to enable the reversible elastic deformation observed at both 298 and 77 K, thereby preventing cracking. Simultaneously, efficient Förster resonance energy transfer (FRET) between TP and PER enables continuous emission tuning and doping-ratio-controlled programmability of the optoelectronic properties. The resulting heterostructures not only support low-loss active optical waveguiding and programmable multichannel optical logic and information encoding but also remain fully operational for optical transduction during bending under cryogenic conditions. This work establishes a general molecular alloying approach to structures that integrate low-temperature mechanical compliance with sophisticated photonic functionalities, providing a pathway to deformable, multiemissive optoelectronics elements that remain operational in extreme environments.

## Graphical abstract

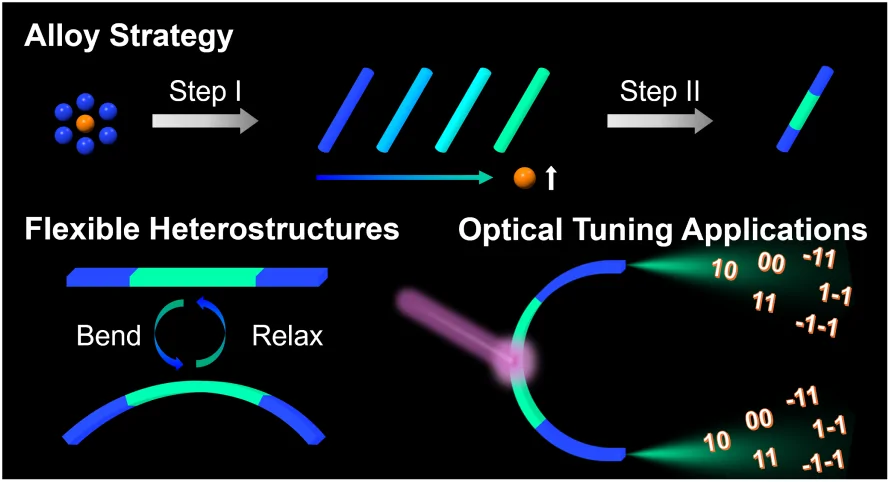

## Introduction

Organic single-crystal heterostructures (OSCHs) have been recognized as key materials for advanced photonic and optoelectronic devices^1–3^ such as optical waveguides,^4–6^ logic gates,^7–9^ and multiwavelength signal processors.^10–12^ These structures benefit from long-range molecular ordering, low defect densities, and controllable interfacial energy and charge transfer.^13–15^ However, the practical application of conventional OSCHs is limited by the perceived intrinsic mechanical brittleness of organic single crystals,^16,17^ which arises from their highly ordered and densely packed crystalline lattice.^18,19^ This brittleness restricts their integration into flexible or deformable architectures,^20–23^ particularly under extreme conditions, such as cryogenic temperatures.^24,25^ Recent advances in dynamic adaptive organic crystals^26^ that build on molecular-scale dynamics^27^ have demonstrated that organic single crystals can exhibit mechanical elasticity,^28–31^ even at cryogenic temperatures,^32–34^ thereby challenging the conventional perception of their inherent rigidity. These developments lay a foundation for single-crystal organic materials that combine structural adaptability, low-temperature functionality, and high optical performance.^35,36^ Nevertheless, the realization of such mechanical compliance with multiblock OSCHs remains a significant challenge—the formation of high-quality OSCHs relies on a high degree of lattice matching between coexisting phases to ensure coherent, seamless interfaces,^37–39^ while maintaining mechanical integrity at the heterojunction demands sufficient intrinsic toughness in each phase to withstand bending stress.^40,41^

The existing strategies for the construction of flexible heterostructures, such as employing photochromic transformations,^42^ acidochromic switches,^43^ or polymorphic transitions^44^ typically involve local stimulation of only one section of a crystal (e.g., irradiation or chemical treatment of one end or a specific region). While the stimulated region eventually undergoes structural or chemical changes to form a heterojunction with the untreated part, this approach often compromises the integrity of the crystal; the localized transformation frequently introduces defects such as microcracks, lattice distortions, or even delamination at the interface, resulting in degraded mechanical and optical performance upon repeated deformation.^42–44^ More critically, the mechanical behavior of OSCHs at cryogenic temperatures remains largely unexplored, creating a critical knowledge gap that hinders their application in space exploration, polar instrumentation, and other extreme-environment optoelectronics. Therefore, it is of considerable importance to develop strategies that combine cryogenic mechanical compliance and sophisticated multichannel photonic functionalities in the same OSCH.

To address these challenges, we employed a molecular alloying strategy (also referred to as mixed crystals or solid solutions), previously demonstrated to be effective for constructing high-quality heterostructures (Figure 1a).^37,45,46^ This approach involves incorporating a small fraction of geometrically and dimensionally compatible guest molecules (e.g., perylene, PER) into a host lattice (e.g., triphenylene, TP) to prepare a homogeneous solid solution with near-perfect lattice continuity. Building on recent progress in cryogenically flexible single crystals,^32^ we extended this molecular alloying platform to multiblock OSCHs through seed-induced heteroepitaxial growth. This alloying approach offers several key advantages over traditional methods. First, it provides superior lattice matching.^47^ Unlike conventional heterojunctions between dissimilar molecules, which typically suffer from large lattice mismatches and high defect densities,^48–50^ the host–alloy boundary forms a homoepitaxial-like interface.^45^ This minimizes structural defects and interfacial strain, enabling precise construction of heterojunctions with well-defined spatial topologies.^46^ Second, the strategy ensures cryogenic elasticity and crack resistance.^51^ By incorporating only a small fraction of guest molecules, the alloying approach preserves the intrinsic mechanical flexibility of the host crystal,^52^ effectively suppressing the flexible-to-brittle transition at low temperatures. Third, it allows simultaneous tuning of optoelectronic properties without compromising the mechanical framework.^53–56^ Optical functionalities (e.g., emission color via energy transfer) can be continuously modulated by varying the doping ratio,^54^ eliminating the need for de novo molecular design or complex synthesis. Finally, the resulting heterostructures exhibit high durability and straightforward programmability.^52^ In contrast to fatigue-prone stimuli-responsive methods, this strategy preserves long-range crystalline order and avoids structural phase transitions, offering a reliable, device-integrable materials platform.

**Figure 1:**
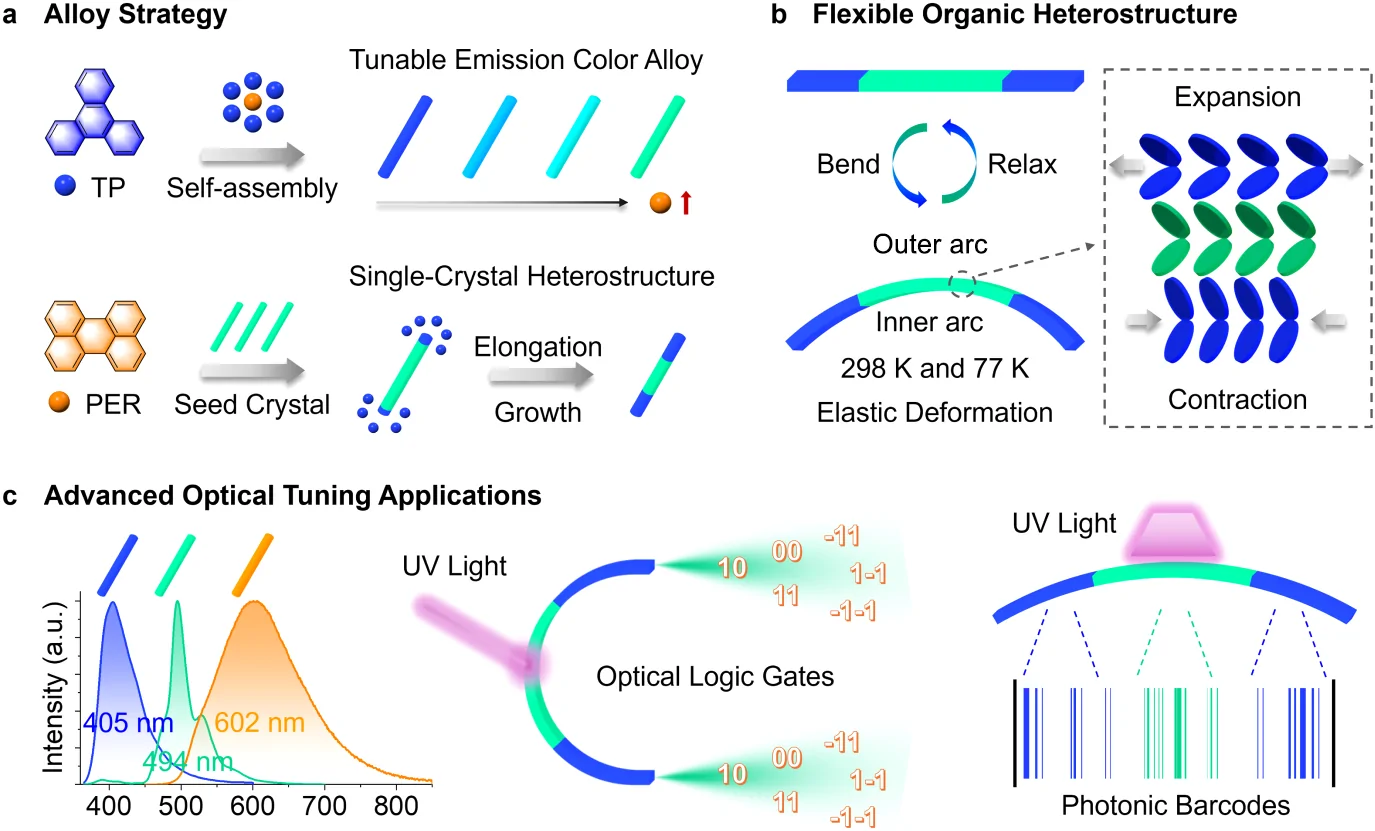
**Concept of flexible molecular alloy crystalline heterostructures for optical transmission and optical logic gate applications.** (a) Schematic illustration of the concept, heteroepitaxial growth, and structural assembly of the triblock OSCHs. (b) Proposed mechanism and molecular-level lattice response during the reversible elastic bending of alloy crystals at 298 and 77 K. (c) Photonic and optoelectronic applications of flexible OSCHs, including active optical waveguiding, multichannel logic operations, and information encoding.

Herein, we use an alloy epitaxial approach to fabricate cryogenically flexible blue–cyan–blue triblock OSCHs (Figure 1). Elastically compliant TP segments were grown onto PER-doped alloy seed crystals through controlled crystallization by cooling, yielding well-defined triblock OSCHs with coherent interfaces (Figure 1a). Single-crystal X-ray diffraction, Hirshfeld surface analysis, and energy framework calculations indicate that a network of weak intermolecular interactions (primarily C–H···H/C and π–π stacking) supports the reversible elastic deformation observed at both 298 and 77 K and prevents crack formation (Figure 1b). Concurrently, efficient Förster resonance energy transfer (FRET) within the alloy blocks enables continuous emission tuning and programmability of the optoelectronic properties, which can be controlled by varying the doping ratio without invoking complex synthetic protocols. The resulting heterostructures demonstrate low-loss active optical waveguiding and enable programmable multichannel optical logic and information encoding, and remain operational even when the materials are bent under cryogenic conditions (Figure 1c).

## Results and Discussion

### Fabrication and Characterization of the Molecular Alloys

TP and PER were selected as the host and guest, respectively, for the preparation of molecular alloys. Pure TP single crystals exhibit bluish-purple fluorescence (λ_em_ = 405 nm), while pure PER single crystals exhibit orange fluorescence (λ_em_ = 602 nm) (Figure 1c and Figure S1). Formation of a homogeneous solid solution requires compatibility between the host and guest molecules.^54^ The approximate lateral and longitudinal molecular dimensions of TP are 11.52 Å and 11.04 Å, respectively, while those of PER are 11.69 Å and 9.15 Å (Figures 2a,b and Figure S2).^57^ Both molecules also have high axial symmetry and self-assemble in the respective structures by π–π stacking interactions at comparable distances (3.75 Å for TP and 3.86 Å for PER; Figure S3 and Table S1). These structural similarities provide a strong basis to ensure isomorphous coassembly,^52^ and, indeed, we were able to prepare single crystals with composition TP_1–*x*_PER_*x*_ (where *x* is the feed molar fraction of PER, 0 < *x* < 1) by controlled cooling of acetonitrile solutions. Needle-like alloy crystals with relatively uniform morphology were obtained at feed ratios *x* ≤ 0.10 (Figure S4).

**Figure 2:**
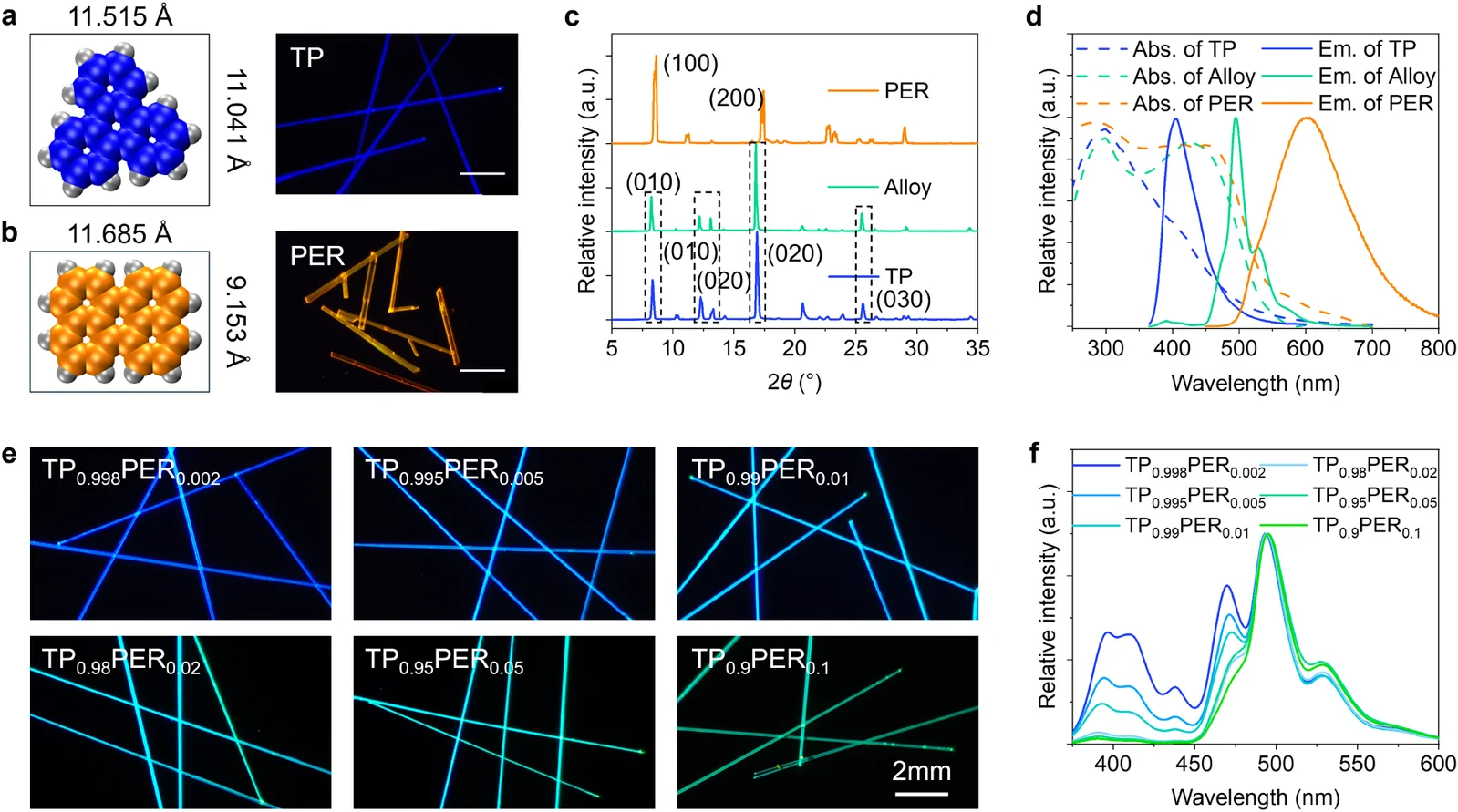
**Structural and photophysical characterization of the TP**_**1‑** ***x***_ **PER*_x_*** **alloy crystals.** (a, b) Molecular structures, dimensions, and PL photographs of pure TP (a) and pure PER (b) single crystals (scale bars: 1 mm). (c) PXRD patterns of pure PER (orange), alloy (green), and pure TP (blue) crystals. (d) UV–vis absorption (dashed lines) and steady-state PL spectra (solid lines) of pure TP, pure PER, and the alloy crystals, highlighting the spectral overlap between host emission and guest absorption. (e, f) PL photographs (e) and corresponding PL spectra (f) of alloy single crystals prepared with different PER feed fractions (*x*).

Spatially resolved photoluminescence (PL) spectra recorded across different regions of an individual alloy crystal were nearly identical, confirming compositional and spectroscopic homogeneity (Figure S5). In contrast, phase separation occurred at feed ratios of *x* > 10%, resulting in a mixture of orange plate-like aggregates and green needle-like crystals (Figure S6). Interestingly, attempts to use PER as the host for TP doping under identical crystallization conditions did not yield the expected solid solutions. This result is attributed to the denser molecular packing of PER relative to TP, which probably hinders incorporation of guest molecules. This conclusion was supported by theoretical calculations: the π–π stacking interaction energy of pure PER (*E*_PER_ = 77.3 kJ mol^–1^) is substantially higher than that of TP (*E*_TP_ = 46.1 kJ mol^–1^), and the lattice free volume of PER (25.1%) is smaller than that of TP (29.6%) (Figure S7).^57^ Quantification of the crystal composition revealed that the feed ratio (*x*) deviates from the actual doping ratio (*y*). Specifically, for a feed ratio of *x* = 0.1, and thus nominal composition TP_0.9_PER_0.1_, ^1^H NMR spectroscopy of the dissolved crystals in CDCl_3_ yielded an actual PER incorporation ratio (*y*) of approximately 0.5% (Figure S8). The structural phase and purity of the crystalline molecular alloys were confirmed by powder X-ray diffraction (PXRD) and differential scanning calorimetry (DSC). The PXRD patterns display diffraction peaks at 2θ = 8.3°, 12.3°, 13.3°, 16.9°, 20.7°, and 25.6°, which match those of the pure TP phase, indicating preservation of the host crystal lattice (Figures 2c and Figure S9a). Concurrently, the DSC profiles show that the melting temperature of the alloy closely matches that of pure TP, consistent with low dopant incorporation (Figure S9b).

The emission color of the alloy crystals varies with the concentration of the guest molecule (PER).^58^ As shown in Figure 2d, the steady-state PL spectrum of pure TP crystals (365–600 nm) exhibits substantial spectral overlap with the ultraviolet–visible (UV–vis) absorption spectrum of PER crystals (250–700 nm), which enables efficient FRET from TP to PER.^54,56^ Consequently, systematic variation of the PER feed ratio allows continuous tuning of the emission color from blue to green (Figure 2e). UV–vis absorption spectra show progressive intensification of the characteristic PER absorption band at 430 nm with increasing dopant concentration (Figure S10). Meanwhile, under ultraviolet excitation, the emission peaks at 396 and 410 nm, which are characteristic of TP crystals, undergo continuous quenching with the incremental incorporation of PER components into the alloy crystals. This behavior is attributed to a FRET process: higher PER content facilitates energy transfer from TP to PER, thereby attenuating the intrinsic emission of TP (Figure 2f).

At low PER concentrations (e.g., TP_0.998_PER_0.002_), the alloy crystals exhibit four prominent emission peaks (396, 410, 470, and 493 nm), corresponding to Commission Internationale de l’Éclairage (CIE) coordinates of (0.15, 0.27). At higher doping levels (e.g., TP_0.9_PER_0.1_), the host emission is substantially quenched, and the spectrum is dominated by guest emission bands at 494 and 527 nm, shifting the CIE coordinates to (0.16, 0.47) (Figure S11).

Maximizing the absolute photoluminescence quantum yield (Φ_F_, PLQY) is critical for minimizing propagation losses in active optical waveguiding applications.^59^ We therefore examined the effect of PER guest doping on the radiative efficiency of the alloy crystals. The FRET efficiency was determined from the fluorescence lifetimes of the donor using the relation FRET­(*E*_*p*_) = 1 – τ_DA_/τ_D_, where τ_DA_ and τ_D_ are the lifetimes of the host in the presence and absence of the acceptor, respectively (Figure S12 and Table S2).^54^ The calculated FRET values range from 57% to 64% (Table S2). This efficient energy transfer process optimizes exciton utilization, resulting in absolute Φ_*F*_ values for the alloy crystals that remain high and, in some cases, exceed that of the pure TP host (pure TP: Φ_F_ = 0.59; pure PER: Φ_F_ = 0.05; TP_0.9_PER_0.1_: Φ_F_ = 0.66; Figure S12 and Table S2). To understand the origin of this enhancement, the radiative (*k*_r_) and nonradiative (*k*_nr_) decay rate constants were calculated from the measured Φ_F_ and average lifetime (τ) values (Table S2). Compared with pure TP (*k*_r_ = 0.21 × 10^8^ s^–1^, *k*_nr_ = 0.15 × 10^8^ s^–1^) and pure PER (*k*_*r*_ = 0.0029 × 10^8^ s^–1^, *k*_nr_ = 0.054 × 10^8^ s^–1^), the TP_0.9_PER_0.1_ alloy exhibits a significantly suppressed nonradiative decay rate (*k*_nr_ = 0.053 × 10^8^ s^–1^) while retaining a comparable radiative rate (*k*_r_ = 0.10 × 10^8^ s^–1^). This suppression of nonradiative pathways upon alloying accounts for the enhanced quantum yield and demonstrates that the solid-solution strategy simultaneously achieves emission color tunability and improved luminescent performance.

While the exceptional mechanical flexibility of pure TP crystals at both room temperature and cryogenic conditions has been well documented (Figure S13),^32,60^ the mechanical compliance of the multicomponent alloy crystals requires systematic evaluation. Large-aspect-ratio, centimeter-scale mixed single crystals were readily prepared by crystallization upon cooling (Figure S14). At room temperature, individual TP_0.9_PER_0.1_ crystals exhibited reversible elastic bending into a U-shape under applied shear stress using tweezers, with full recovery to the original linear morphology upon stress release (Figure 3a). Moreover, with one end fixed, the crystals could be coiled into a closed loop using a microneedle (Movie S1). Similar elastic behavior was observed across all compositions of the mixed crystals (Figure 3b and Movie S2). Scanning electron microscopy (SEM) images of the crystal surfaces after 50 bending–recovery cycles showed no detectable microcracks or structural defects, demonstrating the excellent fatigue resistance of the alloy crystals (Figure S15). Importantly, reversible elastic deformation was retained not only at 77 K but also at elevated temperatures up to 433 K, demonstrating remarkable mechanical robustness of the alloy crystals across a broad, experimentally accessible temperature range (Figure 3c,d, Figure S16a–c, and Movie S3).

**Figure 3:**
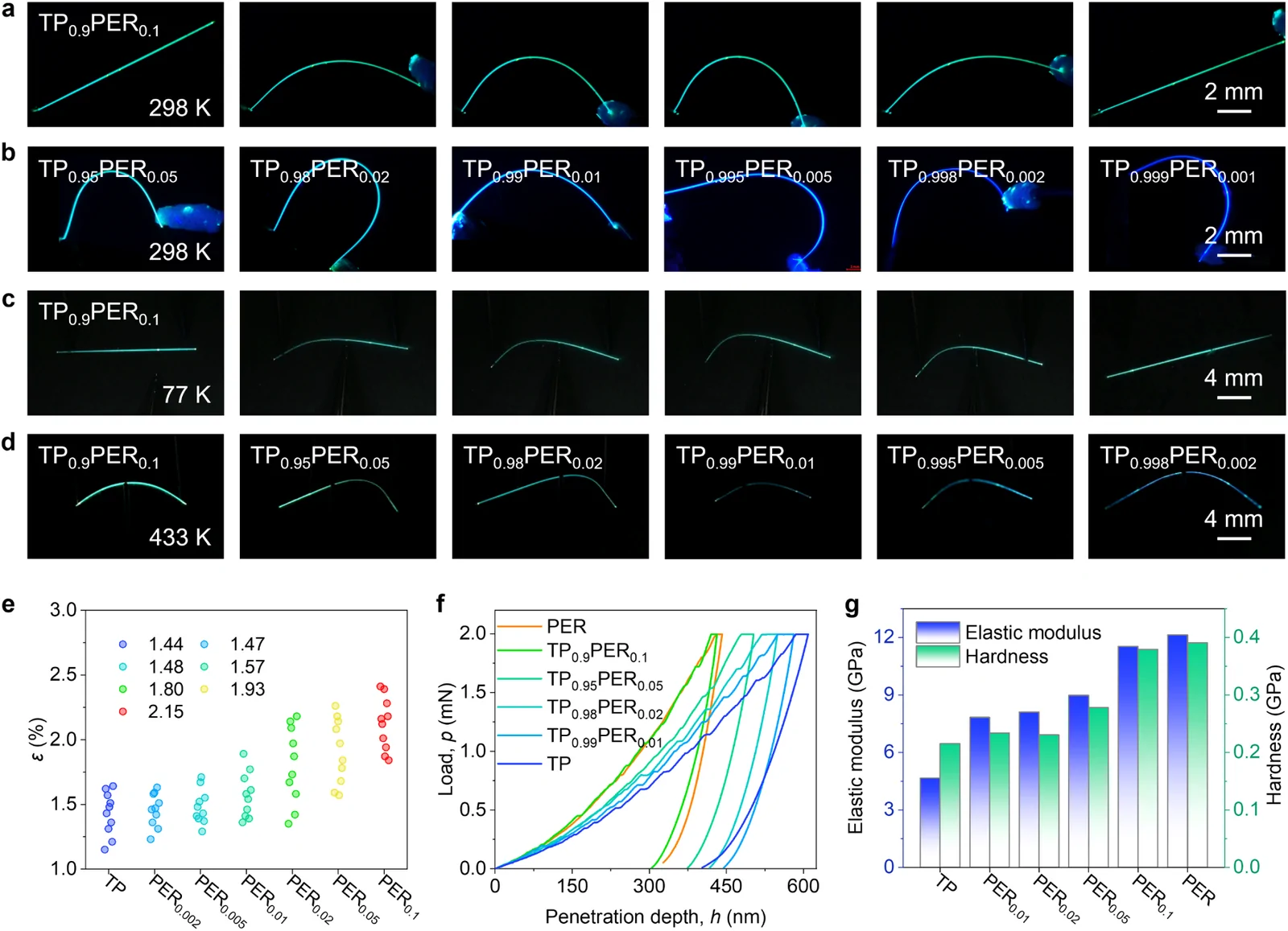
**Mechanical properties of the TP**_**1‑** ***x***_ **PER*_x_*** **alloy single crystals.** (a–d) Reversible elastic deformation of alloy single crystals at 298 K (a, b), 77 K (c), and 433 K (d). (e) Statistical analysis of the maximum elastic strain (*ε*) as a function of alloy crystals. (f) Representative load–displacement (*P*–*h*) curves of pure TP, alloy, and pure PER crystals recorded at a maximum load of 2 mN. (g) Comparison of the *E* and *H* values extracted from nanoindentation measurements for pure TP, the alloy, and pure PER crystals.

To quantify this elasticity, the maximum elastic strain (*ε*) was measured across different alloy crystals, with ten independent measurements per composition. At room temperature, the average elastic strain of pure TP crystals was 1.44 ± 0.28%, whereas that of TP_0.9_PER_0.1_ crystals reached 2.15 ± 0.45% (Figure 3e). At 77 K, the corresponding values decreased to 1.00 ± 0.24% and 1.38 ± 0.33%, respectively (Figure S16d). Although the average elastic strain decreases upon cooling, the composition-dependent enhancement induced by PER incorporation is retained at 77 K, demonstrating that molecular alloying continues to improve lattice compliance under cryogenic conditions. Nanoindentation measurements were subsequently performed to quantify the surface mechanical properties, specifically the elastic modulus (*E*) and hardness (*H*) (Figure 3f and Table S3). The average *E* values for pure TP, TP_0.9_PER_0.1_ alloy, and pure PER were determined to be 4.65 ± 0.37, 11.52 ± 0.15, and 12.13 ± 0.12 GPa, respectively, with corresponding *H* values of 0.22 ± 0.008, 0.38 ± 0.011, and 0.39 ± 0.012 GPa (Figure 3g). Both *E* and *H* of the alloy crystals increase with higher PER feed fractions, approaching the values of the pure PER phase. This trend is attributed to the denser structural packing induced by the presence of guest molecules, which mechanically reinforces the lattice while preserving the stacking systems required for elastic compliance.

### Fabrication and Characterization of Flexible OSCHs

TP and its alloy crystals exhibit excellent elasticity, which motivated us to fabricate flexible OSCHs based on these materials. Centimeter-scale blue–cyan–blue triblock OSCHs were successfully prepared via the seed-induced heteroepitaxial growth strategy (Figure 4a).^61^ Specifically, pregrown TP_0.9_PER_0.1_ alloy single crystals were first obtained by cooling crystallization and then introduced as heteromolecular seeds into a saturated acetonitrile solution of TP at 303.15 K. Slow cooling of the mixture to 273.15 K induced localized secondary nucleation and epitaxial growth from the termini of the seed crystals. After several hours, well-defined centimeter-scale triblock OSCHs, typically 2–5 cm in length, were isolated (Figures 4b and Figure S17). Upon UV excitation (λ_ex_ = 365 nm), the two end sectors were observed to emit blue fluorescence, while the central sector exhibited cyan emission (Figure 4c,d).

**Figure 4:**
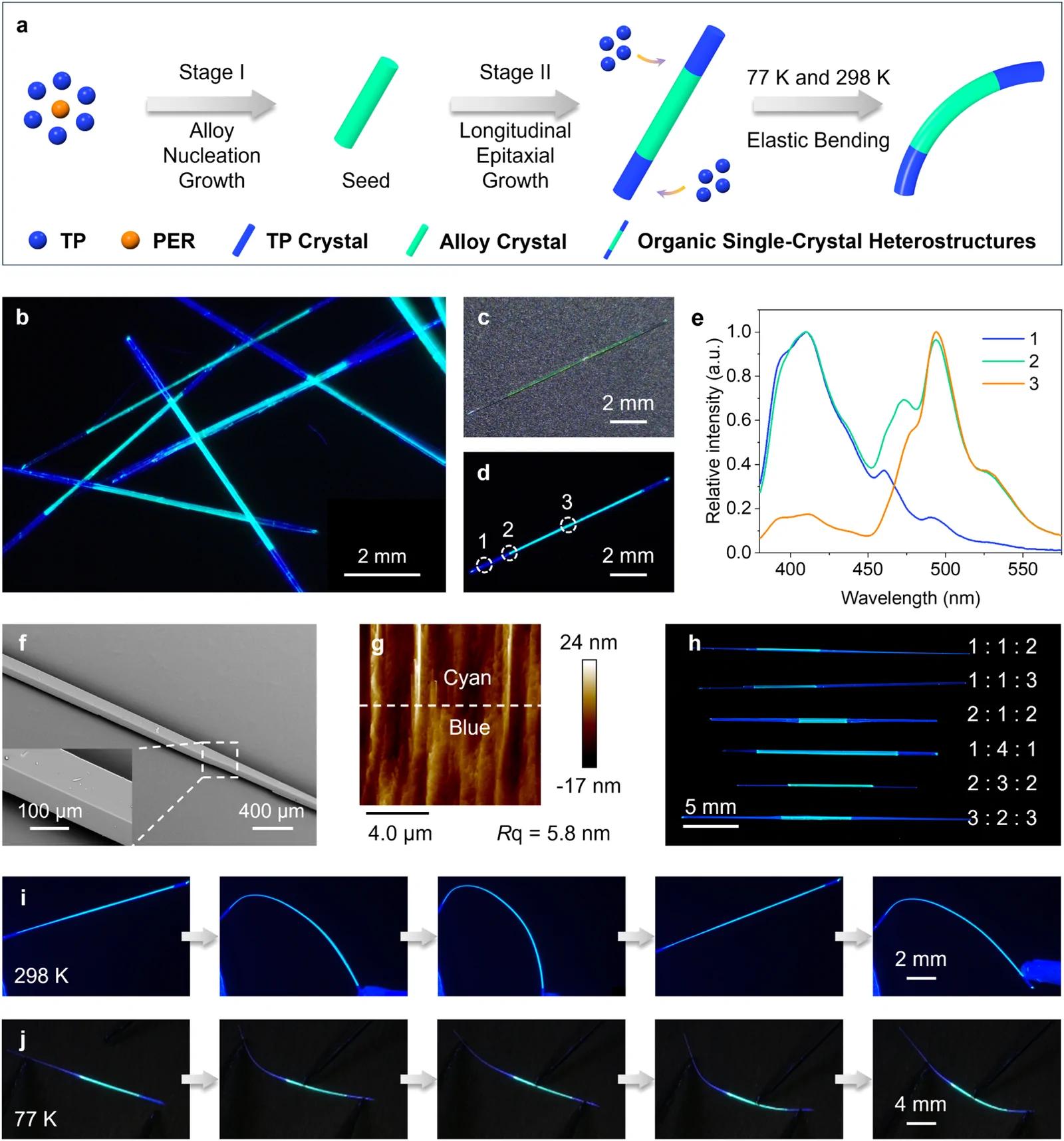
**Preparation, optical properties, and mechanical properties of flexible blue–cyan–blue triblock OSCHs.** (a) Schematic illustration of the seed-induced heteroepitaxial growth process for fabricating triblock OSCHs. (b–d) Optical photographs of centimeter-scale triblock OSCHs obtained by cooling crystallization under UV illumination (b, d) and daylight (c). (e) Spatially resolved photoluminescence (PL) spectra collected from different positions marked on a representative OSCH. (f, g) SEM (f) and AFM (g) images of the heterojunction interface in the OSCHs. (h) PL photographs of triblock OSCHs with different block length ratios. (i, j) Reversible elastic bending deformation of triblock OSCHs at 298 K (i) and 77 K (j).

To elucidate the interfacial characteristics and structural homogeneity of the heterojunctions, spatially resolved PL spectroscopy, SEM, and atomic force microscopy (AFM) were employed. As shown in Figure 4e, the outer blue-emitting domains exhibited a primary emission maximum at 409 nm, matching the photophysical characteristics of pure TP single crystals. The central cyan-emitting segment displayed a dominant emission peak at 494 nm, identical to that of TP_0.99_PER_0.01_ alloy crystals. At the heterojunction boundary, the emission spectrum showed a dual-peak profile with comparable intensities at 409 and 494 nm, indicating a highly coherent and optically coupled interface. PL spectra acquired at multiple positions within both the terminal and middle segments were nearly identical, confirming compositional uniformity within each domain (Figure S18). The corresponding CIE chromaticity coordinates were (0.16, 0.07) for the terminal segments and (0.16, 0.39) for the central segment, in excellent agreement with those of the parent TP and TP_0.99_PER_0.01_ crystals, respectively (Figures S11 and S19).

Microstructural and morphological analyses further confirmed the high quality and integrity of the heterostructures. SEM images revealed a continuous, crack-free surface across the entire triblock OSCH, with no discernible phase boundaries or delamination (Figure 4f), consistent with the excellent lattice matching between the TP and alloy segments. AFM topographic profiles demonstrated smooth interfacial connectivity, with a low root-mean-square (RMS) roughness of approximately 5.8 nm at the interfaces (Figures 4g and Figure S20). To optimize reproducibility and morphological uniformity for potential scale-up, the effects of crystallization conditions were systematically investigated. Acetonitrile was identified as the optimal solvent, with an initial saturation temperature of 303.15 K and a cooling rate of 10 K h^–1^ providing the highest structural fidelity (see Fabrication of Crystals in the Supporting Information).

The ability to precisely control the sector compositional ratios in OSCHs is of significant practical importance for tailoring their photonic performance.^9^ To that end, we developed a simple top-down strategy that exploits the excellent mechanical compliance of the alloy segments. Owing to their robust lattice, the alloy crystals can be mechanically cleaved along specific crystallographic planes without fracturing, microcracking, or loss of single-crystal integrity.^62^ Consequently, triblock OSCHs with arbitrary block length ratios, as well as diblock heterostructures, can be readily fabricated by controlled sectioning (Figure 4h and Figures S21–S24). The resulting triblock OSCHs retain the intrinsic mechanical resilience of their constituent materials. They exhibit fully reversible elastic bending deformation at room temperature (Figure 4i and Movie S4) as well as under cryogenic conditions (77 K, Figure 4j and Movie S5). However, three-point bending tests revealed that fracture tends to occur at the heterojunction interface when the stress is applied directly at the interface (Movie S5). The maximum elastic strain of the OSCHs (approximately 1–1.4%) is slightly lower than that of the pure TP or alloy crystals. This modest reduction is likely attributable to the formation of grain boundaries during epitaxial growth, which slightly weakens the interfacial interactions between the TP and alloy segments.^63^

### Mechanistic Elucidation of Elastic Bending under Cryogenic Conditions

To elucidate the structural origin of the observed mechanical flexibility, the crystal structure of TP_0.9_PER_0.1_ was determined by single-crystal X-ray diffraction. At 298 K, the alloy crystallizes in the orthorhombic space group *P*2_1_2_1_2_1_, *Z* = 4, and unit cell parameters *a* = 5.2702(3) Å, *b* = 13.1718(8) Å, and *c* = 16.7469(9) Å (Figure 5a). These unit cell parameters and the overall molecular packing are highly consistent with those of the pure TP crystal (Figure S25 and Table S1),^32^ providing excellent lattice matching that facilitates epitaxial growth of TP on alloy seeds. Upon cooling to 100 K, the crystal symmetry of TP_0.9_PER_0.1_ is preserved, but the unit cell contracts anisotropically, with the final values for *a* = 5.2639(3) Å, *b* = 12.8914(4) Å, and *c* = 16.6385(7) Å, corresponding to contractions of 0.12%, 2.13%, and 0.65% along the *a*-, *b*-, and *c*-axes, respectively (Figure 5a and Table S1). The molecular stacking distance concomitantly decreases slightly, from 3.745 Å to 3.702 Å. Crystal face indexing confirmed that bending occurs on the (011̅) face (Figure S26).

**Figure 5:**
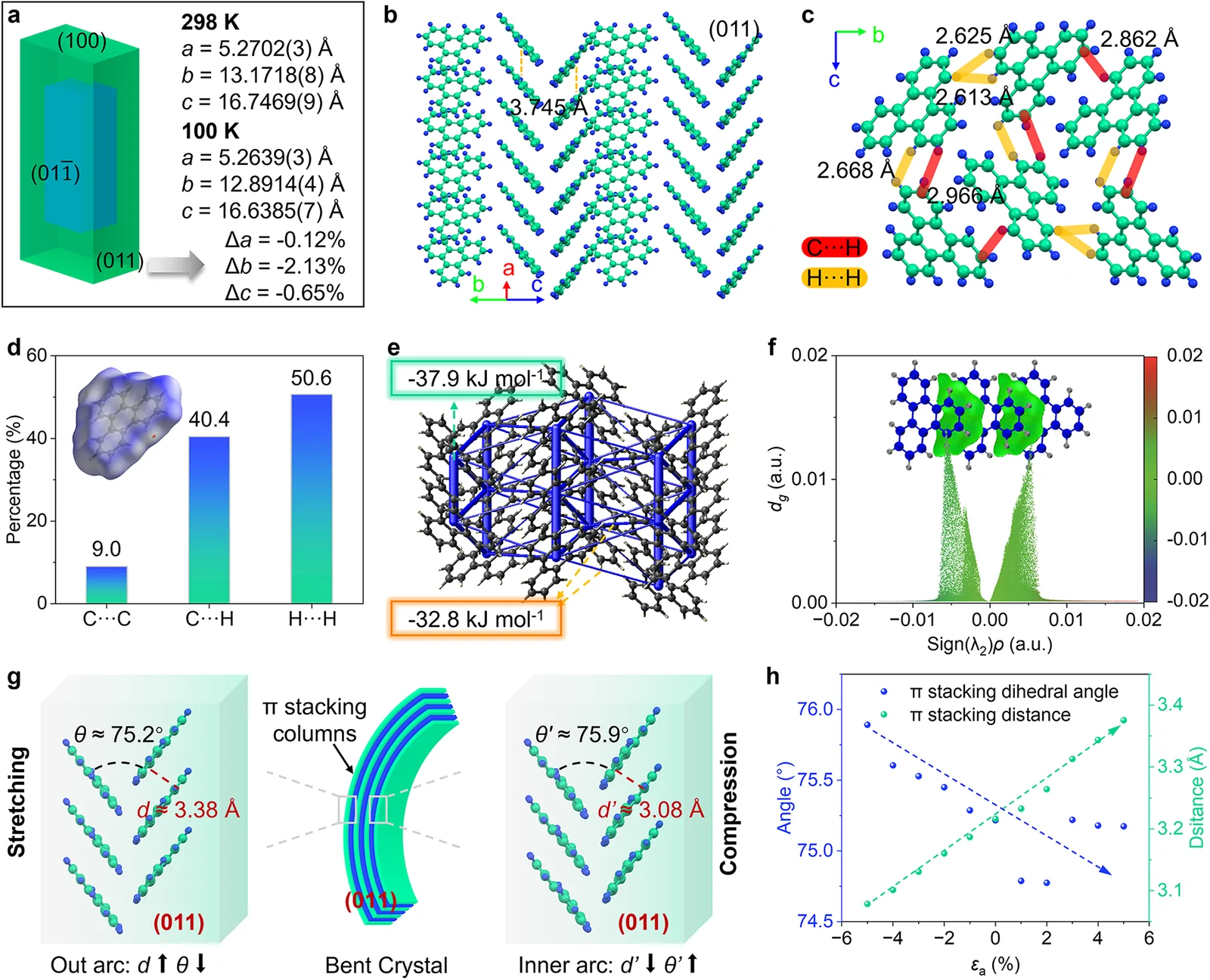
**Mechanistic analysis of the broad-temperature elastic bending in TP_0.9_PER_0.1_** **alloy crystals.** (a) Unit cell parameters at 298 and 100 K. (b, c) Crystal packing arrangement (b) and intermolecular interactions (c) viewed along the (011) plane at 298 K. (d–f) Hirshfeld surface analysis (d), energy framework calculations (e), and IGMH analysis (f) of the dominant noncovalent interactions at 298 K. (g) Schematic illustration of the molecular mechanism for elastic bending. (h) Changes in intermolecular distance and dihedral angle between adjacent TP molecules upon compression and elongation along the *a*-axis.

If viewed from the (011) plane, the molecules are observed to stack along the crystallographic *a*-axis via π···π interactions at a distance of 3.745 Å, forming a columnar structure (Figure 5b). Along the crystallographic *b*-axis, adjacent π-stacking columns are connected by intermolecular hydrogen bonds: C–H···H (2.613 Å, 3.105 Å, 113.6(1)°; 2.625 Å, 3.367 Å, 137.1(4)°) and C–H···C (2.862 Å, 3.629 Å, 140.5(7)°) to form layered plates. In addition, along the crystallographic *c*-axis, neighboring layered plates are further connected by multiple intermolecular hydrogen bonds: C–H···H (2.668 Å, 3.006 Å, 102.2(4)°) and C–H···C (2.966 Å, 3.761 Å, 144.2(9)°) (Figure 5c). In general, the robust π-stacking columns maintain structural integrity, while the abundant weak intermolecular interactions dissipate accumulated stress, collectively enabling elastic deformation.^64,65^

We further performed quantitative analysis of the intermolecular interactions using computational methods. Hirshfeld surface analysis showed that H···H (40.4%) and C···H (50.6%) contacts constitute the largest proportion, indicating that weak interactions are dominant (Figures 5d and Figure S27).^66,67^ This result is consistent with the single-crystal structural analysis (Figure 5c). The intermolecular interaction energies were quantified using the energy framework approach.^68^ The overall distribution of interaction energies is nearly isotropic (Figure 5e). Along the crystallographic *a*-axis, the total interaction energy of the π-stacking columns is the strongest (−37.9 kJ mol^–1^), while the total energies along the *b*- and *c*-axes are −32.8 and −33.6 kJ mol^–1^, respectively (Figures S28 and S29). These abundant intermolecular interactions can dissipate accumulated stress and facilitate structural reconfiguration during deformation. In addition, the independent gradient model based on Hirshfeld partition (IGMH) was employed to analyze the noncovalent interactions along the three crystallographic axes (Figures 5f and Figure S30).^69^ The color-coded sign­(λ_2_)­ρ function mapped onto the isosurfaces reveals that all three molecular pairs are dominated by green regions, corresponding to weak van der Waals interactions (sign­(λ_2_)­ρ = [−0.01, 0.01]). These weak and dispersive interactions are likely involved in the structural adjustments during elastic bending. Furthermore, at low temperature, the crystal undergoes nearly isotropic contraction (Figure 5a), and the weak intermolecular interactions are largely preserved, with energies of −38.6, −35.8, and −36.1 kJ mol^–1^ along the *a*-, *b*-, and *c*-axes, respectively (Figures S31–S33).

Extensive studies using microfocus X-ray diffraction have demonstrated that both the distances and orientations of the molecules during elastic bending of the crystal change, with the intermolecular distances at the outer arc of the bent crystal becoming longer, while those at the inner arc become shorter.^18^ To investigate how TP molecules respond to such changes through specific motions, we performed uniaxial strain-energy calculations.^17^ As shown in Figure 5g, the structural rearrangement could be facilitated by cooperative action between the expansion or contraction of the π-stacking interactions and partial molecular rotation. Specifically, in the outer arc of the crystal, the distance between adjacent TP molecules along the *a*-axis increases and the angle between adjacent TP molecules decreases. In contrast, in the inner arc, the distance decreases and the angle increases. With increasing strain, the overall structure changes in both the inner and outer arcs (Figure 5h). Moreover, abundant weak intermolecular interactions can absorb and dissipate the applied stress during bending, ensuring the reversibility of the structural changes, which may account for the observed elasticity.^18^ Furthermore, owing to the mechanical elasticity of both TP and alloy crystals, OSCHs can undergo elastic deformation via the coordinated deformation of different segments under external force.

### Multichannel Photonic Waveguides, Logic Circuits, and Optical Information Encryption

The efficient emission and cryogenic elasticity of the OSCHs provide a solid foundation for the development of optical logic circuits that are suitable for operation in extreme environments. First, the active optical waveguiding performance of TP and TP_1–*x*_PER_*x*_ alloy crystals was evaluated in both straight and elastically bent states. As shown in Figure 6a and b, a slender TP_0.9_PER_0.1_ single crystal approximately 5 mm in length was selected for testing. The crystal was excited locally with a 365 nm laser beam (spot diameter ∼1 mm) at 1 mm intervals along its long axis, and the outcoupled emission was collected at the left tip. The emission intensity at the tip decreased with increasing propagation distance (Figure 6c). Using a single-exponential fitting function, *I*_tip_/*I*_body_ = *A*exp­(−*αD*), where *I*_tip_/*I*_body_ are the emission intensities at the outcoupling tip and the excitation site, respectively, and *D* is the propagation distance,^28^ the optical loss coefficients (α) were determined to be 0.333 dB mm^–1^ for the straight crystal and 0.357 dB mm^–1^ for the bent crystal (Figure 6d). The same approach was applied to pure TP and other alloy compositions, which showed comparably low optical loss coefficients under both linear and deformed conditions (Figures S34 and S35). These results indicate that elastic bending introduces negligible additional scattering centers or structural defects that would impair light propagation. It has been shown above that the alloy crystals exhibit low optical loss and efficient energy transfer. Moreover, the alloy crystals exhibit excellent spectral reproducibility during repeated cooling–heating cycling, with essentially unchanged emission profiles after 100 cycles (Figure S36). We next examined whether the flexible OSCHs support information transmission along deformable optical pathways at cryogenic temperatures. As illustrated in Figure 6e, five distinct regions were defined on a representative blue–cyan–blue triblock heterostructure and designated as Positions 1–5, serving as input or output channels. Positions 1 and 5 are located in the blue TP segments, Positions 2 and 4 at the blue–cyan interfaces; and Position 3 in the central cyan alloy segment. Upon localized UV laser excitation at Position 1, spatially resolved PL spectra were collected from Positions 1–5 (Figure 6f). The spectra from Positions 1 and 2 were dominated by characteristic peaks in the 380–480 nm range, whereas those from Positions 3–5 showed a clear redshift, with the main emission bands centered in the 480–550 nm region. This redshift results from the substantial spectral overlap between the emission of the TP/alloy segments and the absorption of the central alloy segment. The blue emission generated in the TP domain is reabsorbed by the adjacent alloy block, which then produces its characteristic cyan emission. To further characterize the optical transmission behavior, position-dependent excitation experiments were performed by shifting the excitation spot to Positions 2–5 (Figure 6g–j).

**Figure 6:**
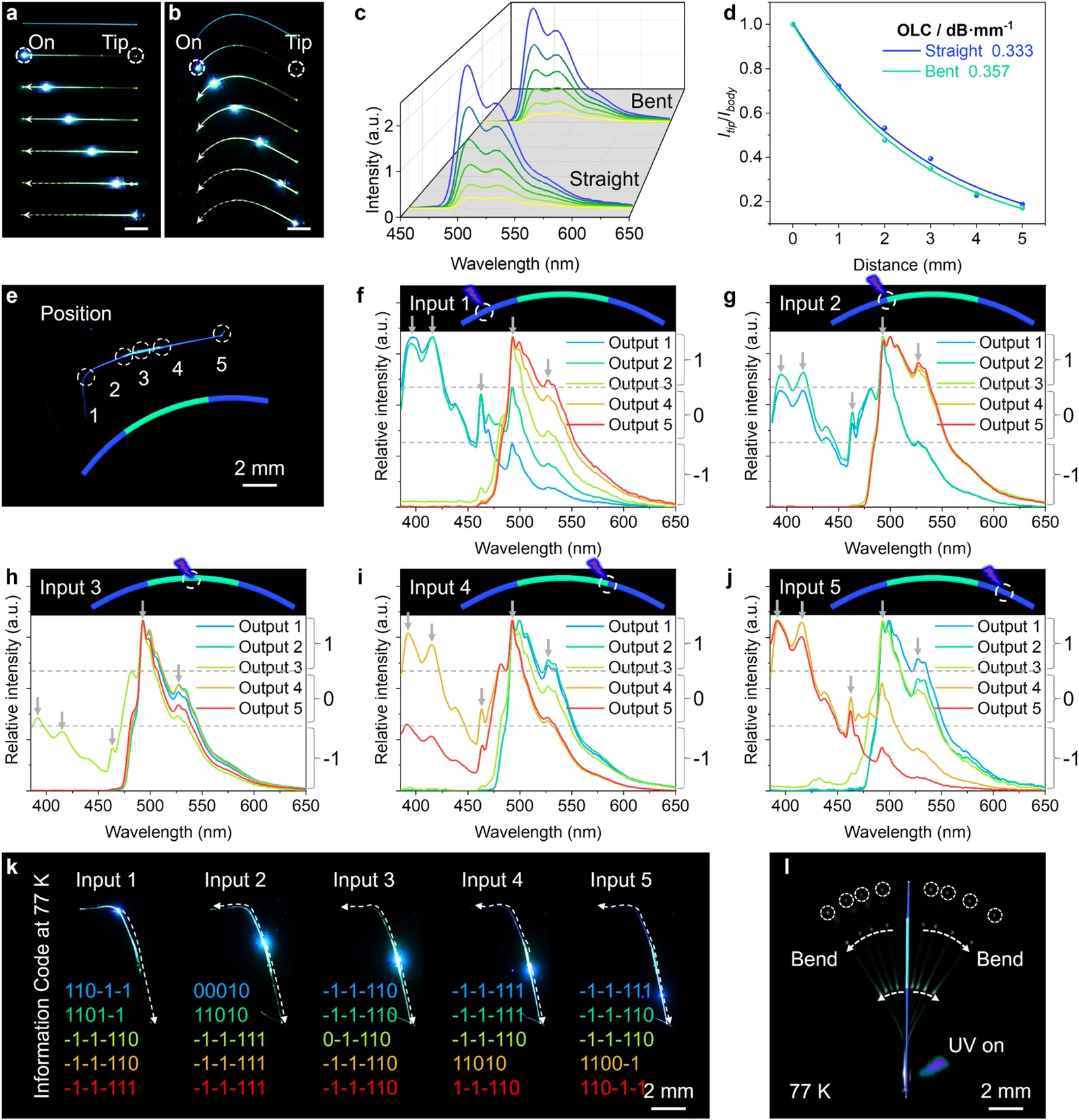
**Active optical waveguides and multichannel optical logic operations based on alloy crystals and OSCHs.** (a, b) Optical photographs of a 5 mm long TP_0.9_PER_0.1_ single crystal operating as an active optical waveguide in the pristine straight state (a) and elastically bent state (b) (scale bars: 1 mm). (c) PL spectra collected at the left tip as a function of excitation position. (d) Spatial decay curves of the outcoupled intensity ratio (*I*_tip_/*I*_body_) for the straight and bent states at room temperature. The optical loss coefficients (*α*) were obtained by single-exponential fitting to the equation *I*_tip_/*I*_body_ = *A*exp­(−*αD*), where *D* is the propagation distance. (e) Schematic showing a triblock heterostructure divided into five operational regions (Positions 1–5). (f–j) Spatially resolved PL spectra recorded at 77 K from the output channels upon localized excitation at Positions 1–5, respectively (insets show the excitation positions). (k) Digitized ternary optical logic signals (“1”, “0”, “–1”) extracted from the five characteristic emission wavelengths (395, 415, 462, 493, and 526 nm). (l) Dynamic fluorescent signal transmission through a mechanically bent OSCH (dashed circles highlight the outcoupling ends at different bending angles).

The relative intensities of selected PL peaks were classified into three logic states (“1”, “0”, and “–1”) (Figure 6f–j). Five representative emission wavelengths (395, 415, 462, 493, and 526 nm) were extracted, and the corresponding logic output matrices were determined for each excitation position. For excitation at Position 1, the output signals across Positions 1–5 were as follows: Position 1: “110–1–1”; Position 2: “1101–1”; Position 3: “–1–1–110”; Position 4: “–1–1–110”; Position 5: “–1–1–111” (Figure 6k). Building on these results, the dynamic waveguiding behavior of diblock and triblock OSCHs was investigated under cryogenic conditions. By maintaining a fixed excitation position and adjusting the orientation of the out-coupling ends through controlled bending, multidirectional and multichannel optical signal transmission was achieved (Figure 6l and Figure S37). These experiments demonstrate that external spatial control of the optical logic outputs can be realized by simple mechanical bending of the heterostructure.

The tunable block-length ratios of the multicolored segments in the triblock OSCHs enable their use as physical-layer photonic barcode systems for information encryption (Figure 7a). Three discrete regions across a single heterostructure were defined as Positions 1–3, with Positions 1 and 3 located in the terminal blue domains and Position 2 in the central cyan domain, serving as input/output channels for optical signal transduction. As shown in Figure 7b, laser excitation at Position 1 produces outcoupled PL spectra that are collected at Positions 1, 2, and 3. These spectra are converted into optical barcodes using a standardized encoding rule: the wavelengths of the characteristic emission peaks determine the positions of the barcode lines, while the relative intensities of those peaks determine the line widths. Specifically, the spectrum recorded at Position 1 (peaks at 410, 460, and 490 nm; Figure 7b) is translated into the blue segments of Barcode 1 (Figure 7a), with the gradual decrease in peak intensity resulting in progressively narrower lines. The spectrum at Position 2 (peaks at 476, 494, and 527 nm) generates the green segments of Barcode 1, and the spectrum at Position 3 (peaks at 501 and 527 nm) generates the red segments of Barcode 1. Different excitation modes (Figure 7c and d) produce distinct barcode patterns (Barcode 2 and Barcode 3) through the same process. The complete barcode is then formed by joining these three color-coded segments in sequence (Figure 7a).

**Figure 7:**
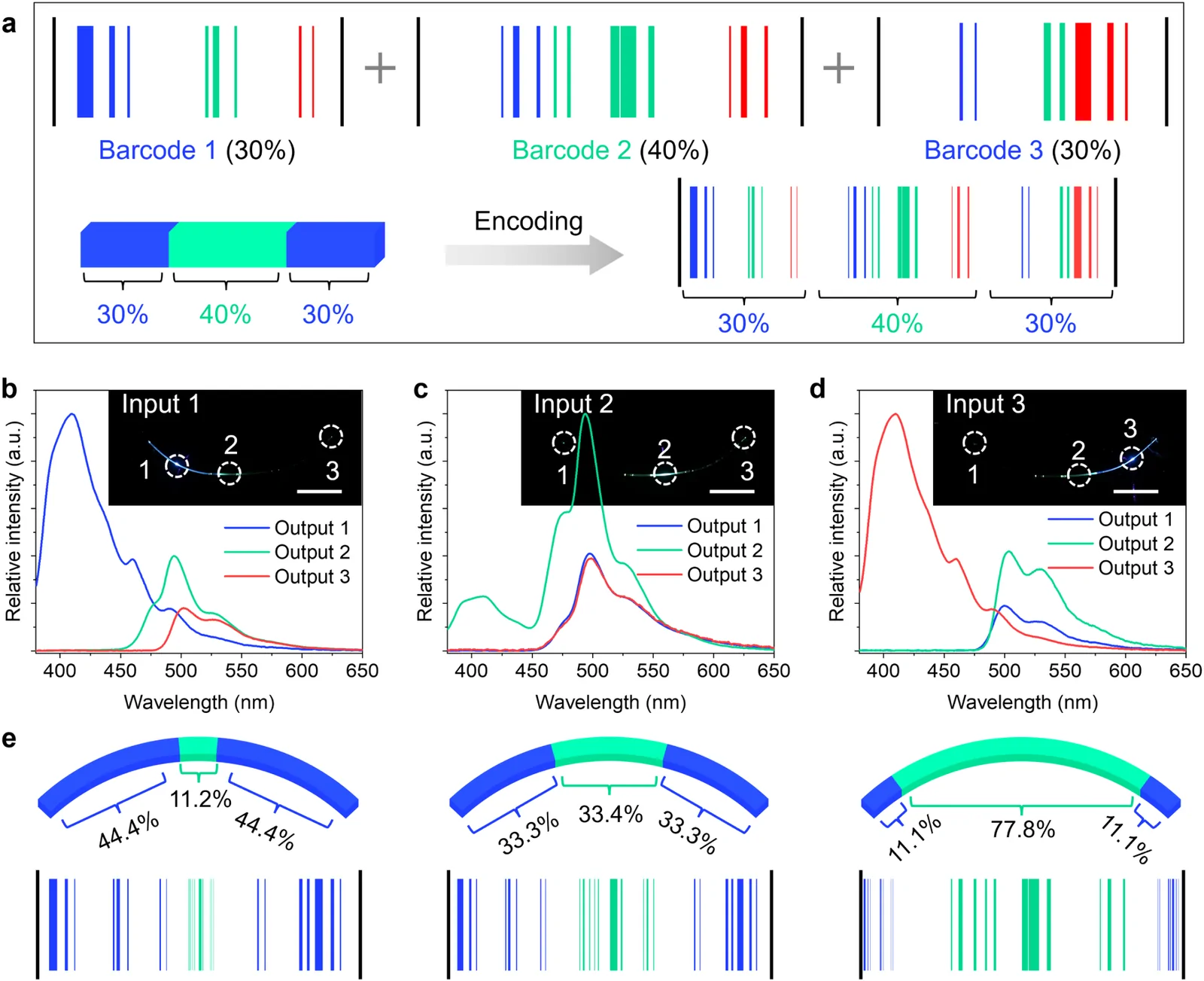
**Optical information encryption and programmable photonic barcodes based on triblock OSCHs.** (a) Schematic illustration of the encoding rules for generating digitized barcodes from the heterostructure emission profiles (black lines indicate division lines; blue, green, and red lines represent barcode segments). (b–d) PL spectra collected from the input and output positions under Excitation Mode 1 (b), Mode 2 (c), and Mode 3 (d) at room temperature (insets show the corresponding fluorescence micrographs of the triblock OSCH, with outcoupling ports highlighted by dashed circles; scale bars: 2 mm). (e) Customized barcode patterns generated from OSCHs with different block length ratios obtained by mechanical sectioning.

Furthermore, the widths of the individual barcode segments are directly proportional to the physical lengths of the corresponding crystalline blocks. For example, a blue (11.1%)–cyan (77.8%)–blue (11.1%) heterostructure produces shorter barcode segments 1 and 3, but a longer barcode segment 2, compared with a blue (44.4%)–cyan (11.2%)–blue (44.4%) heterostructure (Figure 7e). As a result, the encoded patterns can be readily customized by mechanically sectioning the flexible blue or cyan blocks to adjust their relative lengths (Figure 7e). Finally, these heterostructures maintain reliable signal transmission under elastic bending, including at low temperatures. The resulting multicolored barcodes are easy to read yet difficult to duplicate, providing a practical platform for flexible optical anticounterfeiting labels.

## Conclusions

In summary, we have fabricated cryogenically flexible blue–cyan–blue triblock organic single-crystal heterostructures (OSCHs) via an organic alloy epitaxial growth strategy. Near-perfect lattice matching between the TP host and PER-doped alloy crystals enables the precise construction of well-defined heterostructures with spatially resolved emission. Efficient Förster resonance energy transfer (FRET) and the high photoluminescence quantum yield of TP endow the alloy segments with a prominent PLQY of 0.659, supporting low-loss active optical waveguiding. Comprehensive structural analyses, including single-crystal X-ray diffraction, Hirshfeld surface analysis, energy framework analysis, IGMH analysis, and strain-energy calculations, reveal that the flexibility originates from abundant weak intermolecular interactions (primarily C–H···H/C and π···π stacking) along with cooperative molecular sliding and rotation under uniaxial strain, which persist at both 298 and 100 K. Consequently, the resulting OSCHs maintain excellent elastic deformability and multichannel optical signal transmission under cryogenic conditions, enabling applications in optical logic gates, dynamic waveguides, and tunable photonic barcodes for information encryption. This molecular alloying strategy provides a powerful molecular-level approach for integrating mechanical robustness with sophisticated photonic functions in single-crystal heterostructures, opening new opportunities for flexible optoelectronic devices that can operate in extreme environments.

## Supplementary Material













## Data Availability

Crystal data of TP_0.9_PER_0.1_ alloy are available from the Cambridge Crystallographic Data Centre under reference numbers 2552119 and 2552120. All data are available from the corresponding authors upon request.

## Notes

Elastic bending of TP_0.9_PER_0.1_ crystal at room temperature (MP4)
Elastic bending of TP_0.95_PER_0.05_, TP_0.98_PER_0.02_, TP_0.99_PER_0.01_, TP_0.995_PER_0.005_ crystals at room temperature (MP4)
Elastic bending of TP_0.9_PER_0.1_, TP_0.95_PER_0.05_ crystals at liquid nitrogen temperature (MP4)
Elastic bending of diblock and triblock single-crystal heterostructures at room temperature (MP4)
Elastic bending of diblock and triblock single-crystal heterostructures at liquid nitrogen temperature (MP4)
Experimental procedures; preparation of crystals; optical and physical properties of crystals; chemical structures and approximate sizes of molecules; characterization of crystal phases; bending of the crystals; characterization of the mechanical properties of crystals; cyclability testing; photoluminescence and mechanical properties of multiblock crystals; crystal structure and molecular packing; face indexing of an alloy crystal based on X-ray diffraction analysis; analysis of intermolecular interactions based on IGMH; energy framework calculations; optical waveguiding characterization; crystal data (PDF)
