# Supplementary material for: Cryogenically Flexible Organic Single-Crystal Heterostructures via Molecular Alloying for Advanced Optical Tuning
Source: J Am Chem Soc. 2026 Oct 27;148(44):48128–40. doi: 10.1021/jacs.6c14819 (PMC13761272; doi:10.1021/jacs.6c14819)
Supplement: Supplementary file 1 [file ja6c14819_si_006.pdf]

Supporting Information

Cryogenically Flexible Organic Single-Crystal Heterostructures via Molecular Alloying for Advanced Optical Tuning

Wenbo Wu,[1¶] Yifang Lu,[1¶] Mingliang Wu,[2] Jiaxuan Zhu,[1] Hui Yu,[1] Xin Huang,[1,3] Na Wang,[1,3]* Qiang Li,[4] Ting Wang,[1,3]* Dongyang Zhu,[1,3]* Panče Naumov,[5,6,7,8]* Hongxun Hao[1,3]

[1]*National Engineering Research Center of Industrial Crystallization Technology, School of Chemical Engineering and Technology, Tianjin University, Tianjin 300072, P. R. China*
[2]*Suzhou Laboratory, Suzhou, Jiangsu, 215123, P. R. China*
[3]*Engineering Research Center of Green Refining Process, Ministry of Education, Tianjin University, Tianjin, 300072, P. R. China*
[4]*Key Laboratory of Quantum Materials and Devices of Ministry of Education, School of Physics, Southeast University, Nanjing, 21189, P. R. China*
[5]*Smart Materials Lab, New York University Abu Dhabi, 129188, Abu Dhabi, UAE*
[6]*Center for Smart Engineering Materials, New York University Abu Dhabi, 129188, Abu Dhabi, UAE*
[7]*Research Center for Environment and Materials, Macedonian Academy of Sciences and Arts, Bul. Krste Misirkov 2, MK–1000 Skopje, Macedonia*
[8]*Molecular Design Institute, Department of Chemistry, New York University, 100 Washington Square East, New York, NY 10003, USA*
Corresponding authors. Emails: wangna224@tju.edu.cn (N. Wang); wang_ting@tju.edu.cn (T. Wang); zhudongyang@tju.edu.cn (D. Zhu); pance.naumov@nyu.edu (Prof. P. Naumov)
[¶]These authors contributed equally to this work.

1. Materials and Methods

Materials. Triphenylene (TP, CAS: 217-59-4) and perylene (PER, CAS: 198-55-0) were purchased from HEOWNS, acetonitrile (analysis grade), dichloromethane (analysis grade), *n*-hexane (analysis grade), acetone (analysis grade), ethyl acetate (analysis grade), and ethanol (analysis grade) were purchased from BOHUA CHEMICAL. All compounds and solvents were used without further treatment.

Powder X–ray diffraction. The powder X–ray diffraction data were obtained on a Rigaku D/max–2500 (Rigaku, Japan) using CuK_α radiation (1.5405 Å), with a step size of 0.02° and a scanning rate of 0.067° s^{-1} over diffraction angle (2θ) range of 5–35°.

Thermal analysis. The DSC measurement was performed on a calorimeter model DSC 1 (Mettler Toledo, Switzerland) calibrated with standard indium and zinc under a nitrogen atmosphere to determine the thermodynamic properties of the samples. Samples weighing 5–10 mg were placed in standard aluminum pans and heated at a heating rate of 10 K min^{-1} under a nitrogen gas flow of 50 mL min^{-1}.

Single–crystal X–ray diffraction. The single crystal X–ray diffraction (SCXRD) measurements were carried out on 007HF XtaLAB P20 (Rigaku, Japan) diffractometer. The data was collected at the temperatures of 100 K and 298 K, while the temperature was controlled with an Oxford Cryostream 800 cooling system.[1] Olex2 was used to solve the structure by intrinsic phasing methods (SHELXT), and the full–matrix least–squares method on F^2 (SHELXL) was used to complete and refine the structure models.[2]

Fluorescence spectroscopy. Fluorescence spectra and lifetimes were recorded by using a photoluminescence spectrometer (Edinburgh Instruments, FLS1000, UK). The spatially-resolved photoluminescence spectra and optical waveguides were tested using the AvaSpec ULS2048XL-EVO.

Ultraviolet–visible absorption spectroscopy. Ultraviolet–visible absorption spectra were recorded by using an ultraviolet visible near–infrared spectrophotometer (Perkin Elmer, LAMBDA 750, China).

Scanning electron microscopy. The morphology of the crystals was observed by using an X650 scanning electron microscope (Hitachi, Japan). The samples were mounted by using a carbon adhesive on an aluminum holder, sputtered with gold, and scanned at a voltage of 15 kV under vacuum.

Nanoindentation. The nanoindentation tests were conducted by using nanomechanical test instrument TI Premier (Hysitron Inc.) with a Berkovich diamond tip indenting the main crystal facets. All crystals were firmly affixed on the test stage with a cyanoacrylate glue. Peak load P_{max} = 2 mN at loading/unloading rate of 0.5 mN s^{-1} and hold time of 10 s was applied in all cases. A minimum of 10 indentations were carried on each crystal facet to obtain satisfactory statistics. The *P*–*h* response curves were analyzed by using the standard Oliver–Pharr method.[3]

Hirshfeld surface and energy framework analysis. The Hirshfeld surface 2D and energy framework analysis were performed and the results were plotted by using the CrystalExplorer software (ver. 17.5).[4] The energy frameworks were calculated at B3LYP/6-31G(d,p) level of dispersion-corrected density functional theory basis set.[5] The energy components calculated within this method are electrostatic, polarization, dispersion, and exchange-repulsion and finally the total interaction energy. They were calculated by adding corresponding interaction energies between a given molecule in one layer and all interacting molecules in a neighboring layer or within the same layer within 3.8 Å. The tube size used in the energy framework was 100 and lower energy threshold (cut-off) value was set to -2 kJ mol^{-1}.

Independent gradient model based on Hirshfeld partition (IGMH) analysis. The popular B3LYP exchange-correlation functional in combination with a medium-sized 6-311G** basis set was used for geometry optimization and generation of the wavefunction for IGMH analysis.[6] The Gaussian 16 A.03 program was used for all the density functional theory (DFT) calculations.[7] All isosurface maps were rendered by VMD 1.9.3 program based on the cube files generated by Multiwfn. [8] Grid spacing of 0.15 Bohr was employed in calculating the grid data involved in IGMH analyses.

Calculation of the strain-energy. First-principles calculations were performed using the Vienna Ab initio Simulation Package, VASP 5.4.4.[9] The projector augmented wave method was employed to describe the electron–ion interactions, and the exchange-correlation energy was treated using the generalized gradient approximation with the Perdew-Burke-Ernzerhof functional.[10,11] Long-range dispersion interactions, which are essential for describing the weak intermolecular contacts in molecular crystals, were included using the Grimme DFT-D3 method.[12] The plane-wave kinetic energy cutoff was set to 500 eV. Electronic self-consistency was achieved with an energy convergence criterion of 1.0×10^{-6} eV. Structural relaxations were carried out using the conjugate-gradient algorithm with a force convergence criterion of 0.02 eV $Å^{-1}$. During the strained-structure optimizations, the lattice parameters were kept fixed and only the atomic positions were relaxed. To simulate uniaxial deformation, the optimized 298 K single-crystal structure was used as the reference configuration. A series of strained structures were generated by systematically compressing and elongating the crystallographic *a*-axis from -5% to +5%, while keeping the fractional atomic coordinates initially unchanged. For each strained structure, the deformed lattice was fixed and all atomic positions were fully relaxed.

2. Experimental section

Fabrication of TP single crystal. At 318.15 K, the TP was dissolved in the acetonitrile solvent to obtain a saturated solution. This solution was then filtered and cooled to 273.15 K at a cooling rate of 5 K h^{-1}. After 2 hours of crystal growth at 273.15 K, TP single crystals were obtained.

Fabrication of alloy single crystals. At 318.15 K, measured molar proportion of the mixture of TP and PER was dissolved in the acetonitrile solvent to obtain a saturated solution. After filtration, it was

cooled to 273.15 K at a cooling rate of 5 K h^{-1}. After 2 hours of crystal growth at 273.15 K, single crystals of the molecular alloy (mixed crystals) were obtained.

Fabrication of the single–crystal heterostructures. First, crystals of $TP_{0.9}PER_{0.1}$ were prepared by the above method. Then, a supersaturated pure TP solution was prepared by the following protocol: at 308.15 K, 198 mg of TP was dissolved in 30 g of acetonitrile. After filtration, the temperature was lowered to 303.15 K and maintained for 2 hours at a constant temperature. Finally, the $TP_{0.9}PER_{0.1}$ crystals were added to the above TP solution, and then cooled at a rate of 10 K h^{-1} to 273.15 K.

The solvents used included acetone, 1,4–dioxane, dichloromethane, and chloroform. In all cases, blue–cyan–blue triblock OSCHs were obtained. Generally, the solvent could affect the dimensions of OSCHs, with the most pronounced influence on crystal thickness. Furthermore, we varied the initial solution temperature (293.15–323.15 K) and cooling rate (2–10 K h^{-1}) to identify the optimal OSCHs growth conditions. When crystallization was initiated from a higher initial temperature (e.g., 323.15 K), the emission color between the primary and newly grown blocks became similar. This was mainly reflected by an increase in the emission peak at 410 nm of the cyan block (Figure S19). We hypothesize that the alloy crystals will undergo the process of dissolution and recrystallization, when introduced into the saturated acetonitrile solution of TP (we note that even in a saturated solution, dissolution persists because dissolution and recrystallization are in a dynamic equilibrium). High temperatures accelerate both dissolution and recrystallization rates, leading to reduce PER content in the primary blocks and increase PER content in the newly grown blocks. As demonstrated earlier, a slight change in PER content (as little as 0.1%) can dramatically alter the optical properties (Figure 2f). In addition, under a slow cooling rate (e.g., 10 K h^{-1}), it also reduced the emission color contrast between the heterostructure blocks. This behavior is likely attributed to prolonged crystallization time, which also reduced PER content in the newly grown blocks. Finally, we attempted to prepare cyan–blue–cyan triblock OSCHs by adding pure TP crystals into a TP/PER mixed solution. However, as the primary TP crystals incorporated PER molecules, yielding heterostructures with virtually no discernible difference in emission color.

Low temperature test. The crystals were cooled by direct rapid immersion in liquid nitrogen rather than by a controlled cold-gas stream. Specifically, a cylindrical iron block was placed inside a liquid-nitrogen Dewar and used as a precooled stage. Liquid nitrogen was added and maintained for approximately 4 h, with periodic refilling to keep the liquid level above the upper surface of the iron block. Individual crystals were then rapidly immersed in liquid nitrogen and subsequently transferred onto the precooled iron platform for mechanical testing under cryogenic conditions.

Optical waveguide test. The active optical waveguiding properties were measured using a home-built spatially resolved photoluminescence setup. AvaSpec ULS2048XL-EVO was used to collect the photoluminescence spectra. A 365 nm continuous-wave laser was used as the excitation source. The laser power density at the sample was 10 mW·cm^{-2}, with a spot diameter of ~1 mm. The laser was installed on a manually driven XYZ moving platform. The crystal was mounted on the fixed optical platform. For straight-state measurements, a single crystal with a length of ~5 mm, width of ~30 μm,

and thickness of ~20 μm was used. For bent-state measurements, the same crystal was manually bent and then fixed in its new shape. After bending, the left tip was re-aligned to the collection optics to ensure identical collection geometry.

Specifically, the emission from the left end of the crystal was collected with an optical fiber and recorded using a spectrometer (AvaSpec ULS2048XL-EVO). The translation stage was then moved to shift the excitation spot to the right along the crystal long axis in 1 mm increments. At each position, a spectrum was recorded, and the emission intensity was denoted I_{tip}, with the first data set denoted I_{body}. The propagation distance D was defined as the distance from the excitation spot to the left tip along the crystal long axis. For a bent crystal, D was taken as the arc length along the crystal centerline. The intensity ratio was fitted with $I_{tip}/I_{body} = A\exp(-\alpha D)$, where α is the optical loss coefficient in dB mm^{-1}.

3. Supplementary Figures

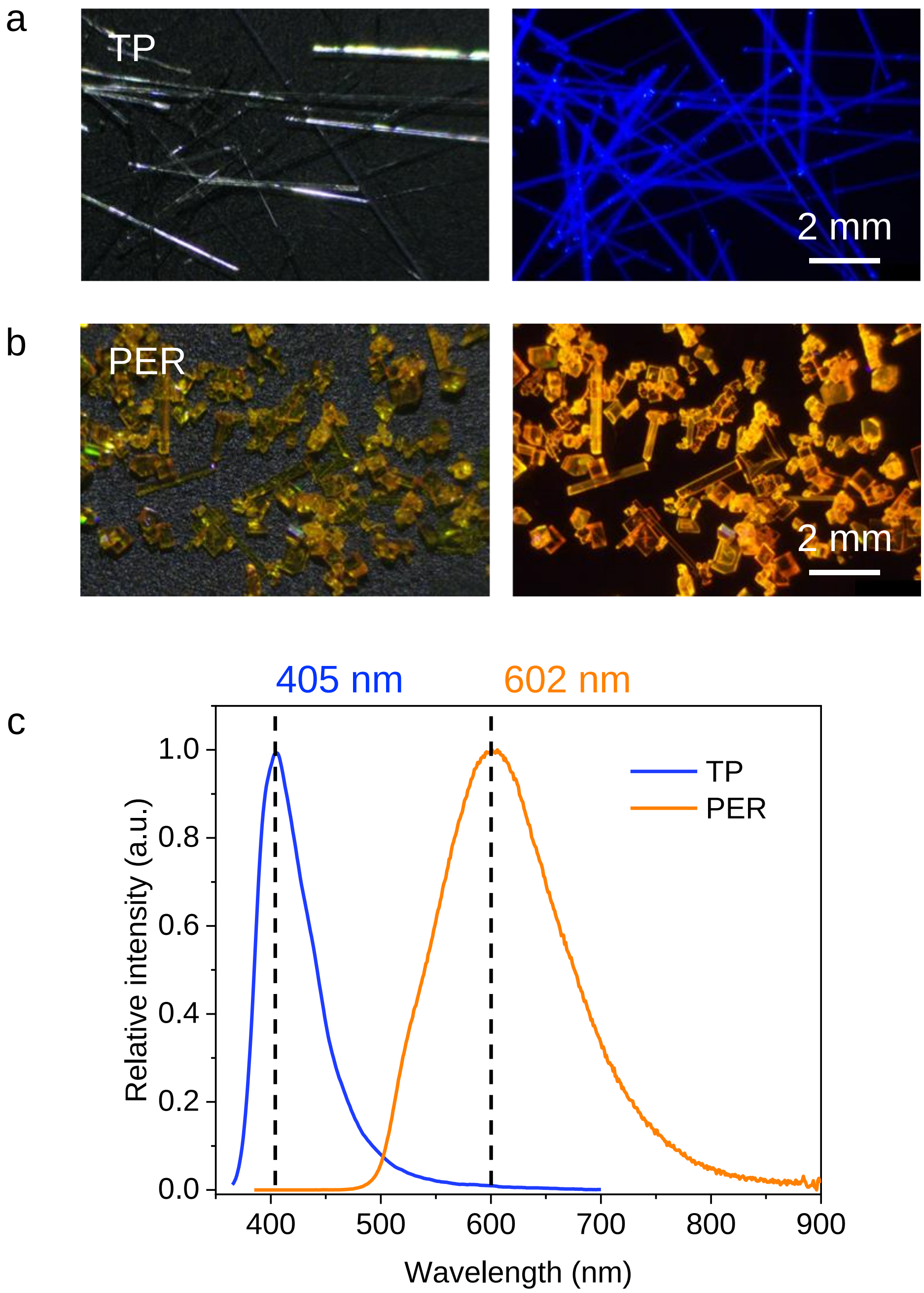

Figure S1. (a, b) Photographs of the TP (a) and PER (b) single crystals under daylight and under UV radiation (365 nm). (c) Photoluminescence spectra of the TP (blue line) and PER (orange line) crystals.

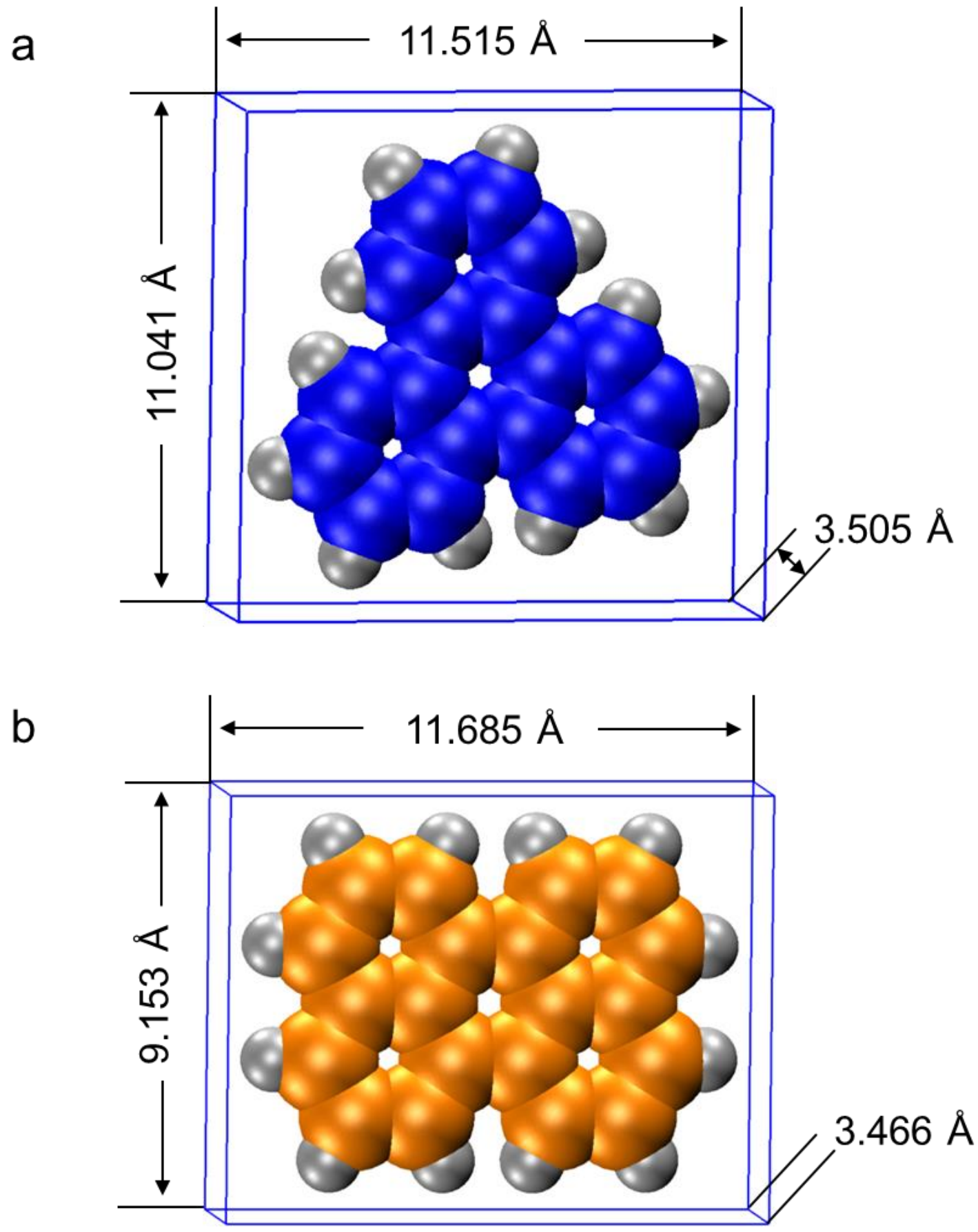

Figure S2. Chemical structures and approximate size of TP (a) and PER (b) molecules defined by van der Waals surfaces, including the hydrogen atoms. The molecular length, width, and height were evaluated using the Multiwfn program. [8] The procedure constructs the van der Waals (vdW) surface of the molecule by assigning the UFF atomic radii to each nucleus. A systematic rotational search over Euler angles is then performed to find the molecular orientation that gives the smallest axis-aligned rectangular box that completely encloses this vdW surface. The three orthogonal box edges after this volume-minimization step are sorted as length (largest), width (intermediate), and height (smallest), providing an orientation-independent measure of molecular size. The equilibrium geometry was obtained from full optimization at the M06-2X/def2-TZVP level, and the size analysis employed the default settings of Multiwfn.

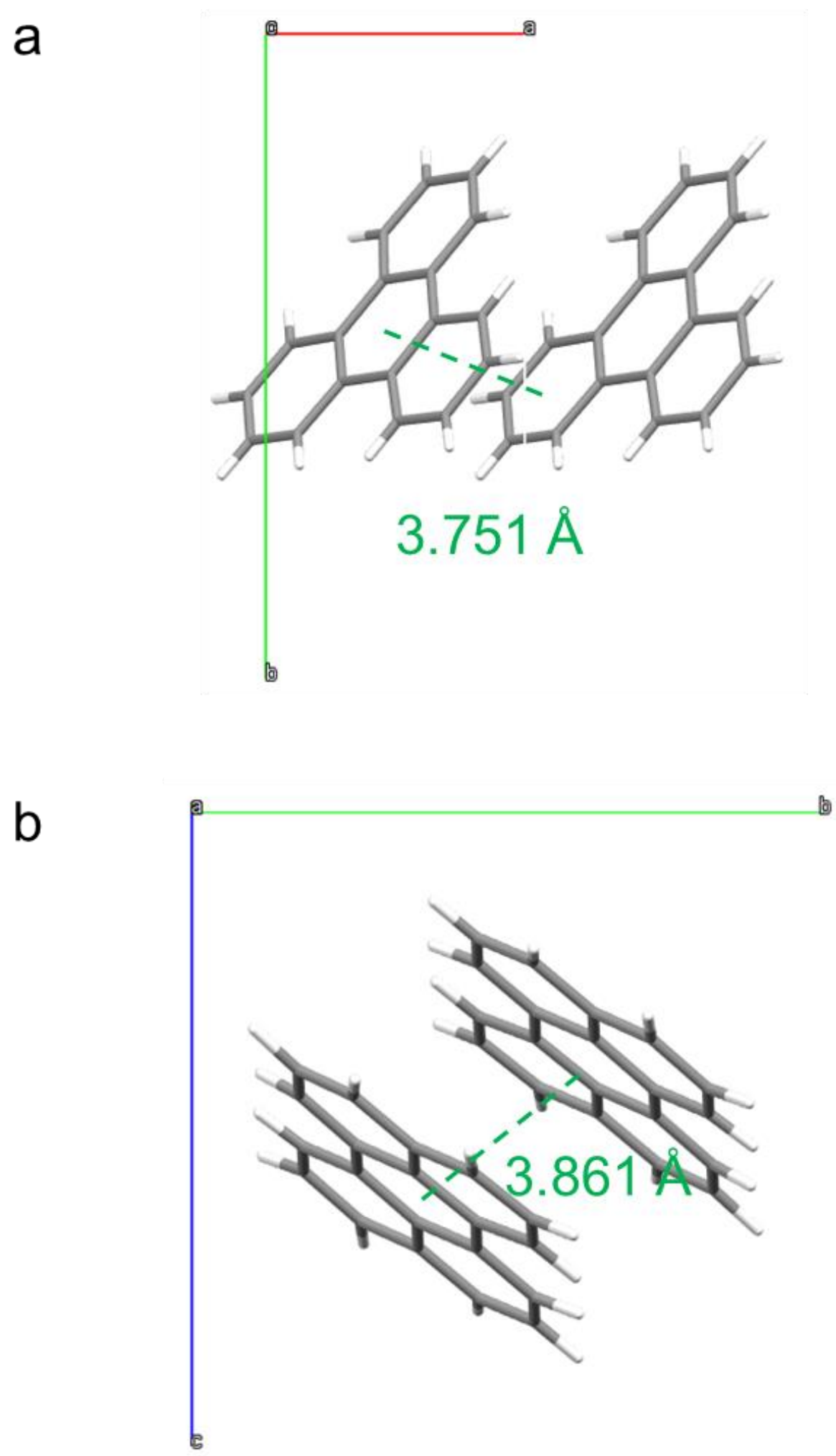

Figure S3. Intermolecular π-stacking distances in the crystal structure of TP (a) and PER (b).

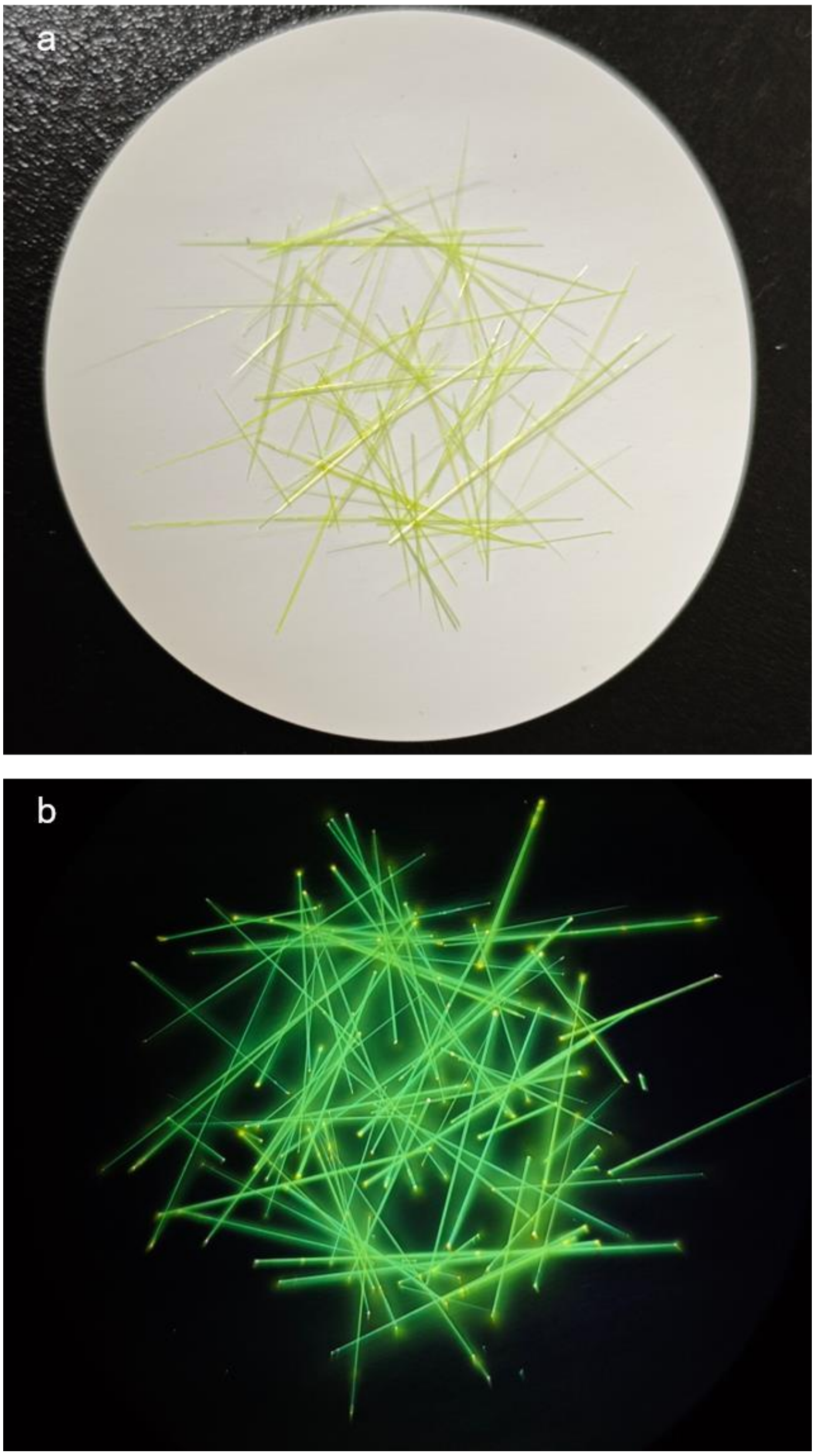

Figure S4. Photographs of alloy single crystal under daylight (a) and under UV radiation (b).

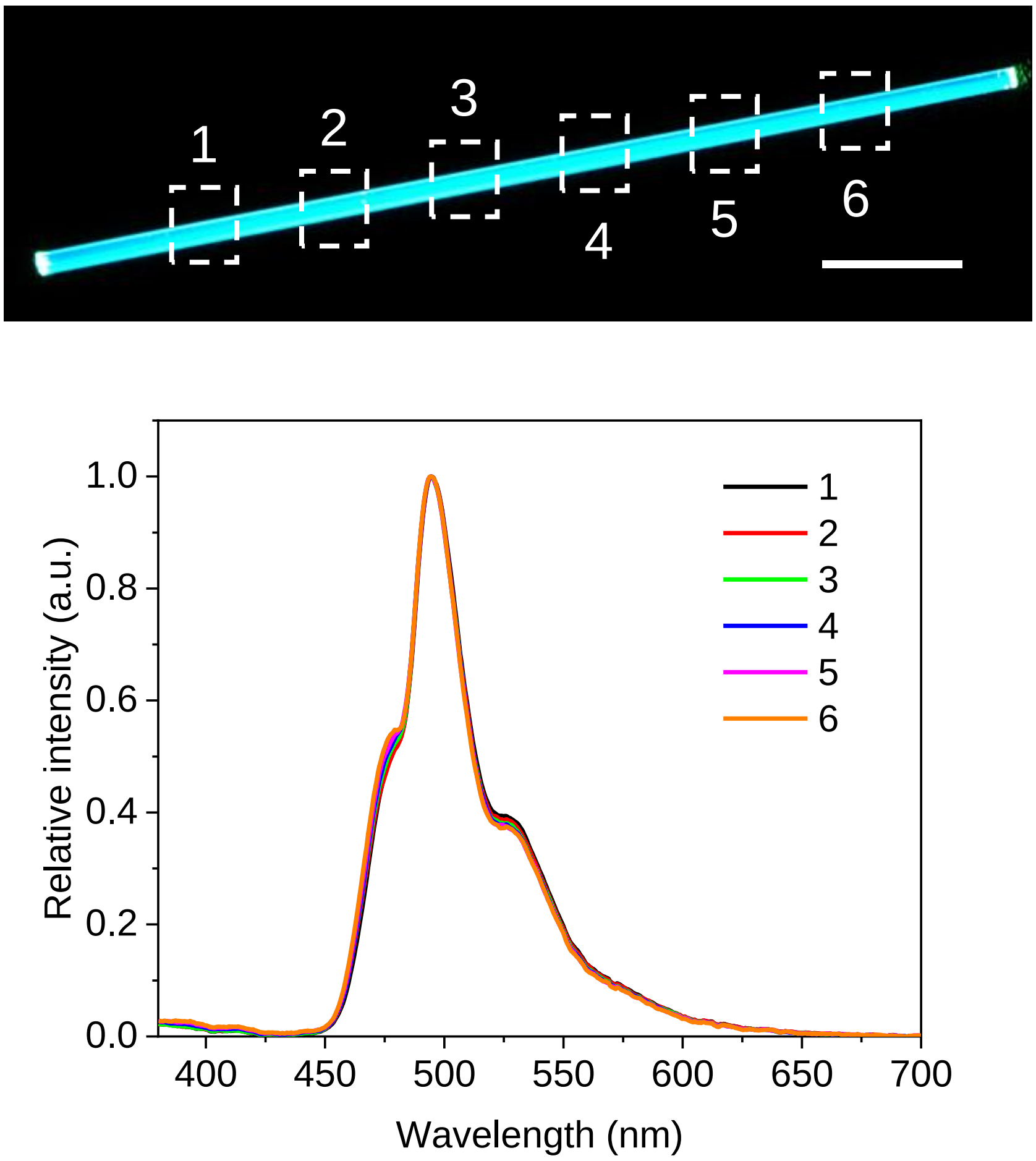

Figure S5. Optical characterization of the $TP_{0.9}PER_{0.1}$ alloy crystal. (a) Fluorescence images. Scale bar 1 mm. (b) Photoluminescence spectra collected from different sections marked in fluorescence images of $TP_{0.9}PER_{0.1}$ alloy crystal excited by UV light (λ = 365 nm). Spatially resolved photoluminescence spectroscopy confirms the compositional uniformity of the alloy, as evidenced by the nearly identical photoluminescence spectra recorded at different positions.

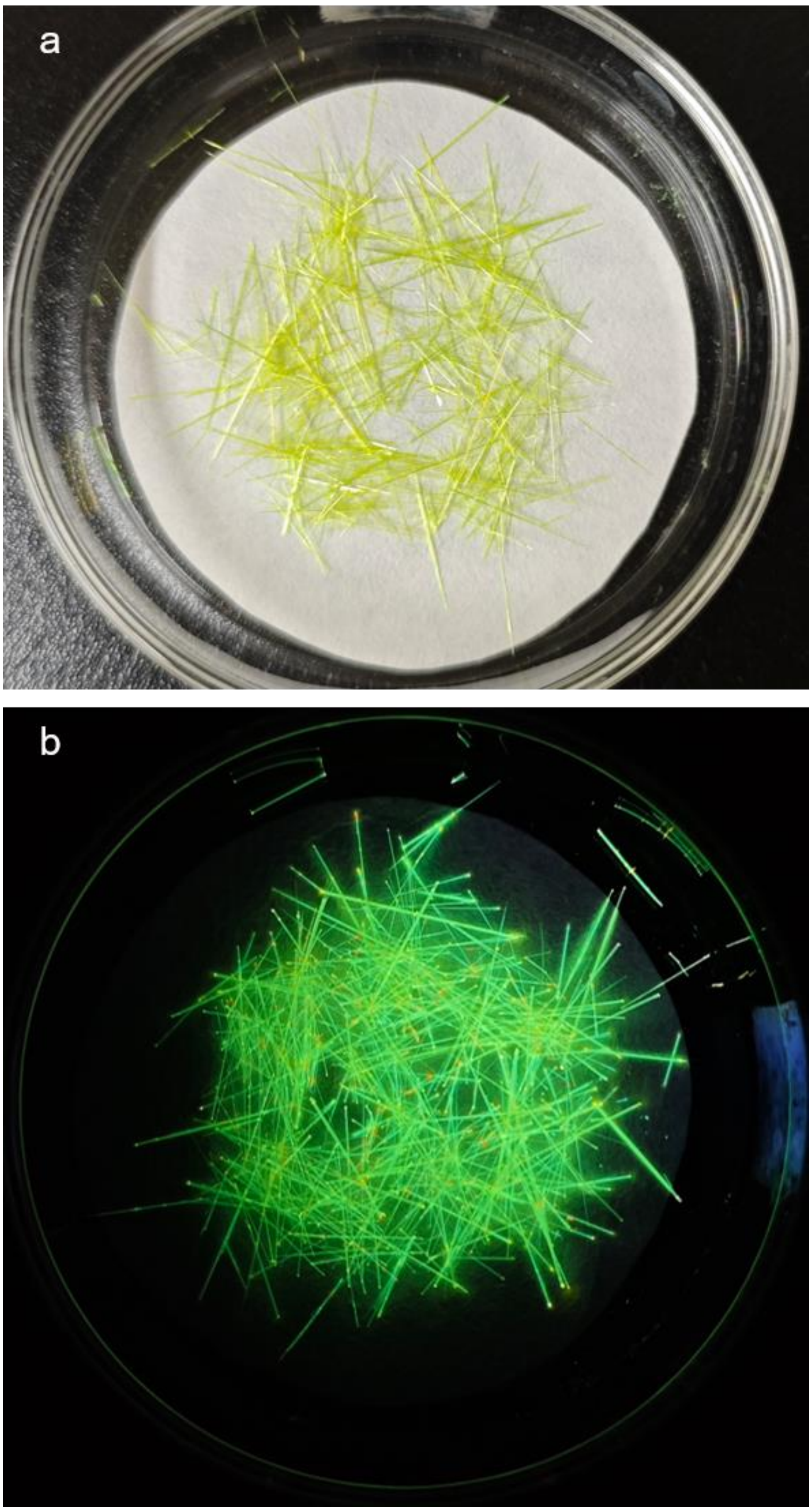

Figure S6. Photographs of mixed single crystal under daylight (a) and under UV radiation (b).

a

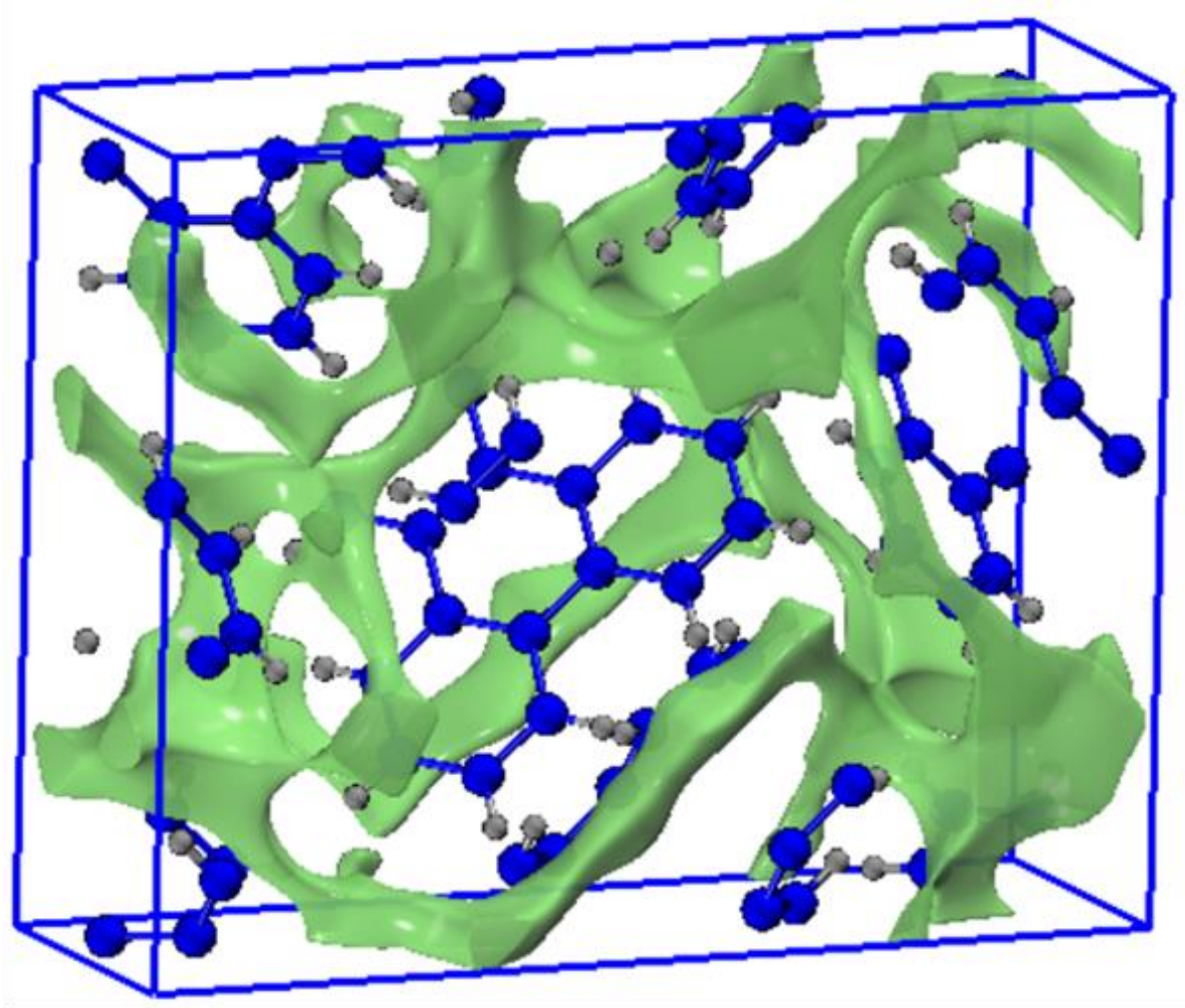

29.6%

b

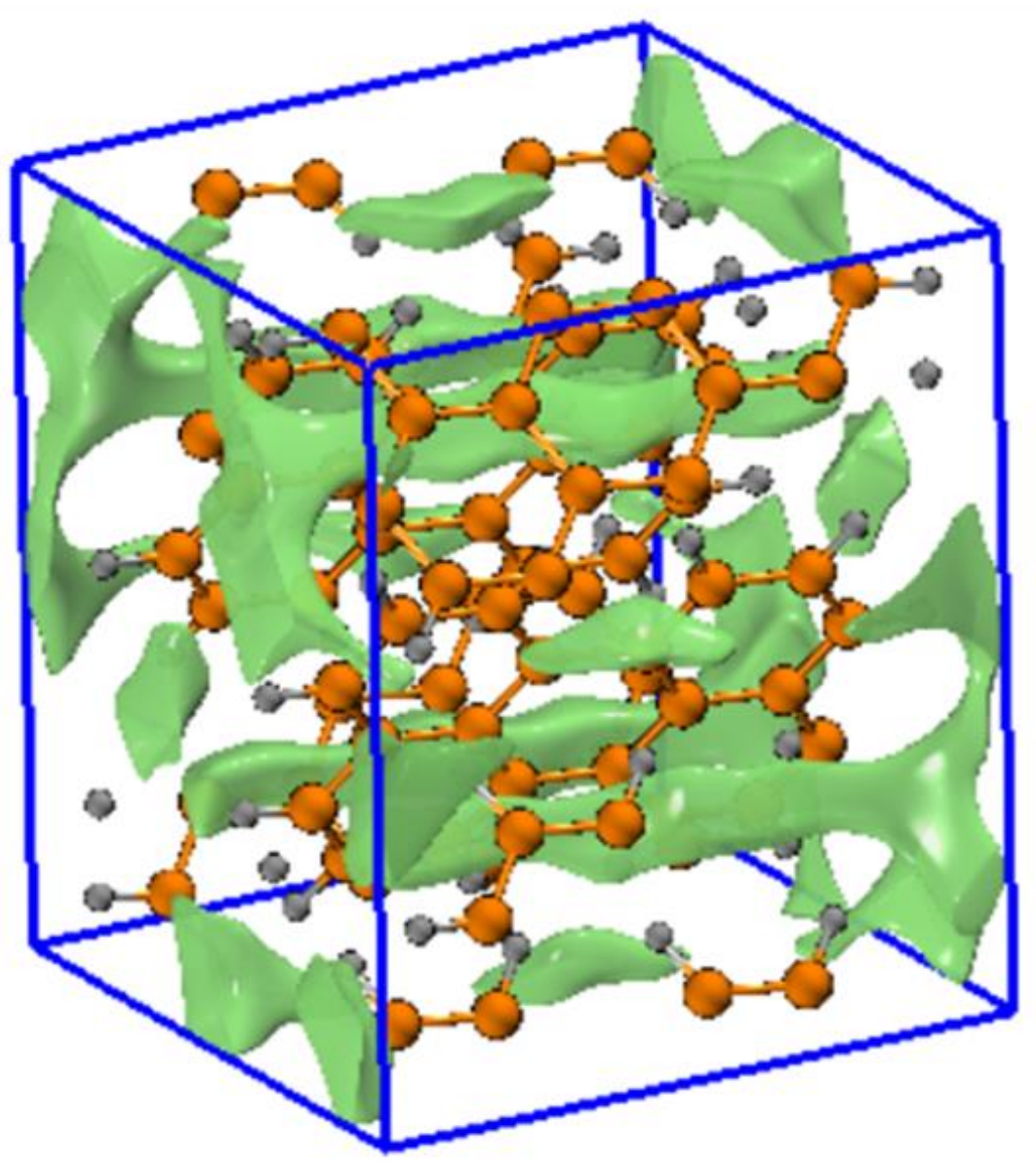

25.1%

Figure S7. Calculated fractional free volumed of TP (a) and PER (b). The free volumes (voids) in the crystal lattice were identified and visualized using Multiwfn program.[8] The unit cell was partitioned into a three-dimensional grid with a spacing of 0.1 Å. Void regions were defined as grid points outside the van der Waals (vdW) spheres of all atoms (employing Bondi's vdW radii) and were filtered by a probe sphere of radius 1.2 Å to locate accessible voids. Connected void domains were clustered, and the total void volume and its fraction were computed. The void occupancy map was exported as a Gaussian cube file and rendered as an isosurface (isovalue = 1) superimposed on the crystal packing to graphically represent the free-volume distribution.[13]

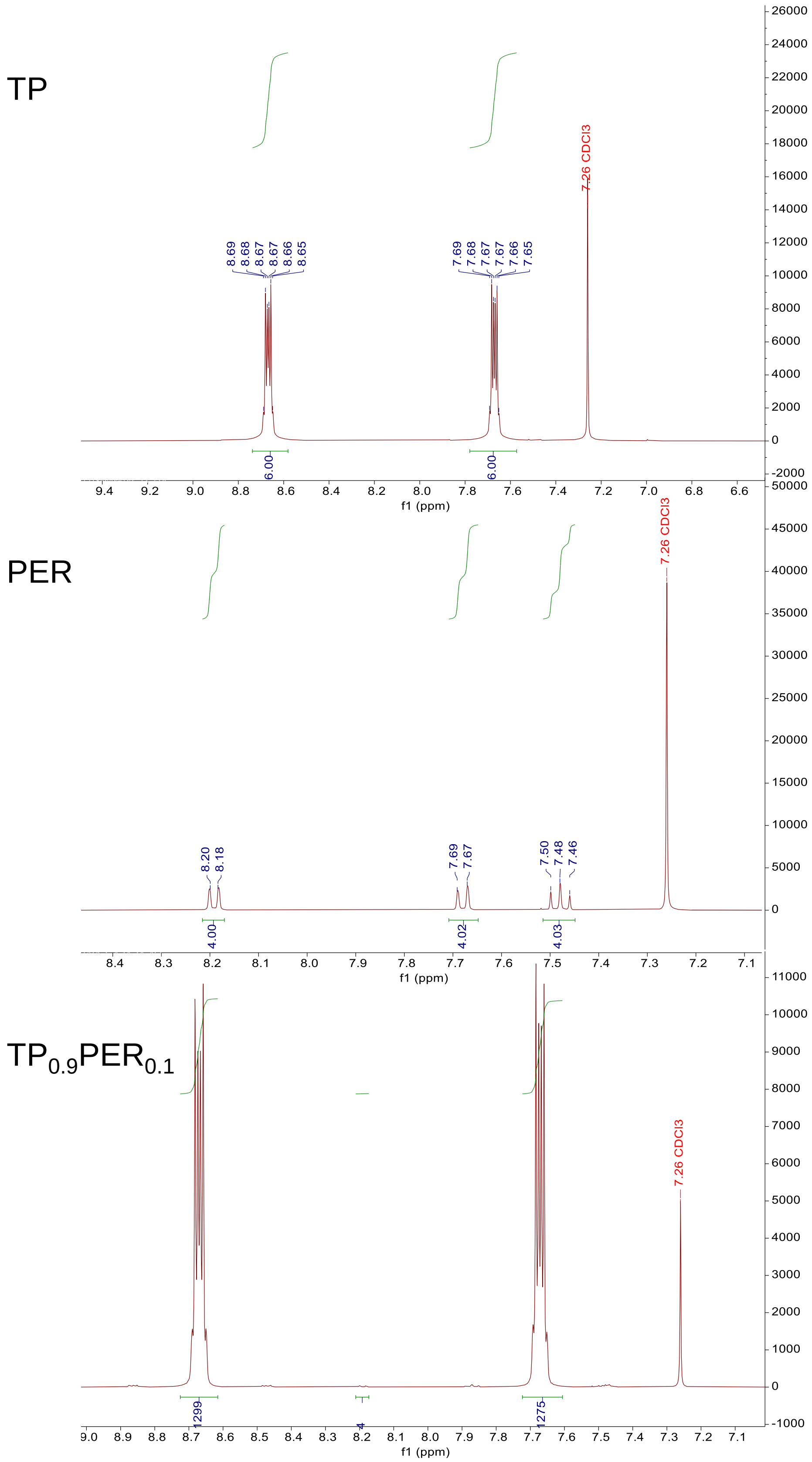

Figure S8. ^{1}H NMR spectrum of TP, PER and $TP_{0.9}PER_{0.1}$ single crystals in $CDCl_3$-d_6 (400 MHz).

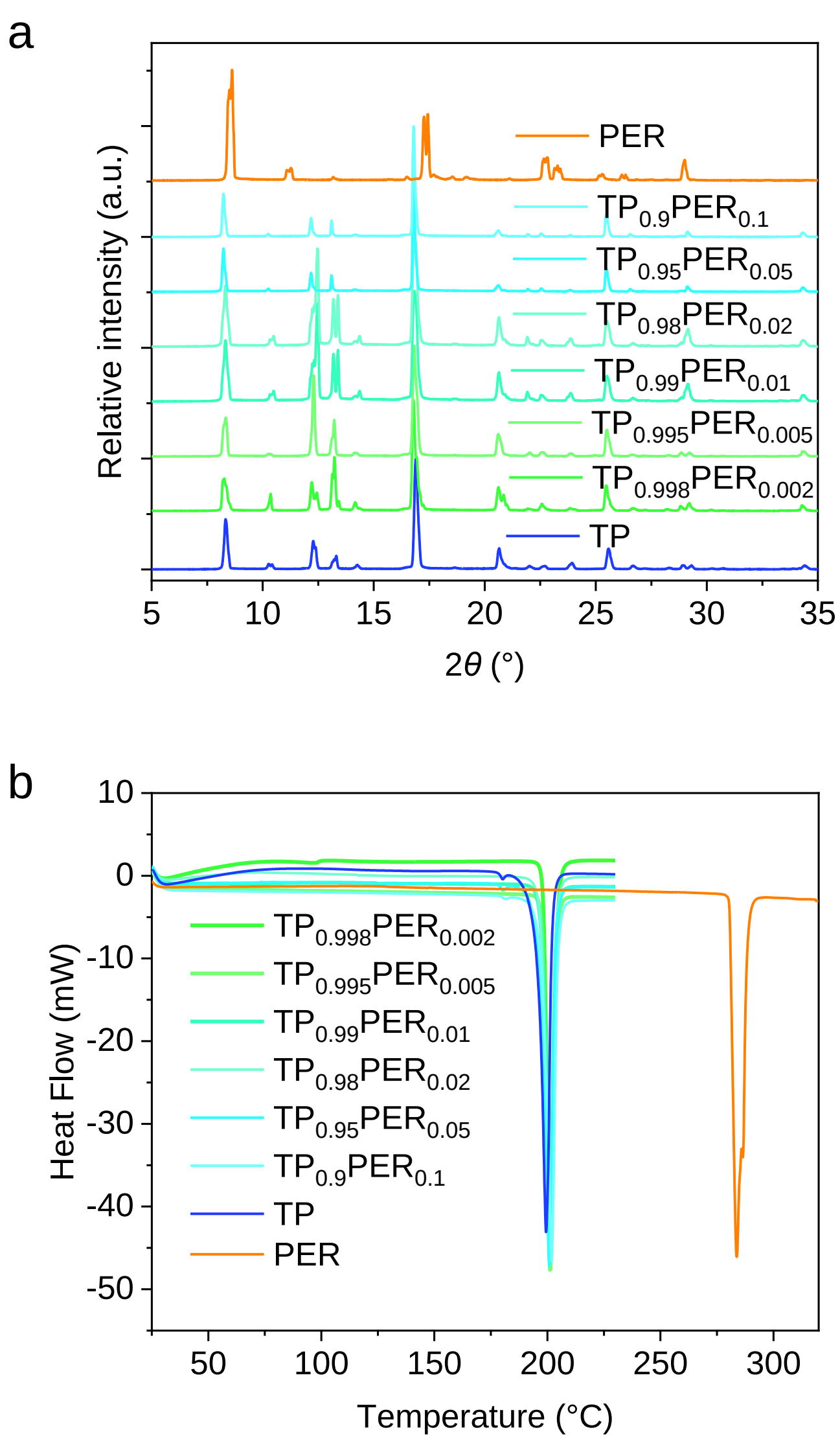

Figure S9. PXRD patterns (a) and DSC profiles (b) of TP, PER and alloy crystals.

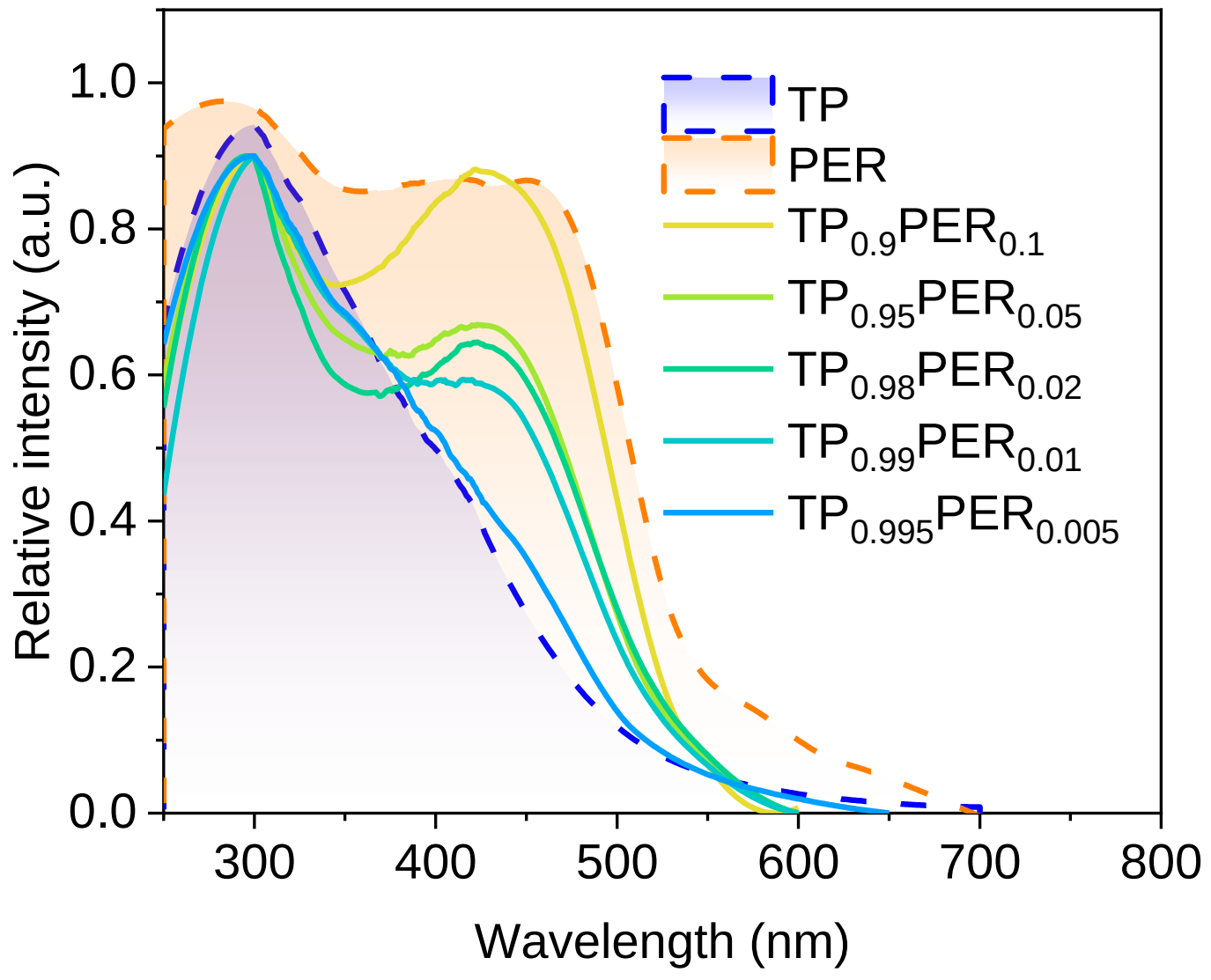

Figure S10. Ultraviolet-visible absorption spectra of PER, TP and alloy crystals.

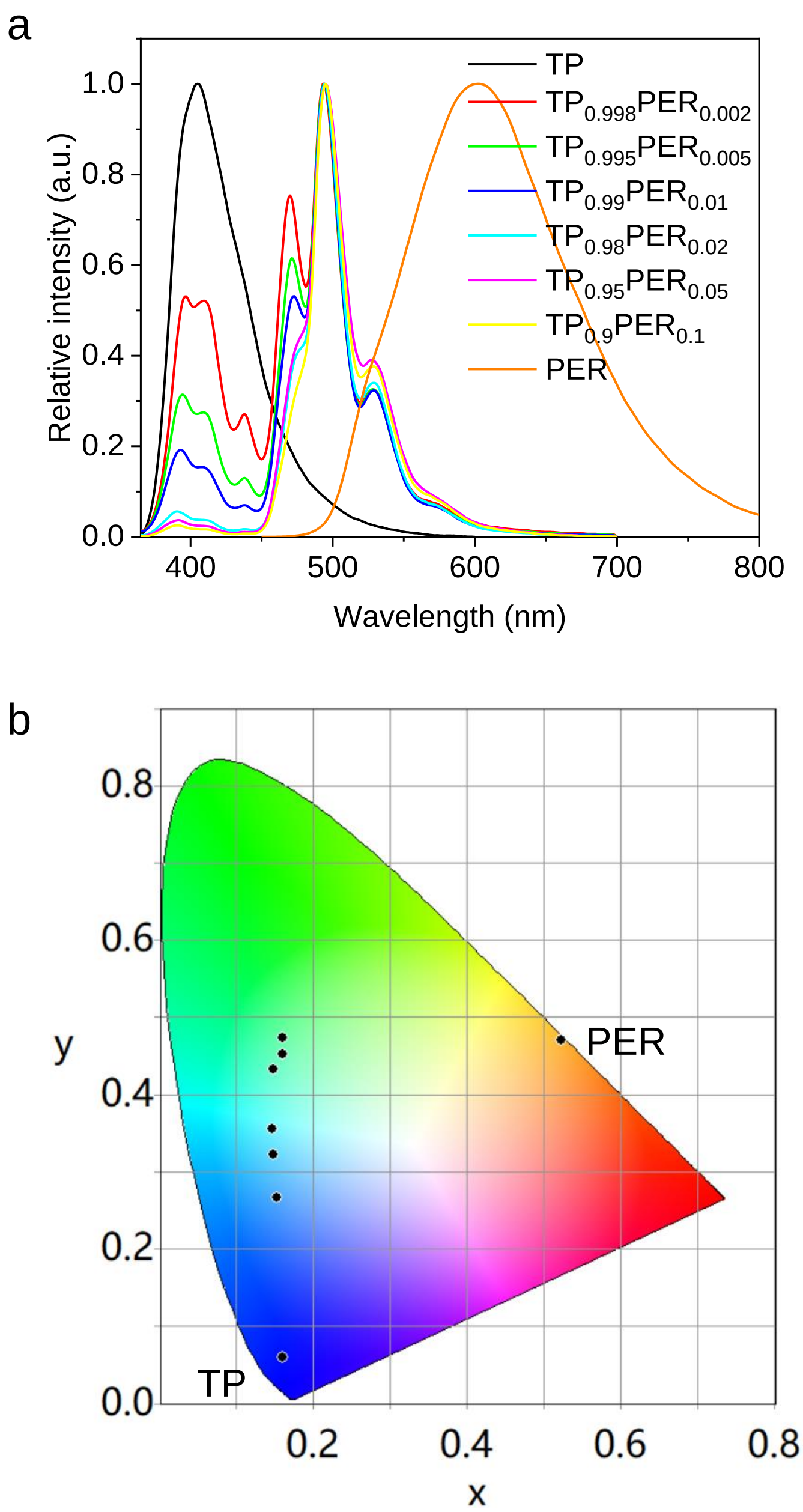

Figure S11. Photoluminescence spectra (a) and corresponding CIE chromaticity maps (b) of TP, PER and alloy crystals.

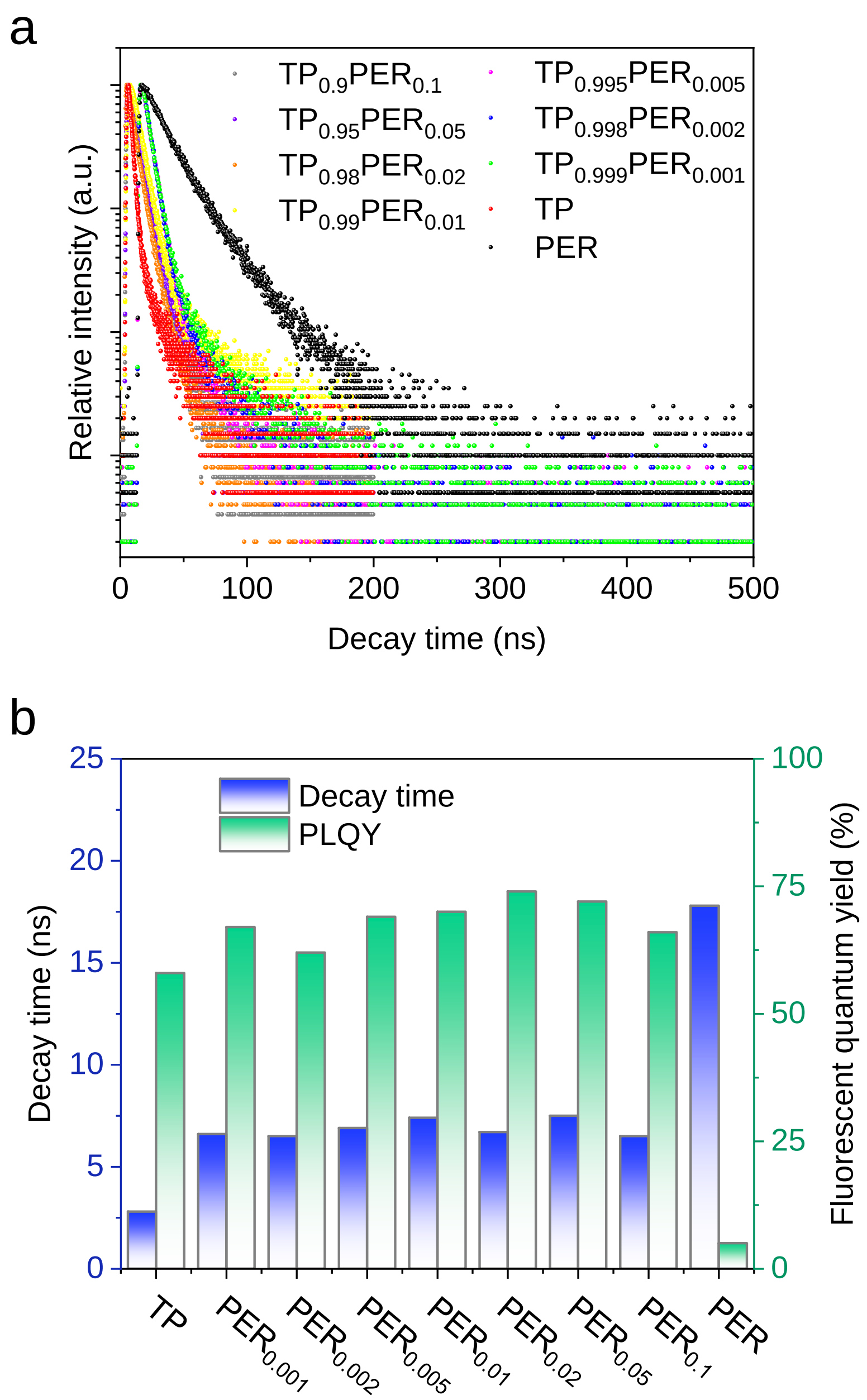

Figure S12. (a) Time-resolved fluorescence decay profiles of PER, TP and alloy crystals. (b) Statistics of fluorescence emission lifetime and fluorescence quantum yield of PER, TP and alloy crystals.

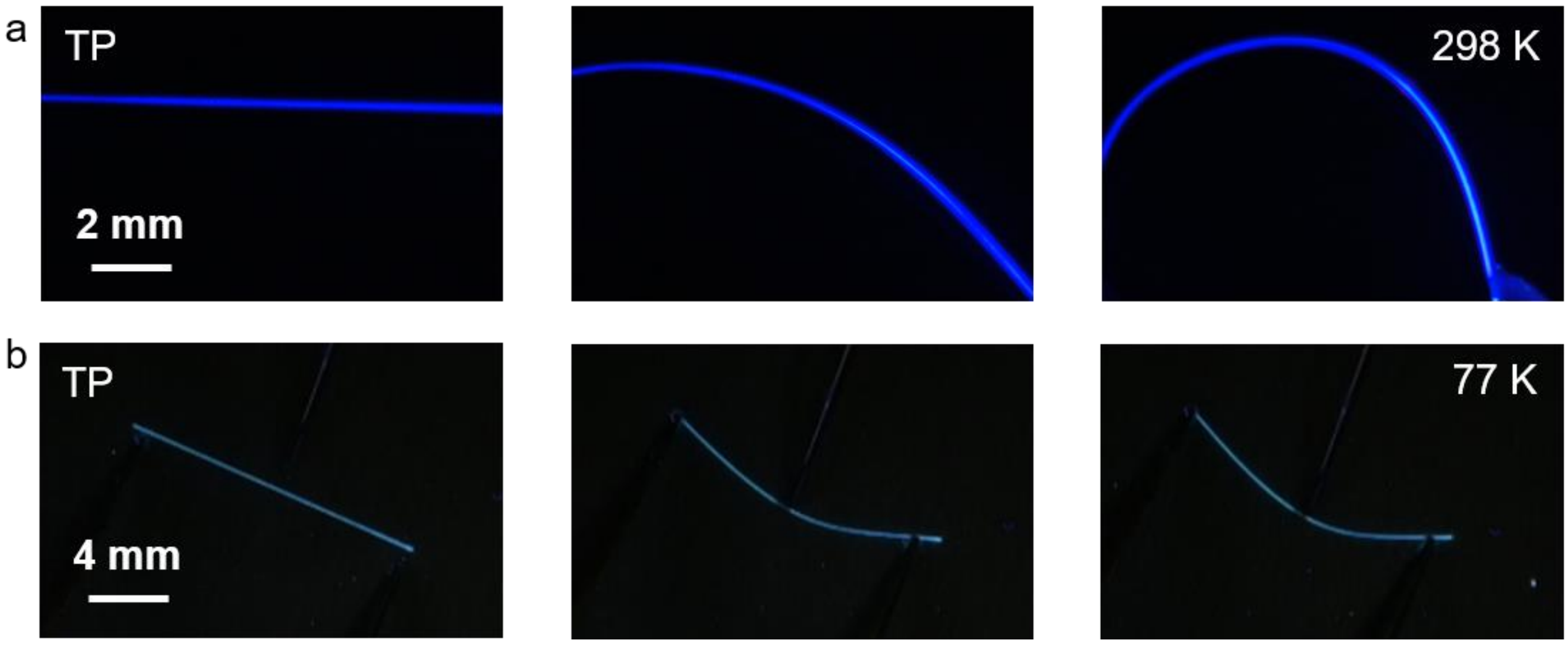

Figure S13. Elastic bending of TP crystals under room temperature (a) and low temperature (b) under UV radiation (365 nm).

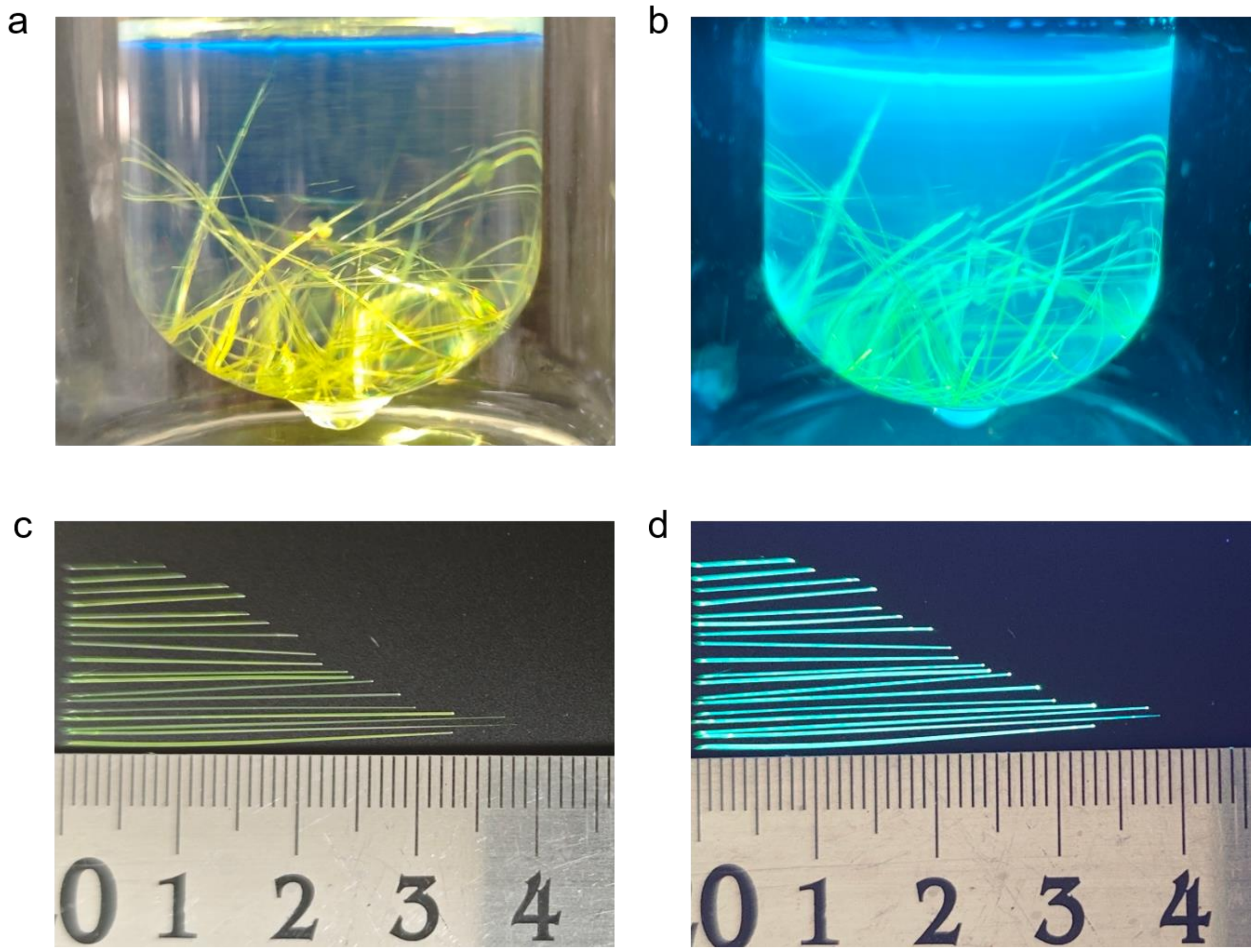

Figure S14. Photographs of $TP_{0.9}PER_{0.1}$ alloy single crystals under daylight (a, c) and under UV radiation (b, d; 365 nm).

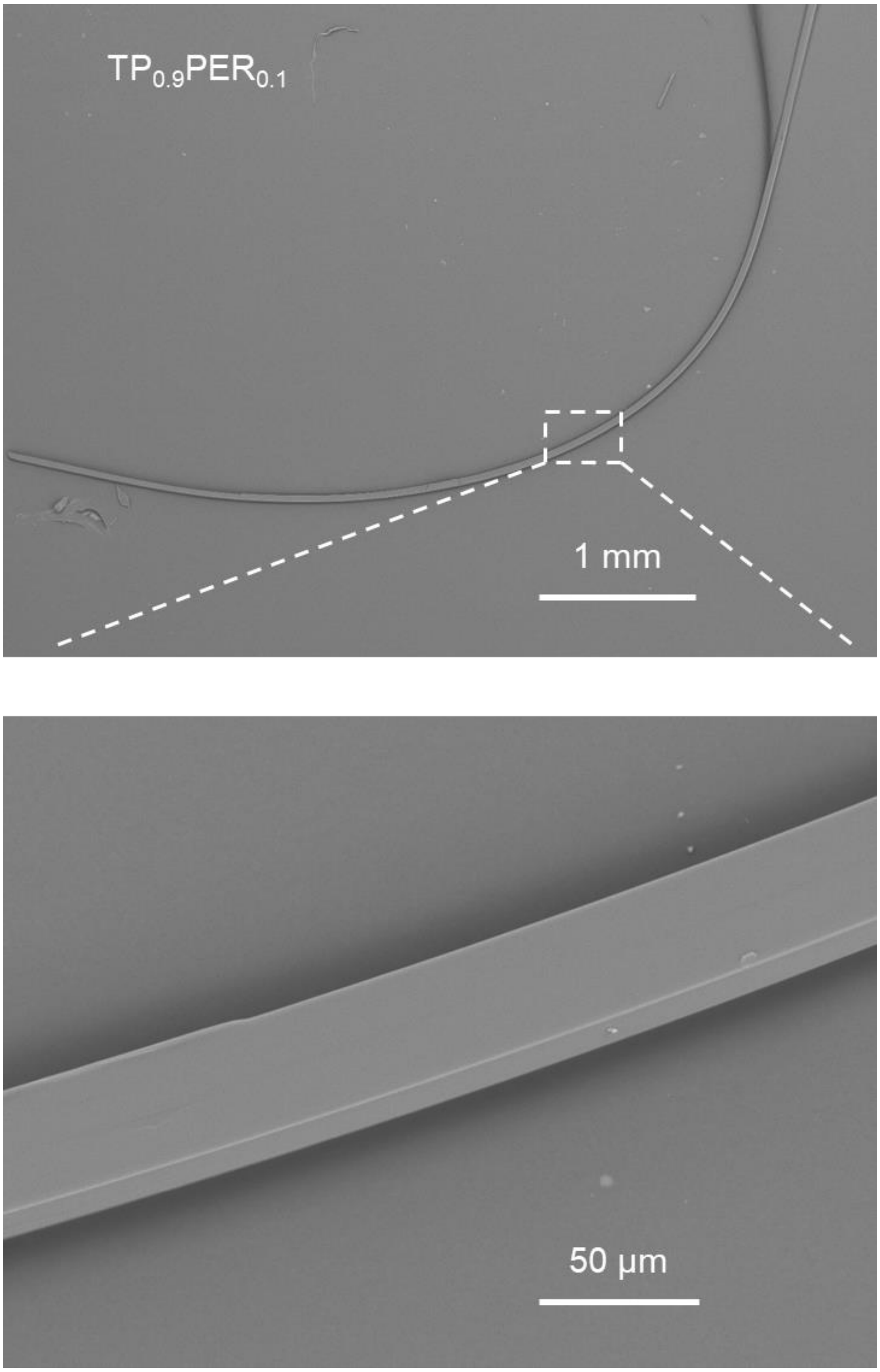

Figure S15. Scanning electron microscopy (SEM) images of $TP_{0.9}PER_{0.1}$ single crystal that has been bent 50 times.

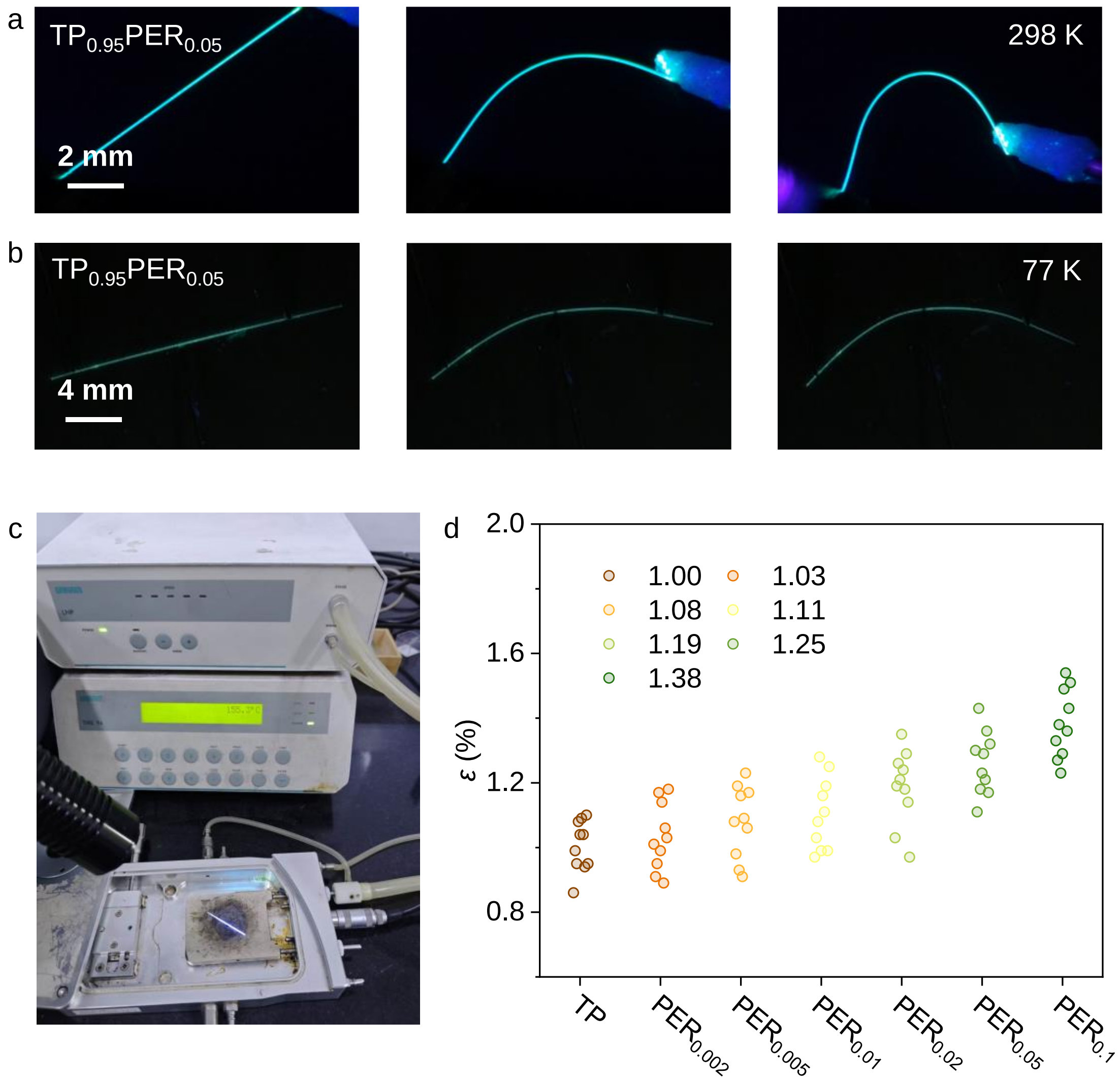

Figure S16. (a-b) Elastic bending of $TP_{0.95}PER_{0.05}$ crystals under room temperature (a) and ultra-low temperature (b) under UV radiation (365 nm). (c) Elastic bending test of alloy crystals under hot stage under UV radiation (365 nm). (d) Statistical analysis of the maximum elastic strain (ε) as a function of the composition of the alloy crystals at 77 K. The values in the plot are the average values in %.

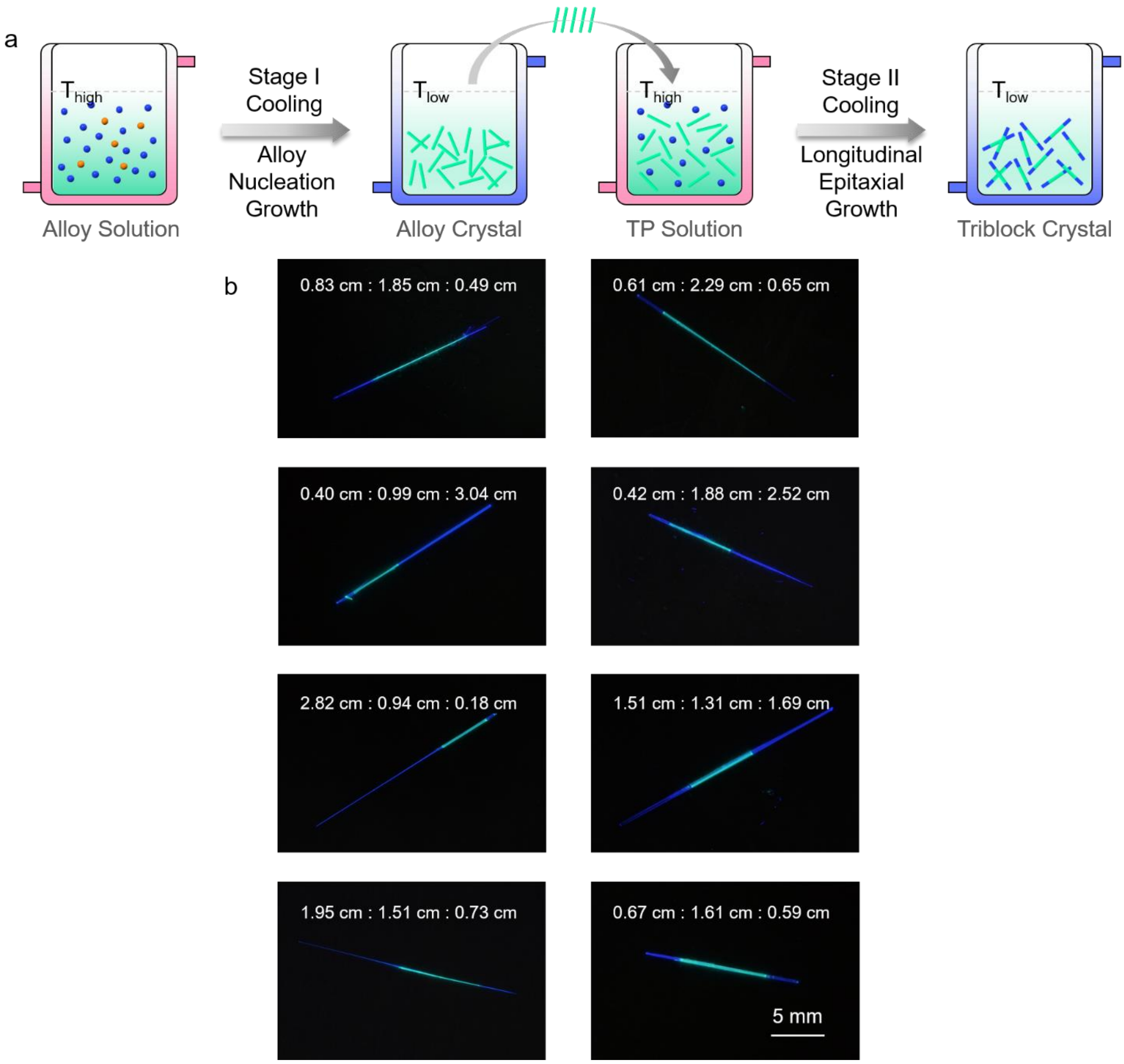

Figure S17. (a) Schematic illustrations depicting the preparation of the organic single–crystal heterostructures. (b) Fluorescence images of the blue-cyan-blue triblock crystals directly obtained through solution cooling crystallization.

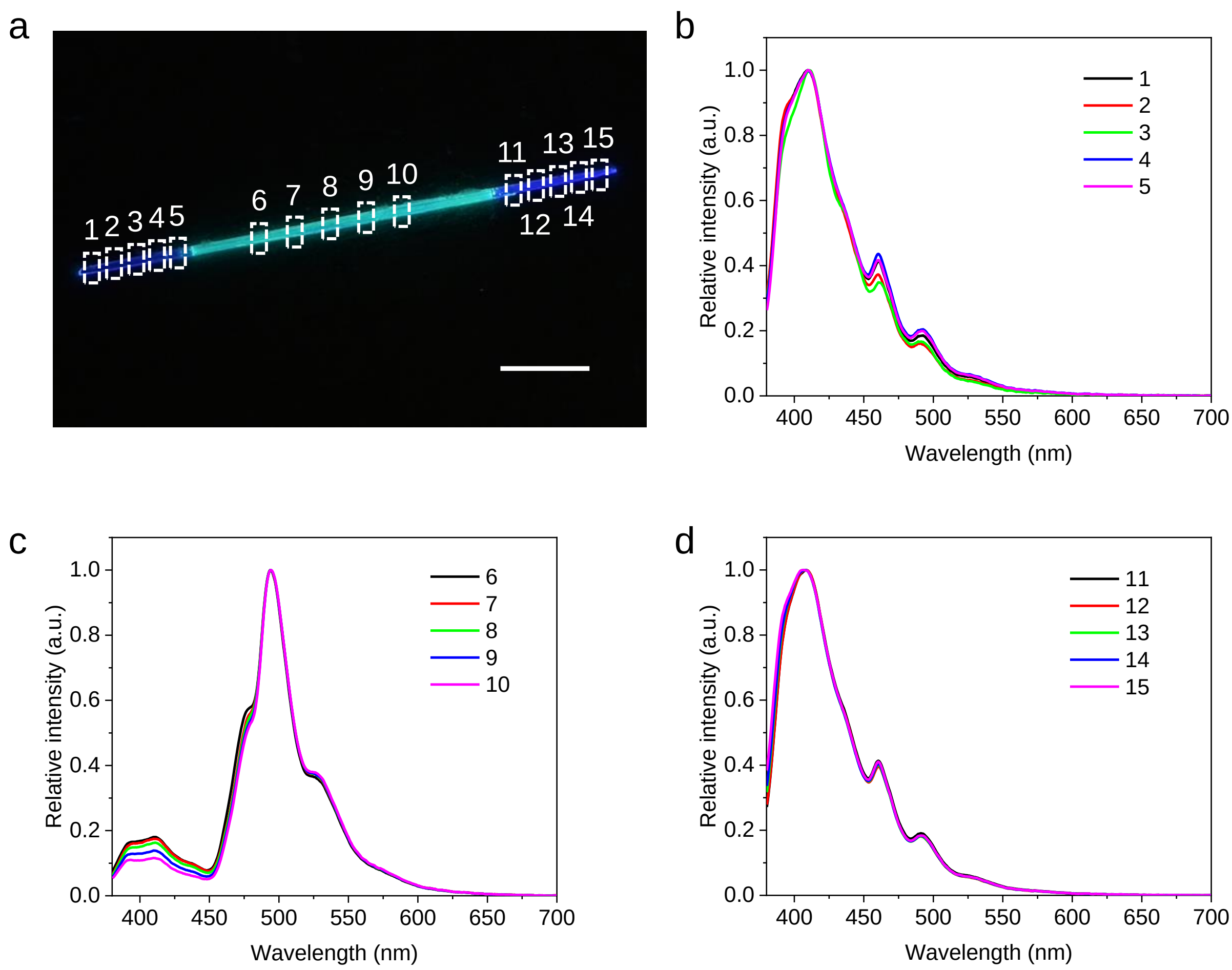

Figure S18. Optical characterization of triblock organic single–crystal heterostructures. (a) Fluorescence images of blue-cyan-blue triblock crystal. The scale bar is 5 mm. (b-d) Photoluminescence spectra collected from different sections marked in fluorescence images of triblock crystal excited at λ = 365 nm.

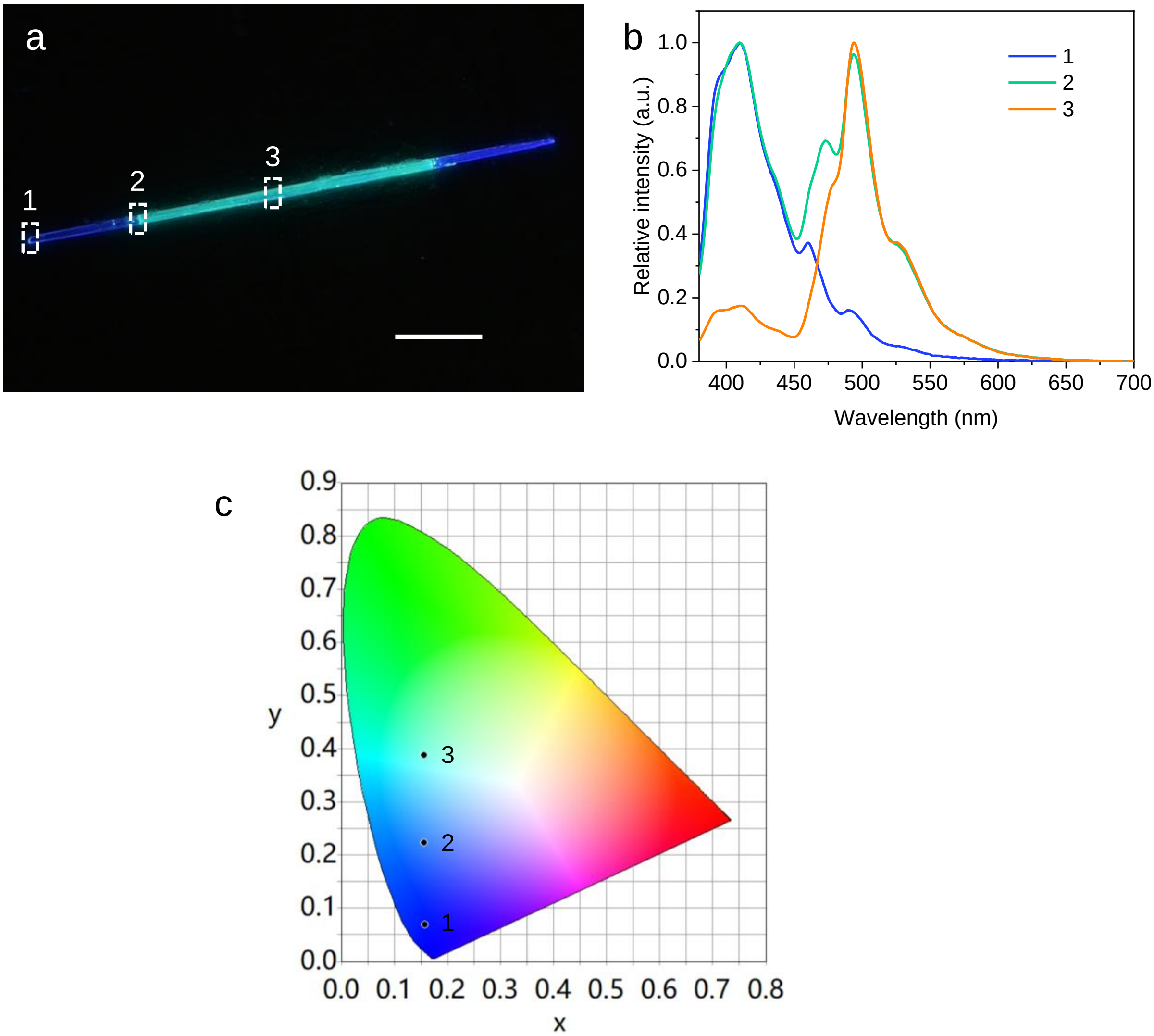

Figure S19. Optical characterization of triblock organic single–crystal heterostructures. (a) Fluorescence images of blue-cyan-blue triblock crystal. The scale bar is 5 mm. (b) Photoluminescence spectra collected from different sections marked in fluorescence images of triblock crystal excited at λ = 365 nm. (c) The CIE chromaticity diagrams for the prepared crystal shown in (a).

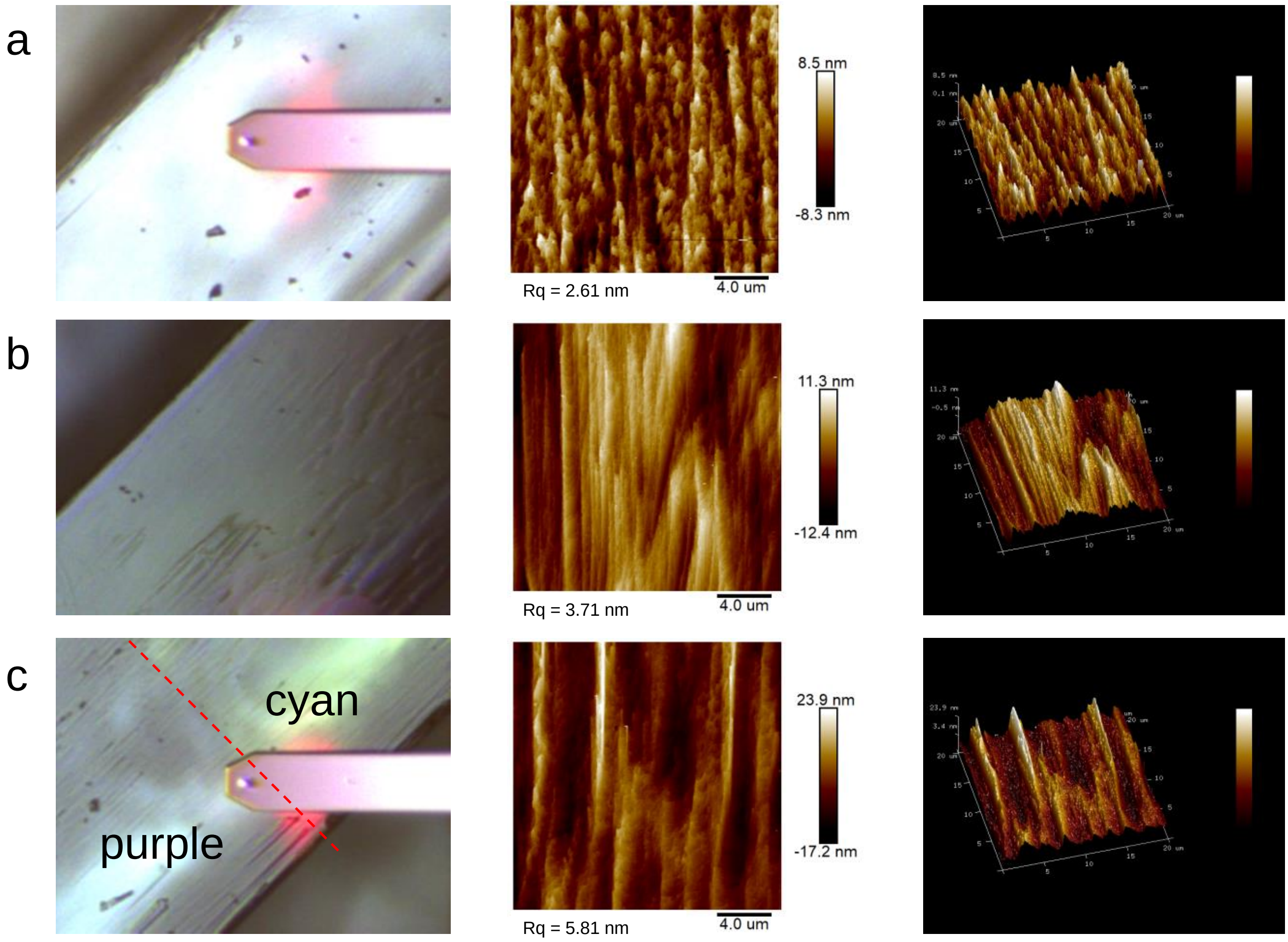

Figure S20. AFM images of the organic crystal heterostructure. (a) AFM image of the blue-emitting block, with a roughness of 2.61 nm. (b) AFM image of the cyan-emitting block, with a roughness of 3.71 nm. (c) AFM image near the interface of the crystal heterostructure, with a roughness of 5.81 nm.

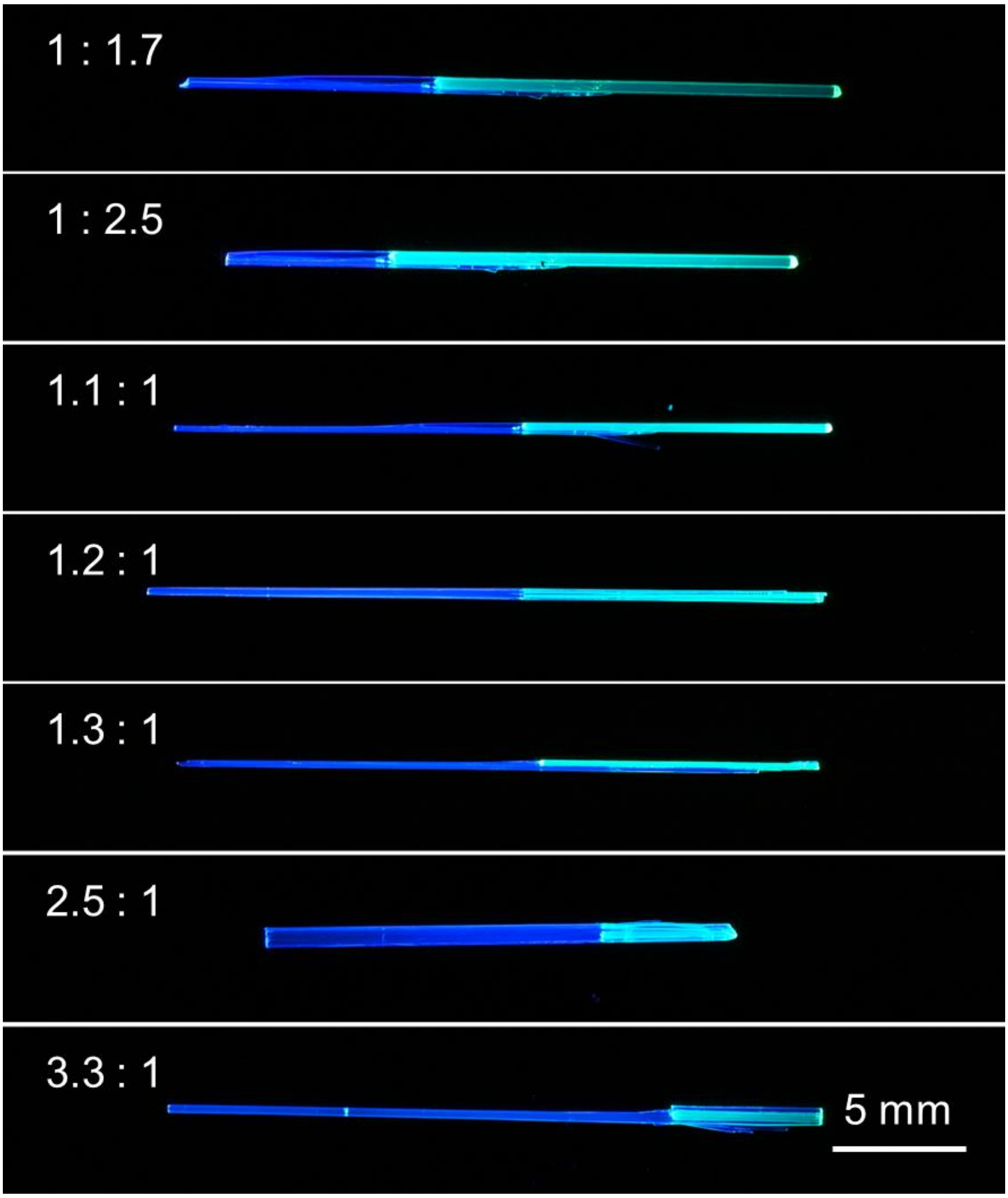

Figure S21. Fluorescence images of the blue-cyan diblock crystals obtained through manual cutting with different ratio of length.

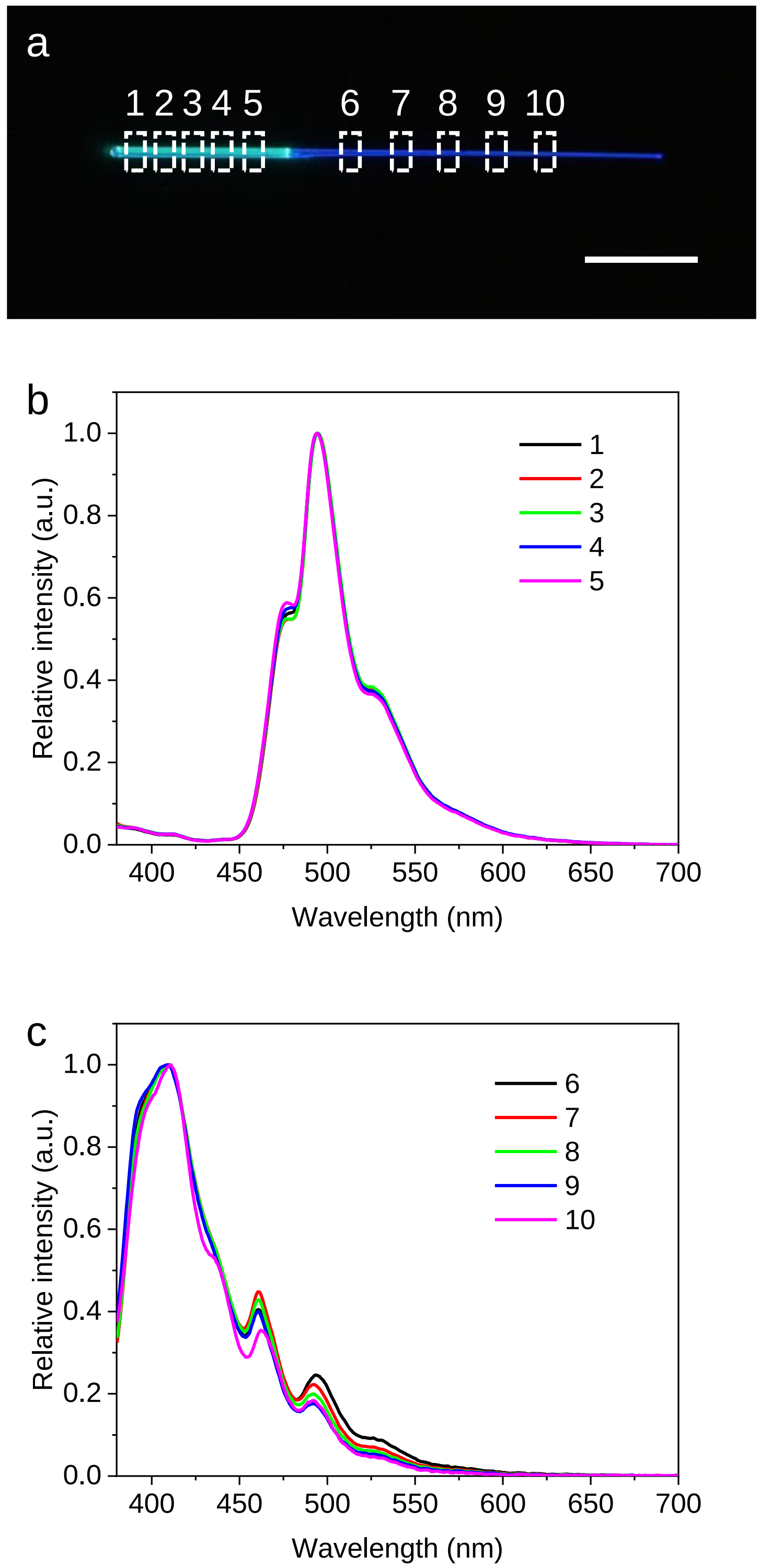

Figure S22. Optical characterization of diblock organic single–crystal heterostructures. (a) Fluorescence images of blue-cyan diblock crystal. The scale bar is 5 mm. (b, c) Photoluminescence spectra collected from different sections marked in fluorescence images of diblock crystal excited at λ = 365 nm. Photoluminescence spectra acquired at different positions within the end and middle sections were nearly identical, further confirming compositional uniformity within each block.

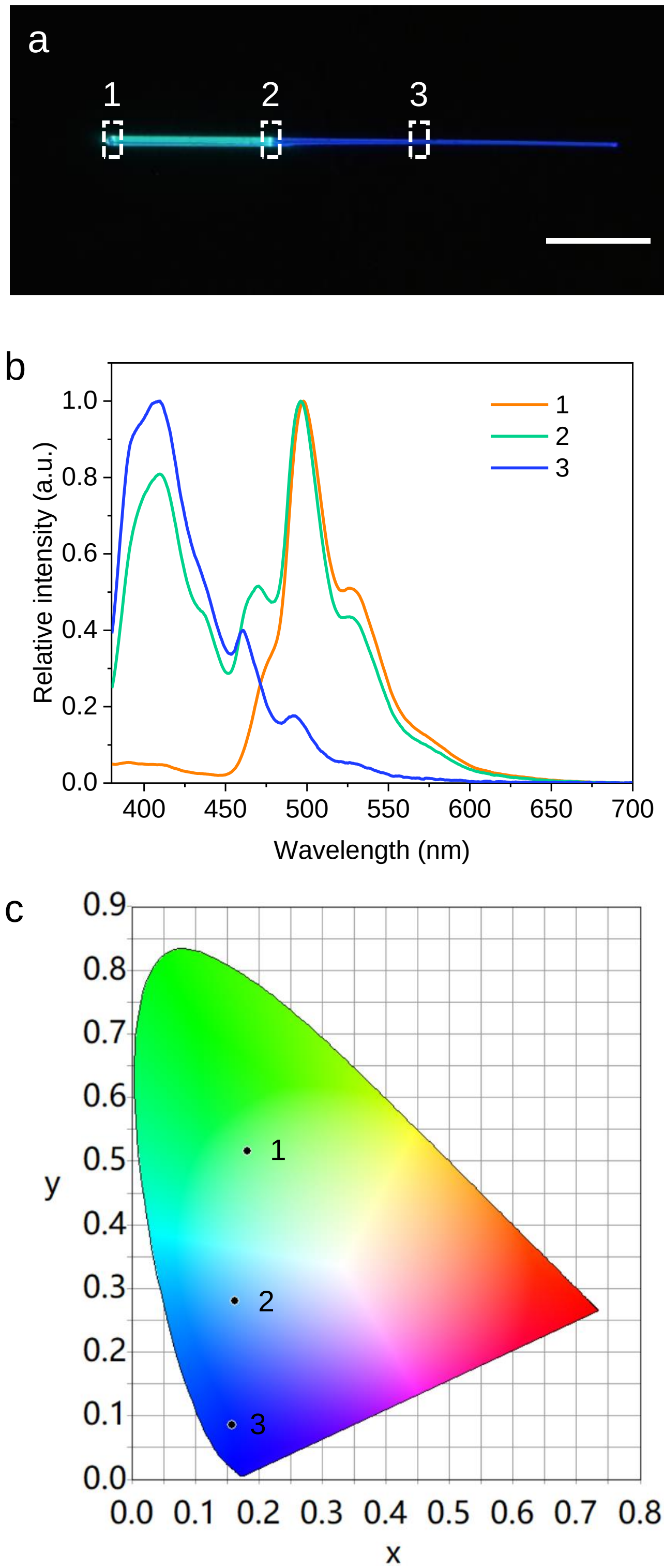

Figure S23. Optical characterization of diblock organic single–crystal heterostructures. (a) Fluorescence images of blue-cyan diblock crystal. The scale bar is 5 mm. (b) Photoluminescence spectra collected from different sections marked in fluorescence images of diblock crystal excited at λ = 365 nm. (c) The CIE chromaticity diagrams for the prepared crystal shown in (a).

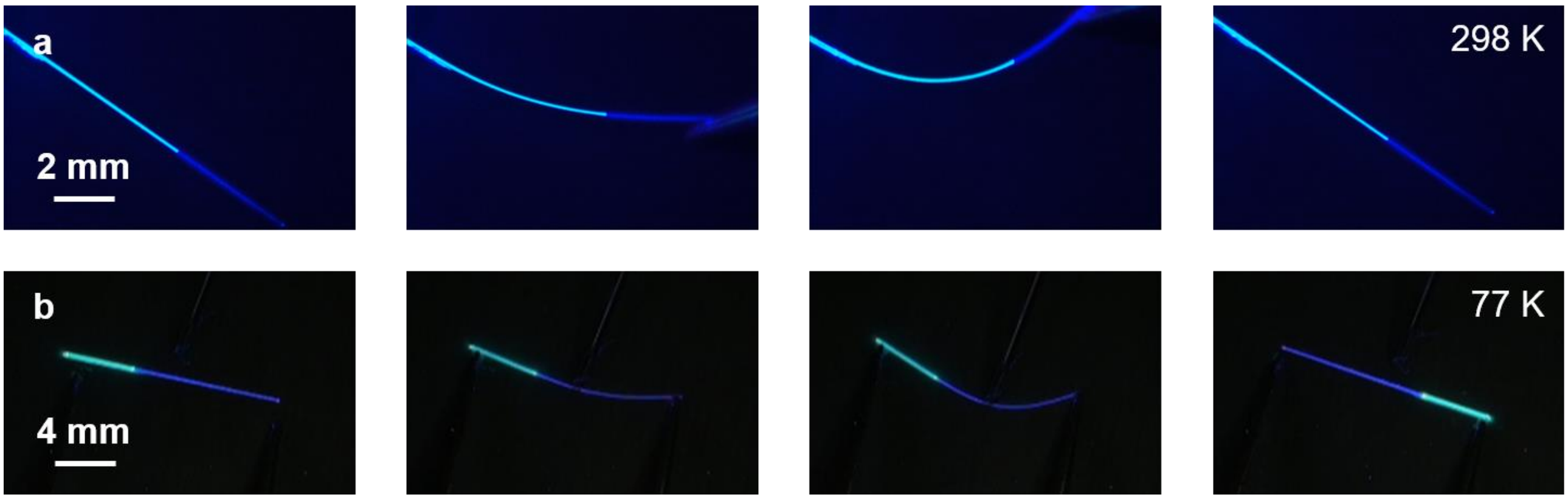

Figure S24. Elastic bending of blue-cyan diblock crystals at room temperatures (a) and low temperatures (b) under UV radiation (365 nm).

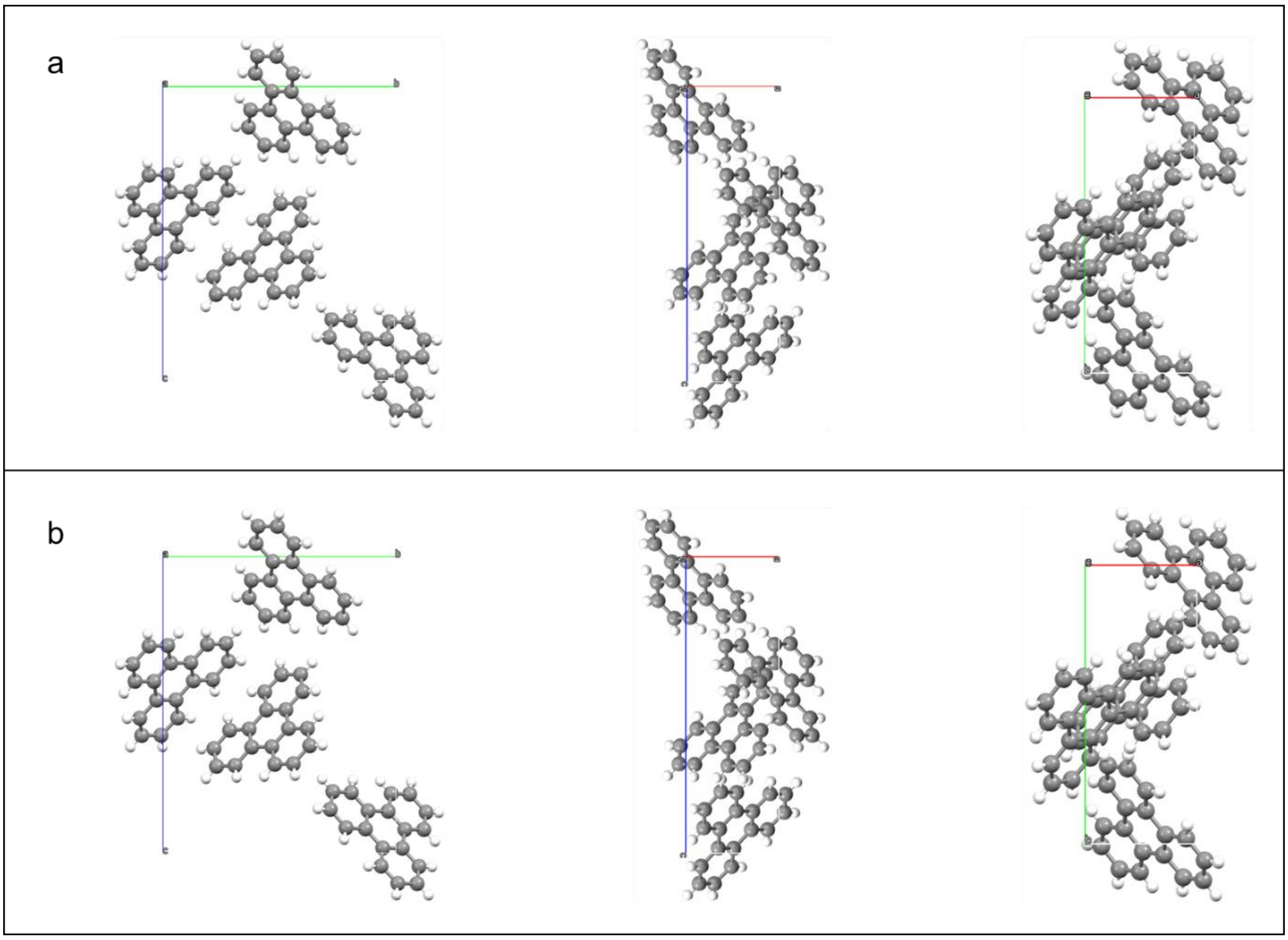

Figure S25. The molecular packing pattern of TP (a) and $TP_{0.9}PER_{0.1}$ (b) in *bc*, *ac*, *ab* planes.

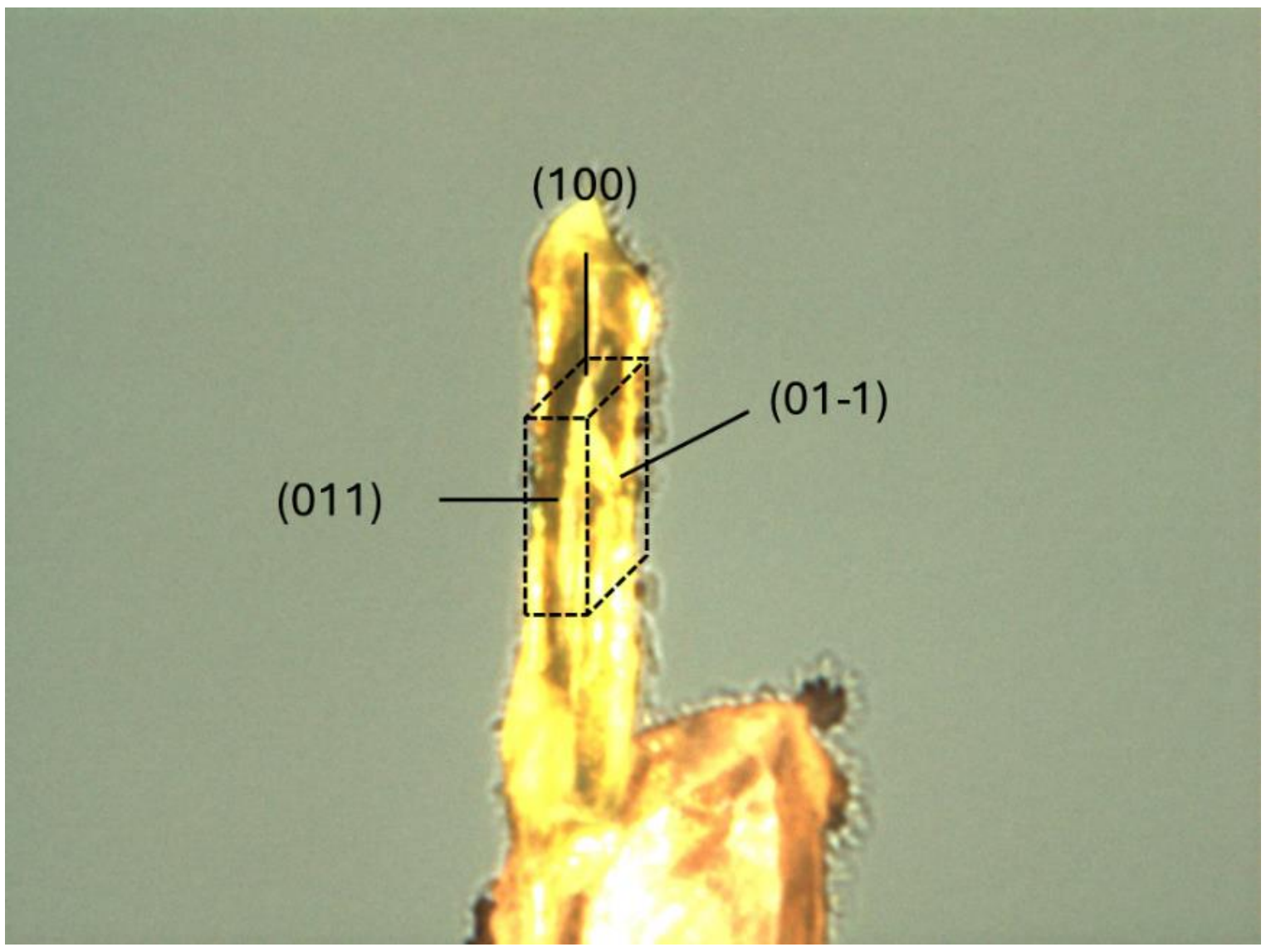

Figure S26. Face indexing of a straight crystal of $TP_{0.9}PER_{0.1}$.

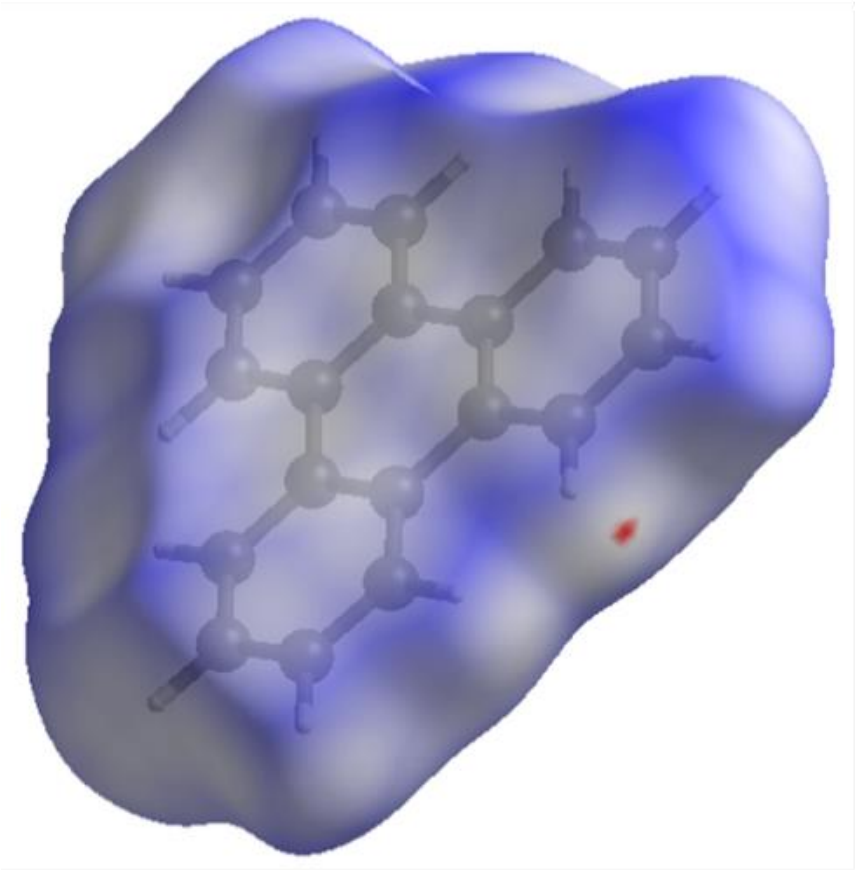

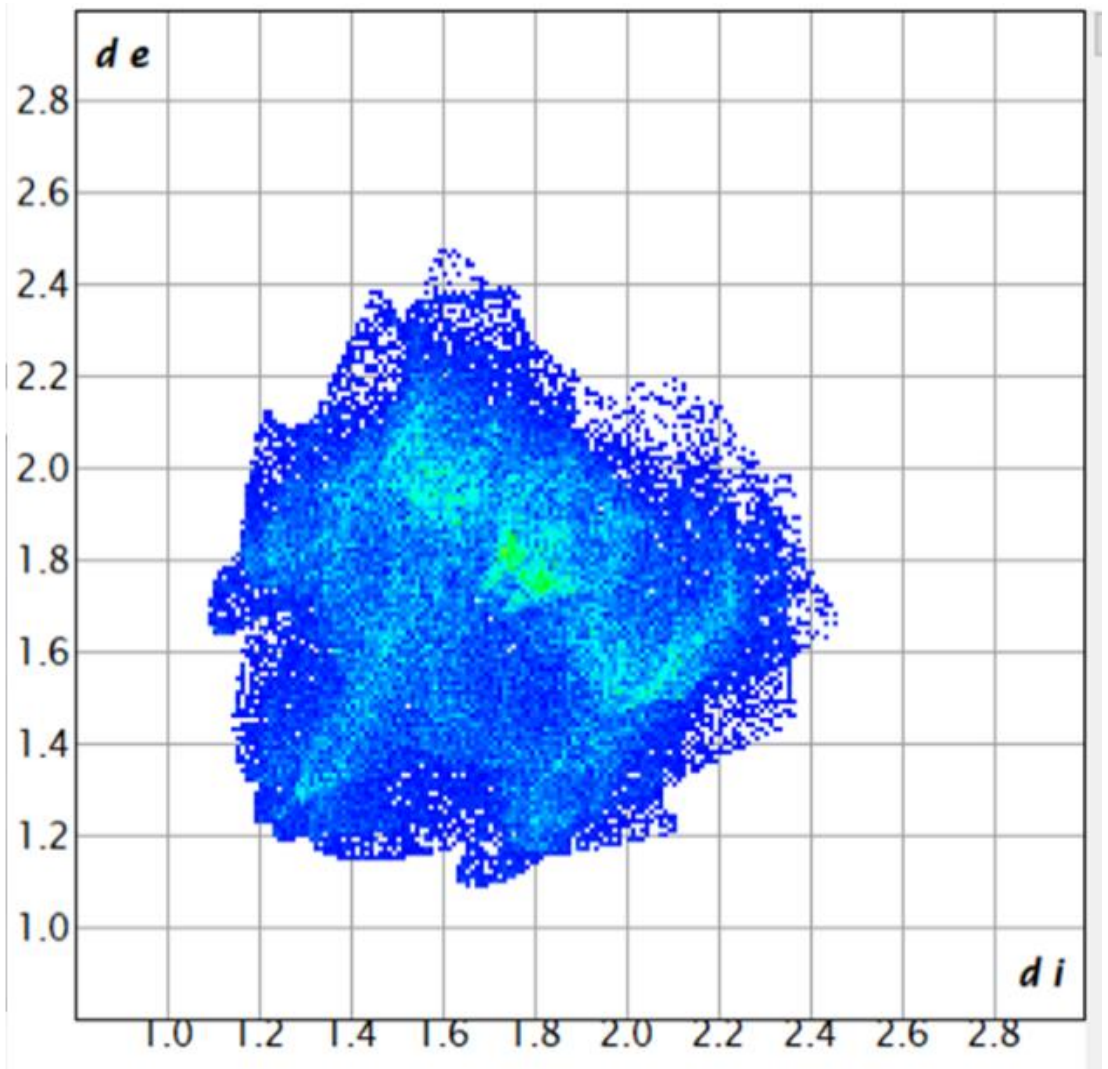

Figure S27. Hirshfeld surface (transparent) surrounding a TP molecule, alongside its corresponding 2D fingerprints based on the d_{norm} values of the different interactions.

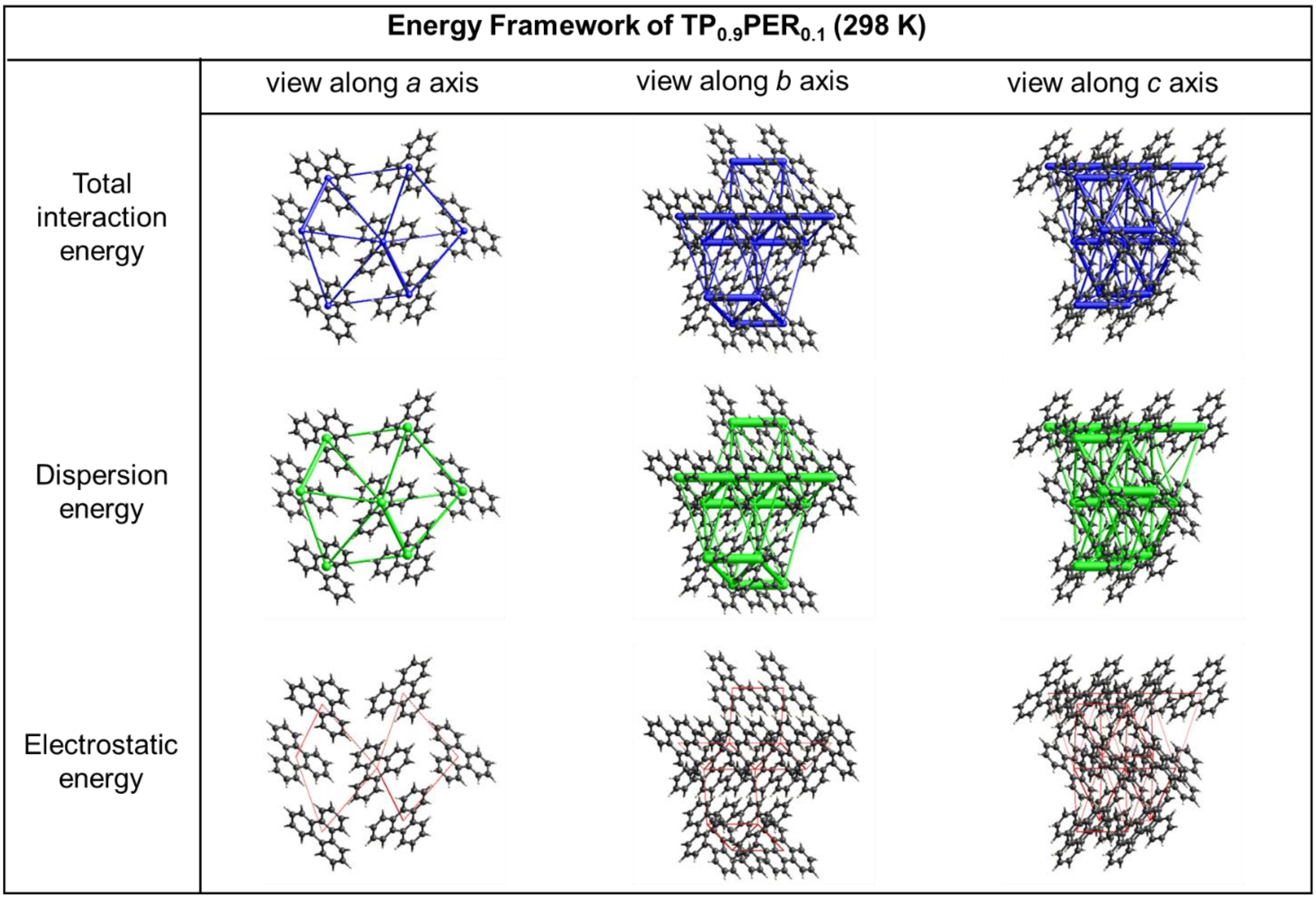

Figure S28. Three-dimensional topologies of the energy frameworks of $TP_{0.9}PER_{0.1}$ at 298 K.

	N	Symop	R	Electron Density	E_ele	E_pol	E_dis	E_rep	E_tot
	2	x, y, z	5.27	B3LYP/6-31G(d, p)	-5.2	-2.1	-58.3	32.1	-37.9
	2	-x, y+1/2, -z+1/2	10.02	B3LYP/6-31G(d, p)	-0.6	-0.2	-8.6	3.7	-6.0
	2	-x+1/2, -y+1/2, -z	8.61	B3LYP/6-31G(d, p)	-2.3	-0.4	-13.7	6.9	-10.4
	2	-x+1/2, -y, z+1/2	8.77	B3LYP/6-31G(d, p)	-1.5	-0.4	-12.9	8.4	-7.9
	2	x+1/2, -y+1/2, -z	11.39	B3LYP/6-31G(d, p)	-0.1	0.0	-1.3	0.0	-1.3
	2	-x, y+1/2, -z+1/2	8.71	B3LYP/6-31G(d, p)	-3.4	-0.5	-15.3	9.8	-11.3
	2	x+1/2, -y+1/2, -z	6.60	B3LYP/6-31G(d, p)	-5.5	-1.1	-28.5	16.9	-21.1
	2	-x+1/2, -y, -z+1/2	8.95	B3LYP/6-31G(d, p)	-1.5	-0.3	-10.3	4.0	-8.4

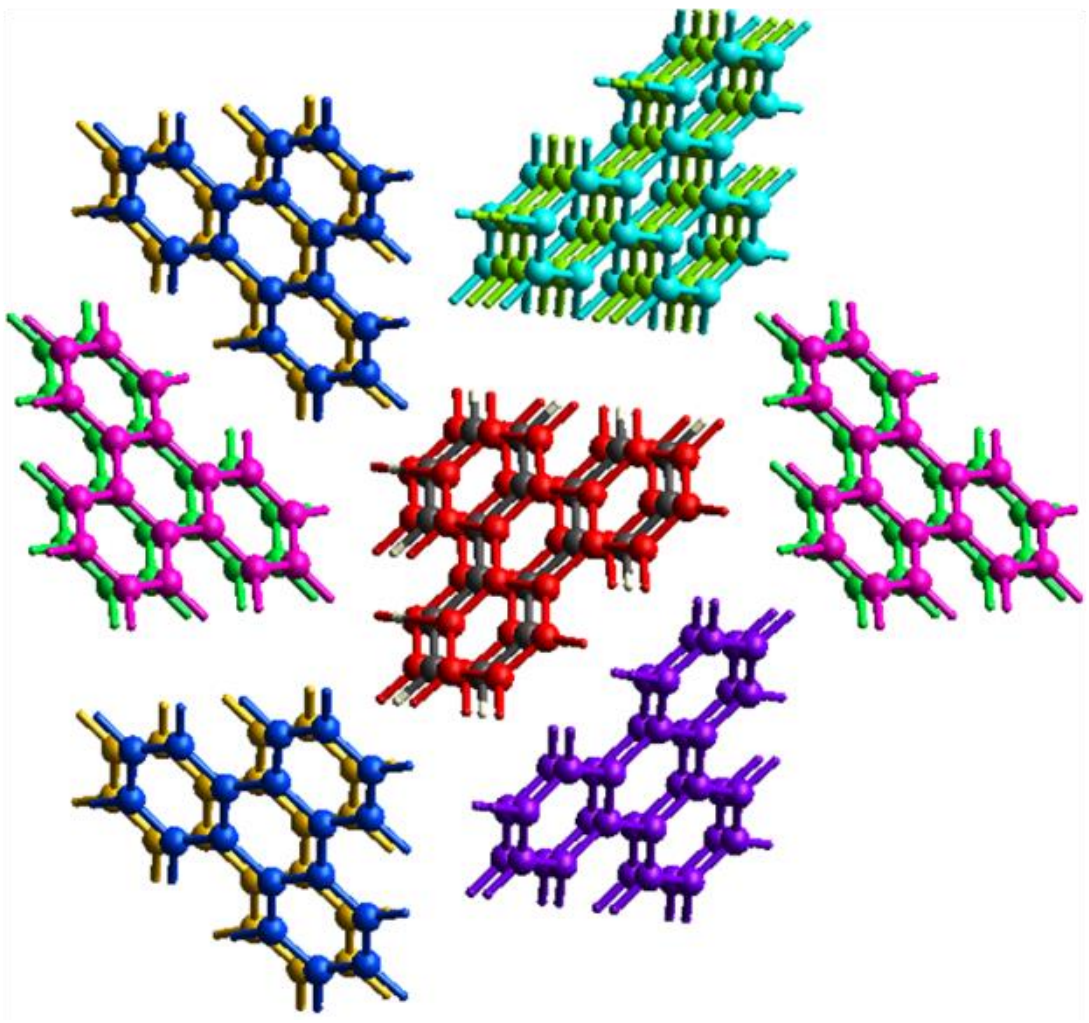

Figure S29. Molecular pairs and interaction energies obtained from the energy frameworks of $TP_{0.9}PER_{0.1}$ at 298 K.

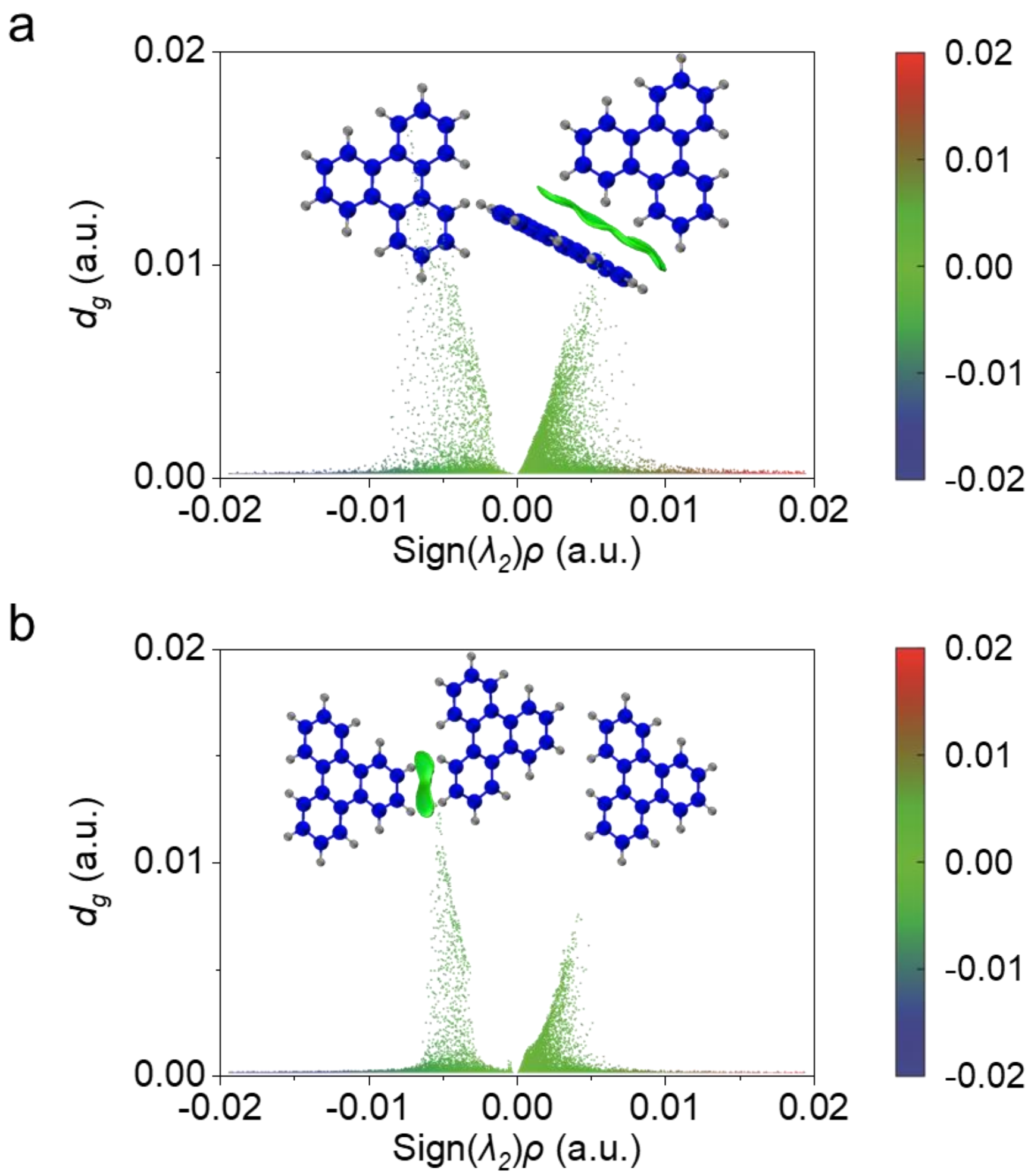

Figure S30. IGMH (independent gradient model based on Hirshfeld partition) analysis for the typical noncovalent interactions. The color-coded sign(λ_2)ρ function mapped onto the isosurfaces reveals that all molecular pairs are dominated by green regions, corresponding to weak van der Waals interactions (sign(λ_2)ρ = [−0.01, 0.01]). These weak and dispersive interactions are likely involved in the structural adjustments during elastic bending.

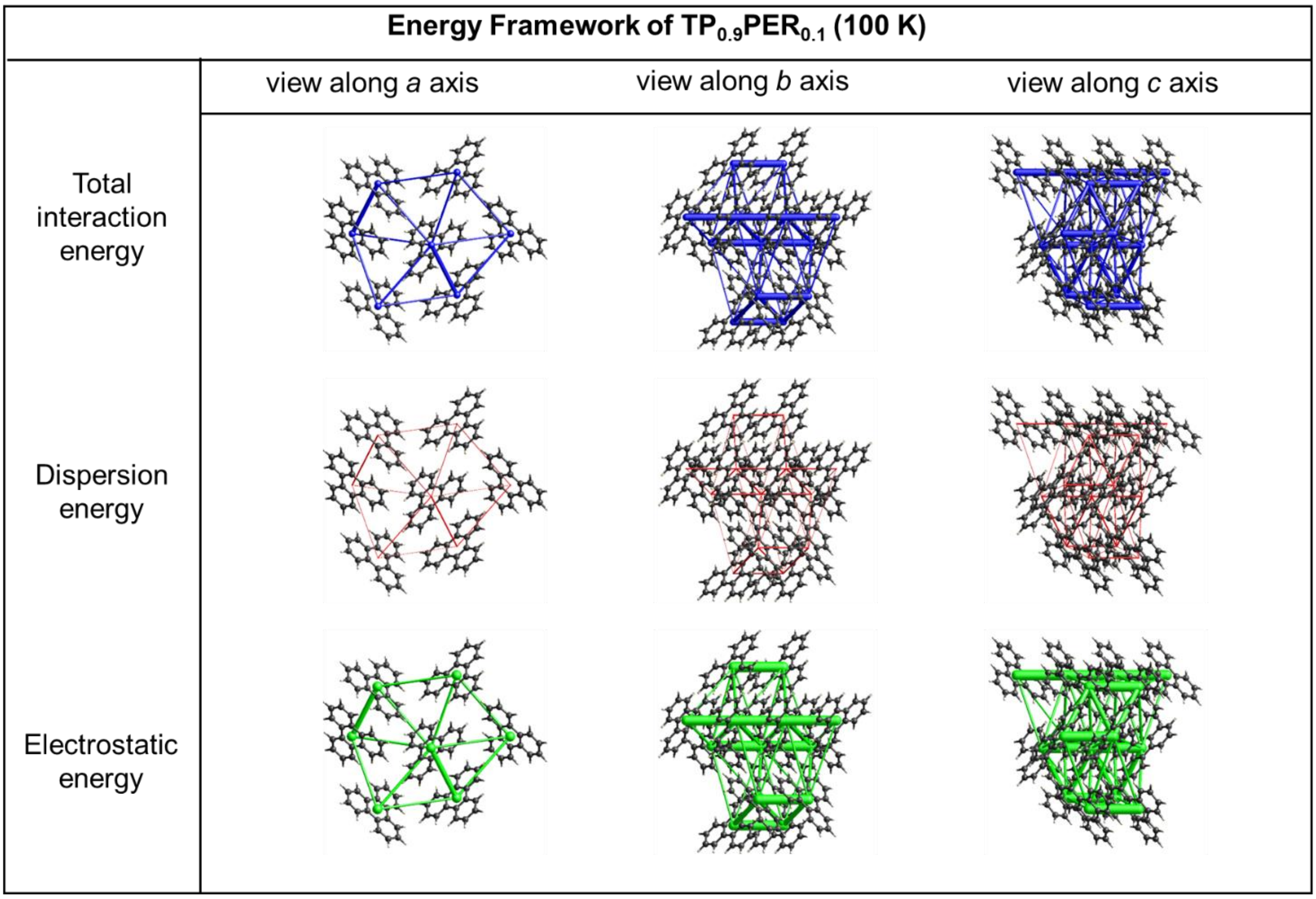

Figure S31. Three-dimensional topologies of the energy framework of $TP_{0.9}PER_{0.1}$ at 100 K.

	N	Symop	R	Electron Density	E_ele	E_pol	E_dis	E_rep	E_tot
	2	x+1/2, -y+1/2, -z	6.45	B3LYP/6-31G(d, p)	-7.3	-1.3	-32.4	22.5	-23.0
	2	x, y, z	5.26	B3LYP/6-31G(d, p)	-7.2	-2.3	-62.1	40.2	-38.6
	2	-x+1/2, -y, z+1/2	8.91	B3LYP/6-31G(d, p)	-1.9	-0.4	-11.1	5.1	-8.9
	2	-x, y+1/2, -z+1/2	8.56	B3LYP/6-31G(d, p)	-4.4	-0.6	-17.4	13.1	-12.2
	2	x+1/2, -y+1/2, -z	11.32	B3LYP/6-31G(d, p)	-0.2	0.0	-1.5	0.0	-1.5
	2	-x+1/2, -y, z+1/2	8.72	B3LYP/6-31G(d, p)	-2.0	-0.5	-13.9	10.4	-8.2
	2	x+1/2, -y+1/2, -z	8.53	B3LYP/6-31G(d, p)	-3.1	-0.4	-15.4	9.3	-11.3
	2	-x, -y+1/2, -z+1/2	9.89	B3LYP/6-31G(d, p)	-1.2	-0.2	-10.0	5.5	-6.8

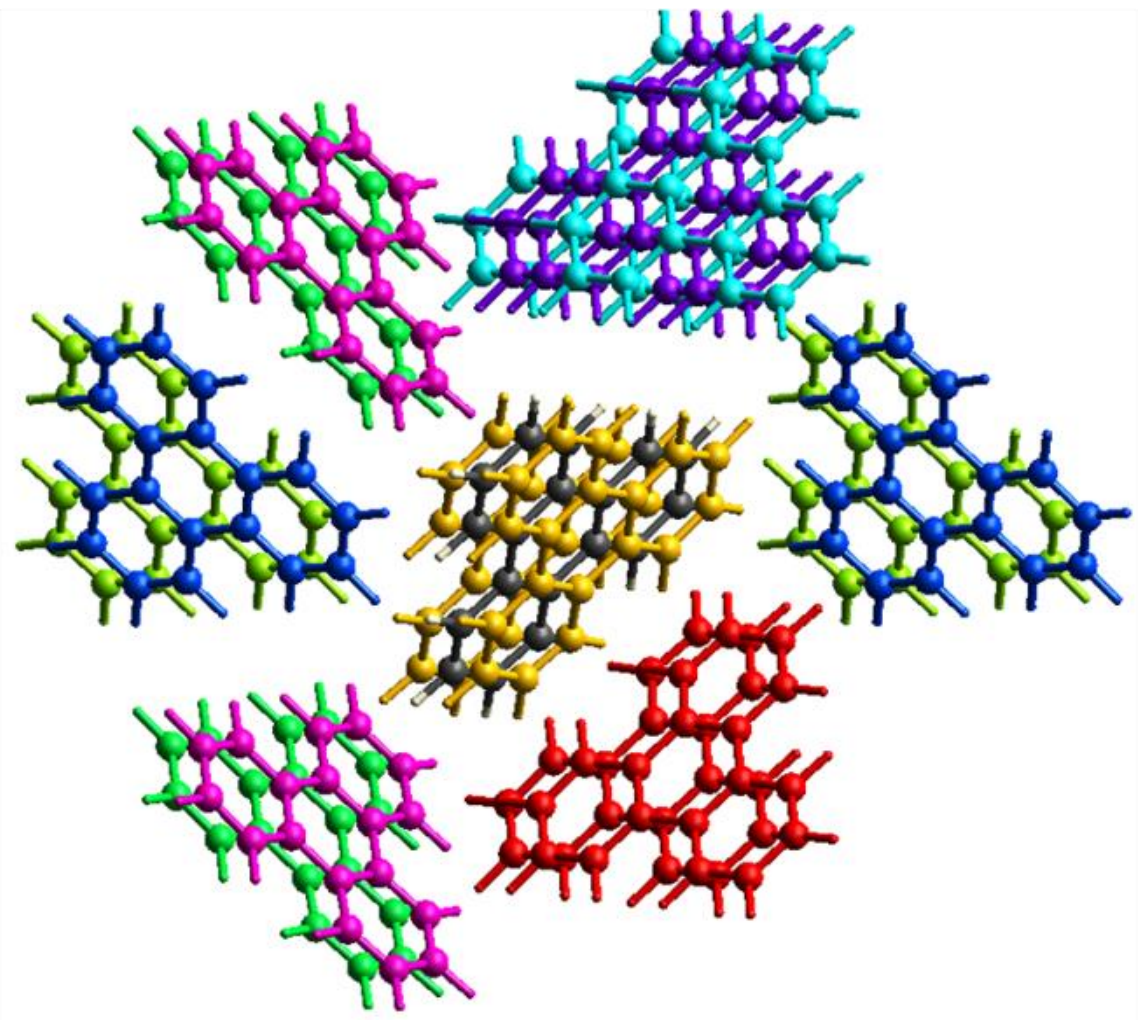

Figure S32. Molecular pairs and interaction energies obtained from the energy frameworks of $TP_{0.9}PER_{0.1}$ at 100 K.

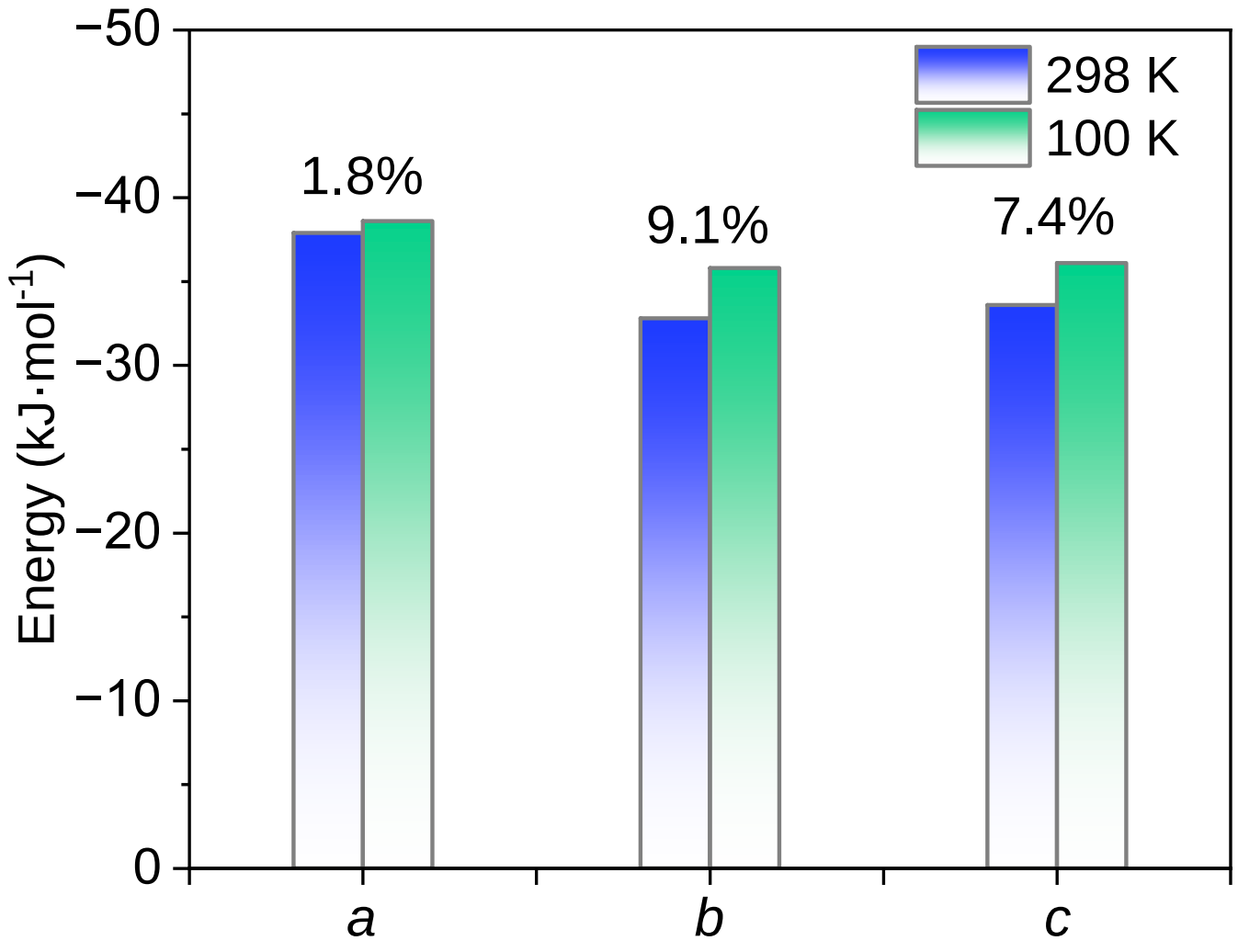

Figure S33. Comparison of the energy contributions of the main interactions at 100 and 298 K. The energy change (in %) is calculated as $(E_{100\ K} - E_{298\ K})/E_{298\ K}$, where $E_{100\ K}$ and $E_{298\ K}$ are the energies at 100 and 298 K, respectively.

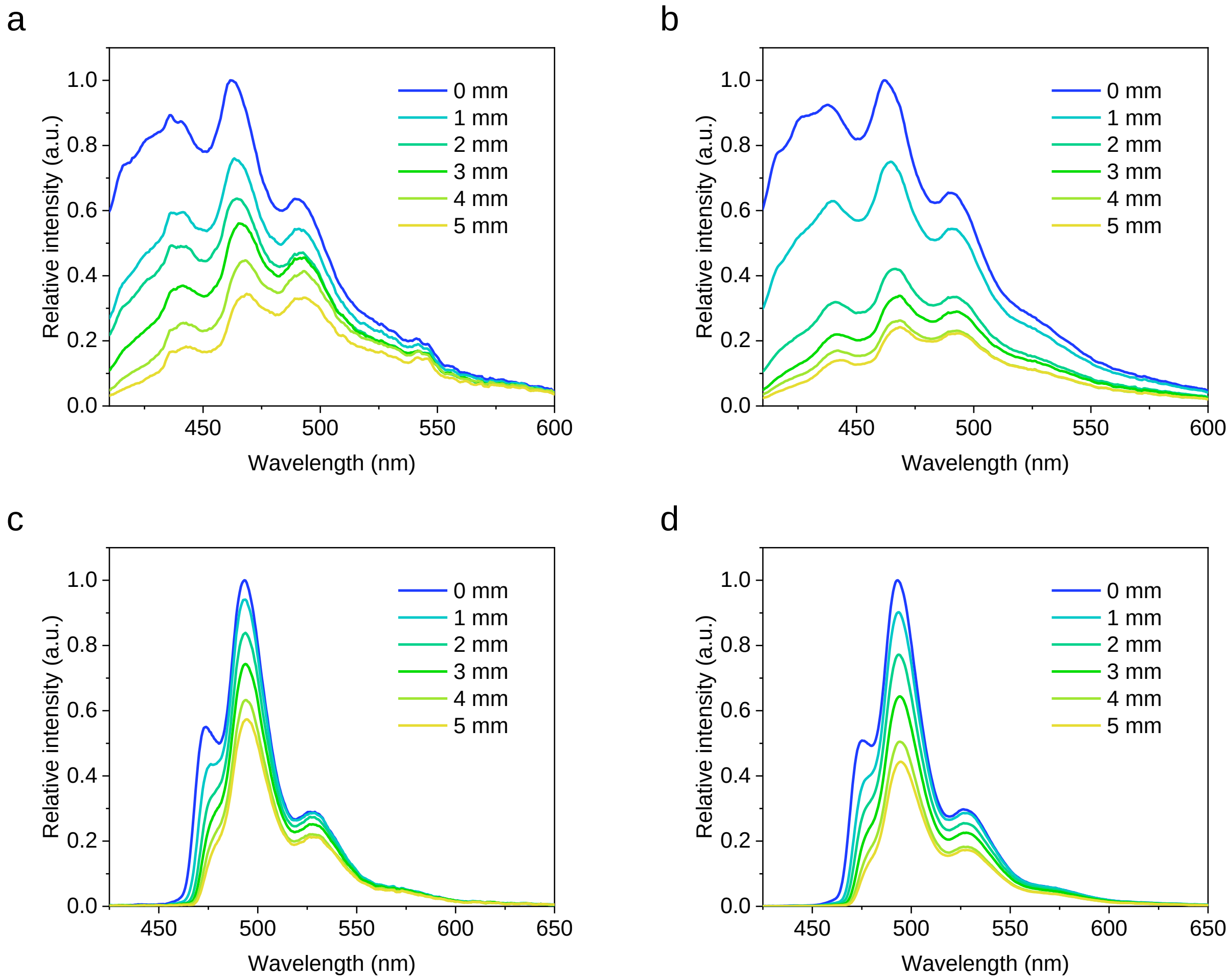

Figure S34. Fluorescence spectra collected at the tip of the TP (a, b) and $TP_{0.99}PER_{0.01}$ (c, d) crystal excited by a 365 nm laser focused at different positions.

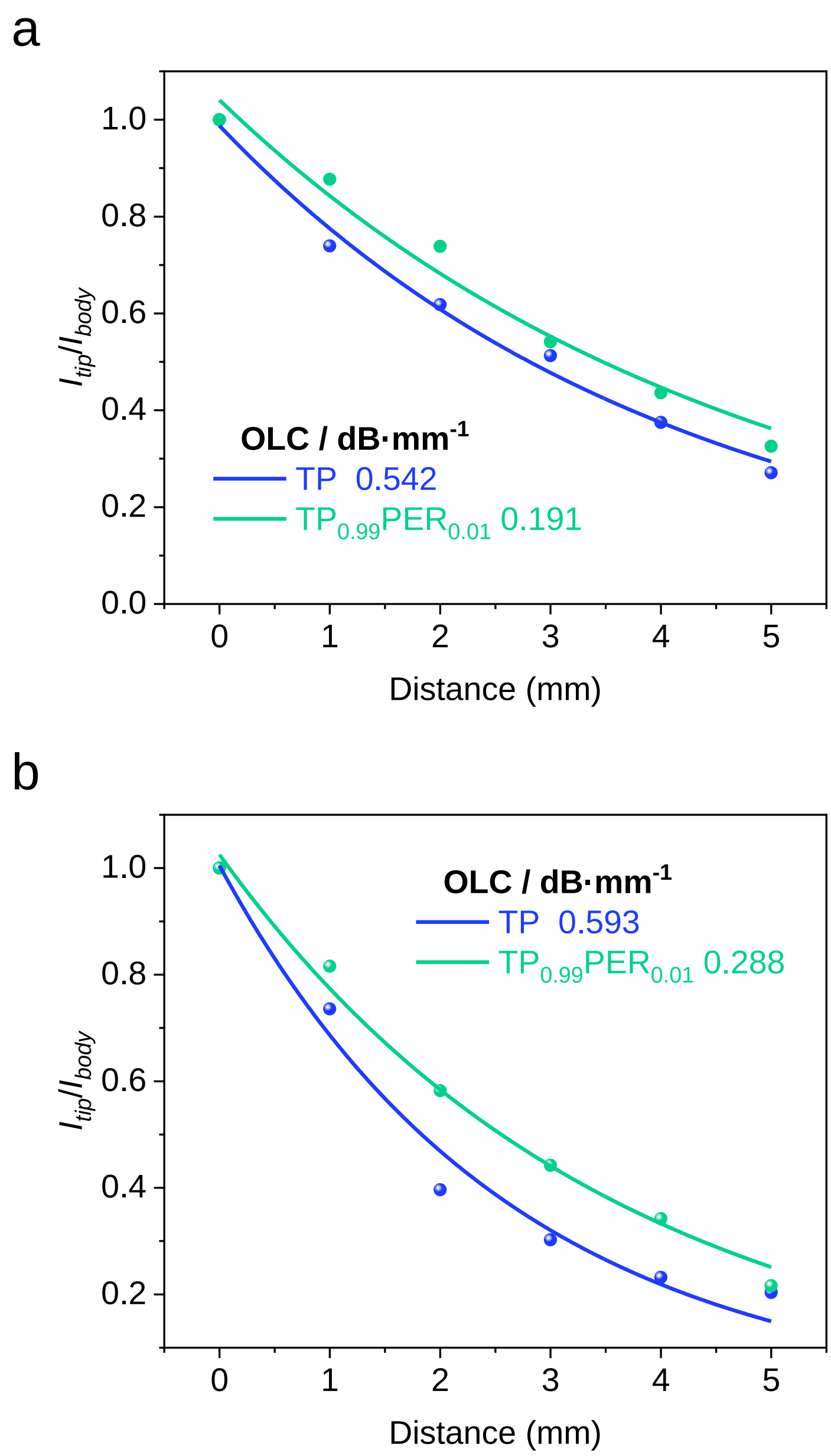

Figure S35. The I_{tip}/I_{body} decays of TP and $TP_{0.99}PER_{0.01}$ crystals in the straight (a), bending (b) states at room temperature.

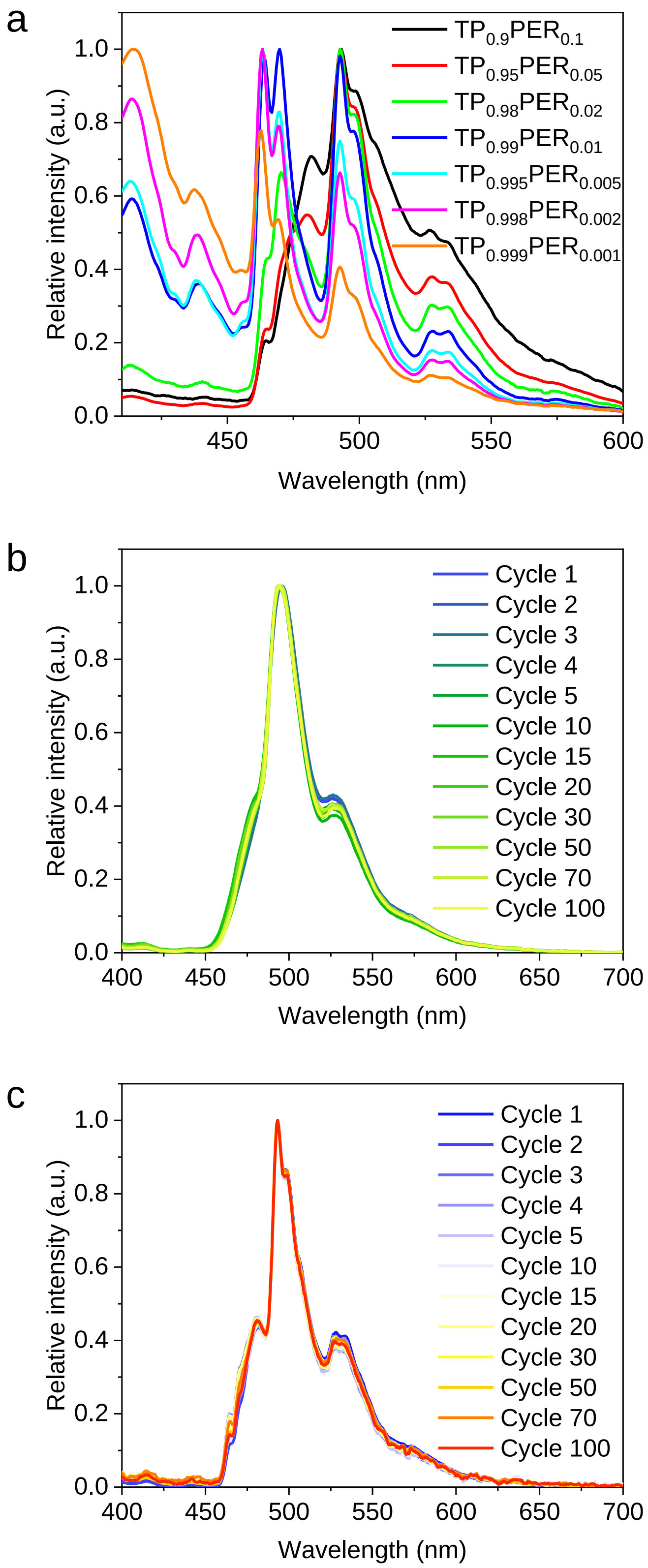

Figure S36. (a) The photoluminescence spectra of TP, PER and alloy crystals under 77 K. (b, c) The photoluminescence spectra of alloy single crystals during repeated cooling–heating cycles (b, 298 K; c, 77 K).

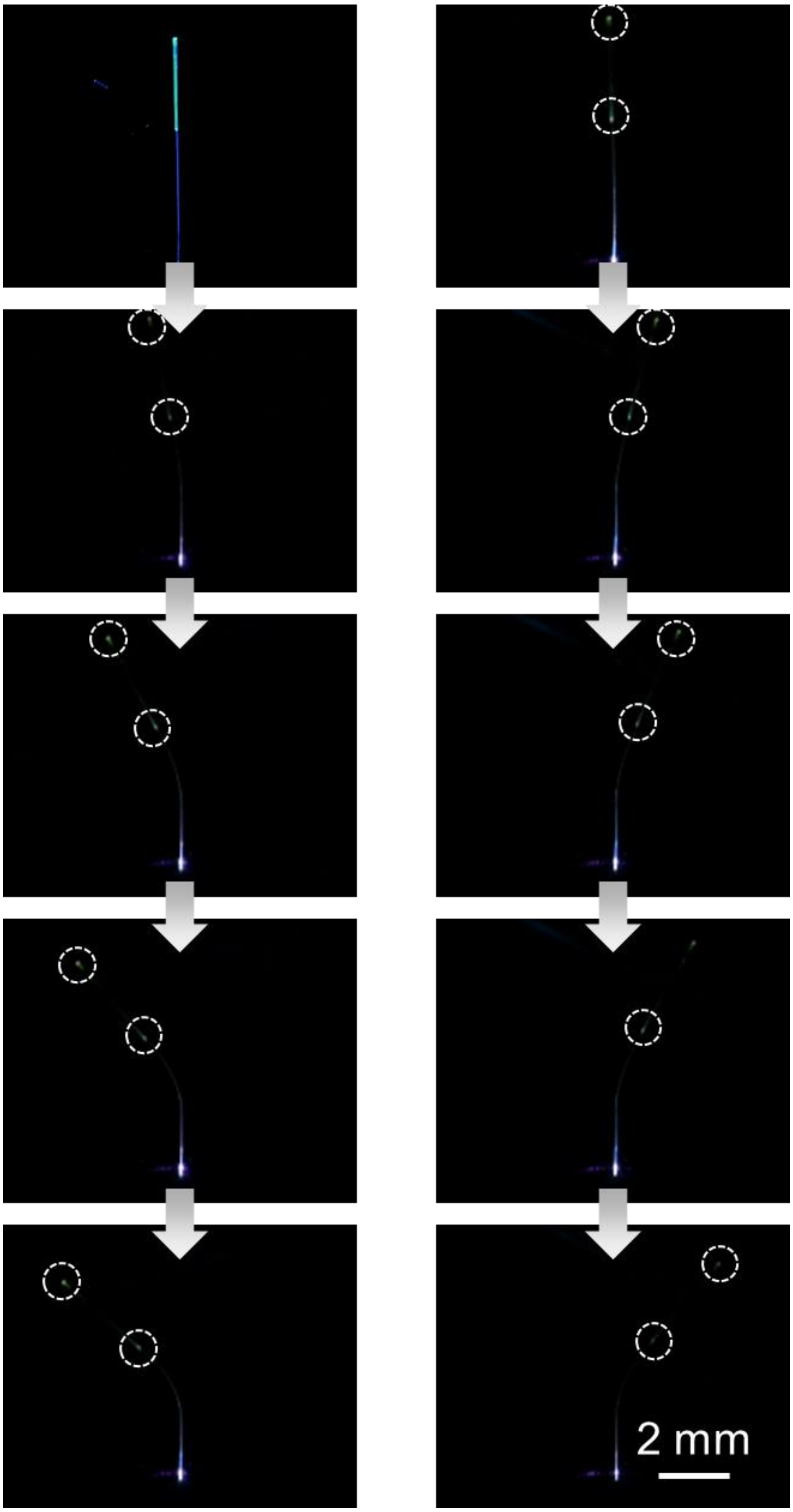

Figure S37. Photoluminescence images of optical signal transmission in bent heterostructures under 365 nm UV excitation.

4. Supplementary Tables

Table S1. Crystallographic data for TP, PER and $TP_{0.9}PER_{0.1}$

	**TP**	**TP**	**PER**	**$TP_{0.9}PER_{0.1}$**	**$TP_{0.9}PER_{0.1}$**
CCDC Number	2389555	2389556	1546182	2552119	2552120
Formula	$C_{18}H_{12}$	$C_{18}H_{12}$	$C_{20}H_{12}$	$C_{18}H_{12}$	$C_{18}H_{12}$
Temperature / K	100	298	173	100	298
Formula weight	228.28	228.28	252.30	228.28	228.28
Crystal system	orthorhombic	orthorhombic	monoclinic	orthorhombic	orthorhombic
Space group	$P2_12_12_1$	$P2_12_12_1$	$P2_1/c$	$P2_12_12_1$	$P2_12_12_1$
a / Å	5.2667(3)	5.2752(3)	10.2735(18)	5.2639(3)	5.2702(3)
b / Å	12.8822(7)	13.1795(8)	10.8005(12)	12.8914(4)	13.1718(8)
c / Å	16.6351(9)	16.7642(10)	11.1744(16)	16.6385(7)	16.7469(9)
α / °	90	90	90	90	90
β / °	90	90	100.880(12)	90	90
γ / °	90	90	90	90	90
Volume / $Å^3$	1128.64(11)	1165.52(12)	1217.6(3)	1129.07(9)	1162.54(12)
Z	4	4	4	4	4
Density / (g cm^{-3})	1.343	1.332	1.376	1.343	1.304
F_{000}	480	480	528	480	480
h_{min}, h_{max}	7, -7	7, -7	12, -11	6, -5	6, -5
k_{min}, k_{max}	17, -17	18, -18	12, -12	16, -16	16, -16
l_{min}, l_{max}	23, -23	23, -23	13, -13	20, -20	20, -20
R_{all}, R_{obs}	0.0610, 0.0435	0.0450, 0.0355	0.1129, 0.0361	0.0460, 0.0398	0.0873, 0.0511
$wR_{2,all}$, $wR_{2,obs}$	0.1255, 0.2011	0.0907, 0.0959	0.0677, 0.0635	0.1007 0.1081	0.0960, 0.1114

Table S2. Fluorescence lifetime and fluorescence quantum yield of TP, PER and alloys

	**TP**	**$TP_{0.999}PER_{0.001}$**	**$TP_{0.998}PER_{0.002}$**	**$TP_{0.995}PER_{0.005}$**	**$TP_{0.99}PER_{0.01}$**	**$TP_{0.98}PER_{0.02}$**	**$TP_{0.95}PER_{0.05}$**	**$TP_{0.9}PER_{0.1}$**	**PER**
τ_1 [ns]	2.81	6.62	6.46	6.92	7.39	6.68	7.49	6.50	17.76
τ_2 [ns]	21.92	41.86	39.69	37.22	35.29	20.79	25.10	17.57	37.09
FRET		0.63	0.64	0.61	0.58	0.62	0.58	0.63	
Φ_F	0.5794	0.6712	0.6236	0.6903	0.7021	0.7434	0.7210	0.6591	0.0508
k_r [10^8 s^{-1}]	0.2061	0.1014	0.0965	0.0997	0.0950	0.1113	0.0962	0.1015	0.0029
k_{nr} [10^8 s^{-1}]	0.1496	0.0497	0.0582	0.0447	0.0403	0.0384	0.0372	0.0525	0.0535

Table S3. Elastic modulus and hardness of TP, PER and alloys

**TP**		$\mathbf{TP_{0.99}PER_{0.01}}$		$\mathbf{TP_{0.98}PER_{0.02}}$		$\mathbf{TP_{0.95}PER_{0.05}}$		$\mathbf{TP_{0.9}PER_{0.1}}$		**PER**	
*E*r (GPa)	*H* (GPa)	*E*r (GPa)	*H* (GPa)	*E*r (GPa)	*H* (GPa)	*E*r (GPa)	*H* (GPa)	*E*r (GPa)	*H* (GPa)	*E*r (GPa)	*H* (GPa)
4.540	0.223	7.705	0.228	8.090	0.227	9.089	0.273	11.636	0.383	12.175	0.378
4.476	0.207	7.781	0.243	7.863	0.226	8.444	0.266	11.392	0.387	12.237	0.388
4.527	0.206	7.821	0.230	7.899	0.216	9.155	0.287	11.370	0.374	12.130	0.390
4.679	0.219	7.760	0.220	8.384	0.243	8.943	0.275	11.655	0.367	12.006	0.394
5.021	0.219	8.040	0.246	8.247	0.240	9.244	0.288	11.568	0.382	12.100	0.403

References

(1) Sheldrick, G. SHELXT - Integrated space-group and crystal-structure determination. *Acta Crystallogr. A* **2015**, *71* (1), 3-8.

(2) Hubschle, C. B.; Sheldrick, G. M.; Dittrich, B. ShelXle: a Qt graphical user interface for SHELXL. *J. Appl. Crystallogr.* **2011**, *44* (6), 1281-1284.

(3) Oliver, W. C.; Pharr, G. M. An improved technique for determining hardness and elastic modulus using load and displacement sensing indentation experiments. *J. Mater. Res.* **1992**, *7* (6), 1564-1583.

(4) Spackman, P. R.; Turner, M. J.; McKinnon, J. J.; Wolff, S. K.; Grimwood, D. J.; Jayatilaka, D.; Spackman, M. A. CrystalExplorer: a program for Hirshfeld surface analysis, visualization and quantitative analysis of molecular crystals. *J. Appl. Crystallogr.* **2021**, *54* (3), 1006-1011.

(5) Turner, M. J.; Thomas, S. P.; Shi, M. W.; Jayatilaka, D.; Spackman, M. A. Energy frameworks: insights into interaction anisotropy and the mechanical properties of molecular crystals. *Chem. Commun.* **2015**, *51* (18), 3735-3738.

(6) Lu, T.; Chen, Q. Independent gradient model based on Hirshfeld partition: A new method for visual study of interactions in chemical systems. *J. Comput. Chem.* **2022**, *43* (8), 539-555.

(7) Gaussian 16, Revision A.03, M. J. Frisch, G. W. Trucks, H. B. Schlegel, G. E. Scuseria, M. A. Robb, J. R. Cheeseman, G. Scalmani, V. Barone, G. A. Petersson, H. Nakatsuji, X. Li, M. Caricato, A. V. Marenich, J. Bloino, B. G. Janesko, R. Gomperts, B. Mennucci, H. P. Hratchian, J. V. Ortiz, A. F. Izmaylov, J. L. Sonnenberg, D. Williams-Young, F. Ding, F. Lipparini, F. Egidi, J. Goings, B. Peng, A. Petrone, T. Henderson, D. Ranasinghe, V. G. Zakrzewski, J. Gao, N. Rega, G. Zheng, W. Liang, M. Hada, M. Ehara, K. Toyota, R. Fukuda, J. Hasegawa, M. Ishida, T. Nakajima, Y. Honda, O. Kitao, H. Nakai, T. Vreven, K. Throssell, J. A. Montgomery, Jr., J. E. Peralta, F. Ogliaro, M. J. Bearpark, J. J. Heyd, E. N. Brothers, K. N. Kudin, V. N. Staroverov, T. A. Keith, R. Kobayashi, J. Normand, K. Raghavachari, A. P. Rendell, J. C. Burant, S. S. Iyengar, J. Tomasi, M. Cossi, J. M. Millam, M. Klene, C. Adamo, R. Cammi, J. W. Ochterski, R. L. Martin, K. Morokuma, O. Farkas, J. B. Foresman, and D. J. Fox, Gaussian, Inc., Wallingford CT, 2016.

(8) Lu, T. A comprehensive electron wavefunction analysis toolbox for chemists, Multiwfn. *Chem. Phys.* **2024**, *161* (8), 082503.

(9) Kresse, G.; Furthmüller, J. Efficient iterative schemes for ab initio total-energy calculations using a plane-wave basis set. *Phys. Review B* **1996**, *54* (16), 11169-11186.

(10) Kresse, G.; Joubert, D. From ultrasoft pseudopotentials to the projector augmented-wave method. *Phys. Review B* **1999**, *59* (3), 1758-1775.

(11) Perdew, J. P.; Burke, K.; Ernzerhof, M. Generalized Gradient Approximation Made Simple. *Phys. Review Letters* **1996**, *77* (18), 3865-3868.

(12) Grimme, S.; Ehrlich, S.; Goerigk, L. Effect of the damping function in dispersion corrected density functional theory. *J. Comput. Chem.* **2011**, *32* (7), 1456-1465.

(13) Humphrey, W.; Dalke, A.; Schulten, K. VMD: Visual molecular dynamics. *J. Mol. Graph.* **1996**, *14* (1), 33-38.
